# The modulation of leaf metabolism plays a role in salt tolerance of *Cymodocea nodosa* exposed to hypersaline stress in mesocosms

**DOI:** 10.3389/fpls.2015.00464

**Published:** 2015-06-26

**Authors:** Amalia Piro, Lázaro Marín-Guirao, Ilia A. Serra, Antonia Spadafora, José M. Sandoval-Gil, Jaime Bernardeau-Esteller, Juan M. R. Fernandez, Silvia Mazzuca

**Affiliations:** ^1^Laboratorio di Biologia e Proteomica Vegetale, Dipartimento di Chimica e Tecnologie Chimiche, Università della CalabriaRende, Italy; ^2^Spanish Institute of Oceanography, Oceanographic Centre of MurciaMurcia, Spain

**Keywords:** seagrasses, leaf proteomics, hypersaline, mesocosm

## Abstract

Applying proteomics, we tested the physiological responses of the euryhaline seagrass *Cymodocea nodosa* to deliberate manipulation of salinity in a mesocosm system. Plants were subjected to a chronic hypersaline condition (43 psu) to compare protein expression and plant photochemistry responses after 15 and 30 days of exposure with those of plants cultured under normal/ambient saline conditions (37 psu). Results showed a general decline in the expression level of leaf proteins in hypersaline stressed plants, with more intense reductions after long-lasting exposure. Specifically, the carbon-fixing enzyme RuBisCo displayed a lower accumulation level in stressed plants relative to controls. In contrast, the key enzymes involved in the regulation of glycolysis, cytosolic glyceraldehyde-3-phosphate dehydrogenase, enolase 2 and triose-phosphate isomerase, showed significantly higher accumulation levels. These responses suggested a shift in carbon metabolism in stressed plants. Hypersaline stress also induced a significant alteration of the photosynthetic physiology of *C. nodosa* by means of a down-regulation in structural proteins and enzymes of both PSII and PSI. However we found an over-expression of the cytochrome b559 alpha subunit of the PSII initial complex, which is a receptor for the PSII core proteins involved in biogenesis or repair processes and therefore potentially involved in the absence of effects at the photochemical level of stressed plants. As expected hypersalinity also affects vacuolar metabolism by increasing the leaf cell turgor pressure and enhancing the up-take of Na^+^ by over-accumulating the tonoplast specific intrinsic protein pyrophosphate-energized inorganic pyrophosphatase (H(+)-PPase) coupled to the Na^+^/H^+^-antiporter. The modulation of carbon metabolism and the enhancement of vacuole capacity in Na^+^ sequestration and osmolarity changes are discussed in relation to salt tolerance of *C. nodosa*.

## Introduction

Seagrasses are marine plants that have successfully colonized the infralittoral bottoms of tropical and temperate coasts around the world representing one of the most ecologically relevant marine coastal habitats (Kuo and Den Hartog, 2000). Although this ecological group of plants is particularly adapted to live completely submersed in a saline medium, their abundance and distribution are determined by critical environmental factors including salinity (Montague and Ley, 1993; Adams and Bate, 1994). The physiological capacity of seagrasses to tolerate salinity changes is species specific and closely related to the salinity regime of the environments in which they grow (Touchette, 2007). This suggests that particular adaptations acquired along their evolution to live submerged in the marine environment are closely related with their current ecological and biological attributes, thus determining their ability to cope with salinity fluctuations. In this regard, C*ymodocea nodosa*, which is a seagrass species inhabiting open coastal waters with stable saline regimes but also hyper-saline lagoons and estuaries with fluctuant salinities, has been considered as an euryhaline species with a higher capacity to tolerate salinity changes than other seagrass species naturally living under a narrower range of salinities (e.g., *Posidonia oceanica*; Tyerman, 1989; Kuo and Den Hartog, 2000; Procaccini et al., 2003; Boudouresque et al., 2009; Sandoval-Gil et al., 2012).

Despite its central role in seagrass ecology, basic knowledge about salinity adaptation and about the specific tolerance mechanisms to increments in seawater salinity is relatively scarce in relation to other issues of seagrass biology and physiology (Touchette, 2007). A key need of physiological studies on seagrasses is the maintenance of plants under controlled mesocosm systems able to simulate the effect of the stress factor of interest (e.g., salinity) and isolate it from the variability of other key factors than can confound or mask the specific responses to the selected stress factor (Coors et al., 2006). In fact, recent ecophysiological studies performed on laboratory mesocosm systems have produced significant new knowledge about the physiological adaptations of Mediterranean seagrass species (mainly *P. oceanica* and *C. nodosa*) to hypersaline stress. Thus, these studies have confirmed that *C. nodosa* is more tolerant to salinity increases than the stenohaline *P. oceanica*. Indeed, several biological attributes of *C. nodosa*, for instance high leaf osmolyte content that confers osmoprotection and high photosynthetic plasticity that allow to balance the plant carbon budget (Marín-Guirao et al., 2011; Sandoval-Gil et al., 2012), have been interpreted as adaptive advantages in its higher resistance to chronic salinity increments. Besides, *C. nodosa* has shown complex responses at the metabolic, physiological and morphological levels that have allowed the species to counterbalance the effects and alterations generated by hypersalinity. These effects include photosynthetic and respiratory alterations, modifications of plant water relations and changes to the metabolite contents of the leaf (Sandoval-Gil et al., 2012, 2014).

Despite this growing physiological background, the application of proteomics is a relatively new tool to be applied to salt response in seagrasses (Serra et al., 2013). Molecular approaches have been recently applied to seagrasses to understand the molecular bases of stress responses, resilience and acclimation to low light (Mazzuca et al., 2009, 2013; Serra and Mazzuca, 2011; Serra et al., 2012; Dattolo et al., 2013, 2014). From these approaches it is possible to recognize new tools that might deserve the designation of “early warning” markers for environmental stresses. A combination of physiology, genomics and proteomics has also recently provided experimental evidence about the induction of specific proteins related to the osmotic stability of *P. oceanica* leaves exposed to salt stress (Serra et al., 2013). Therefore, the combination of molecular techniques and physiological analyses in experiments with plants maintained under controlled mesocosm conditions will represent an effective approach to achieve significant progress in understanding the intrinsic mechanisms of different species of marine plants to cope with hypersaline stress.

On that basis, in this study we used a mesocosm system to expose *C. nodosa* plants to a deliberate increase in seawater salt concentration in order to evaluate how the species modulate their protein expression during the course of the exposure, and how this modulation is linked with their physiological status. To this end, we combined physiological measurements, at the level of plant water relations and plant photochemistry, with a proteomic approach to highlight the molecular adaptation of *C. nodosa* to saline increments. We also took advantage of recent physiological studies on the tolerance of *C. nodosa* to salinity increases to shed light on the possible molecular mechanisms underlying the physiological responses and alterations previously observed in the species.

## Materials and Methods

### Field Plant Sampling and Experimental Design

**FIGURE 1:**
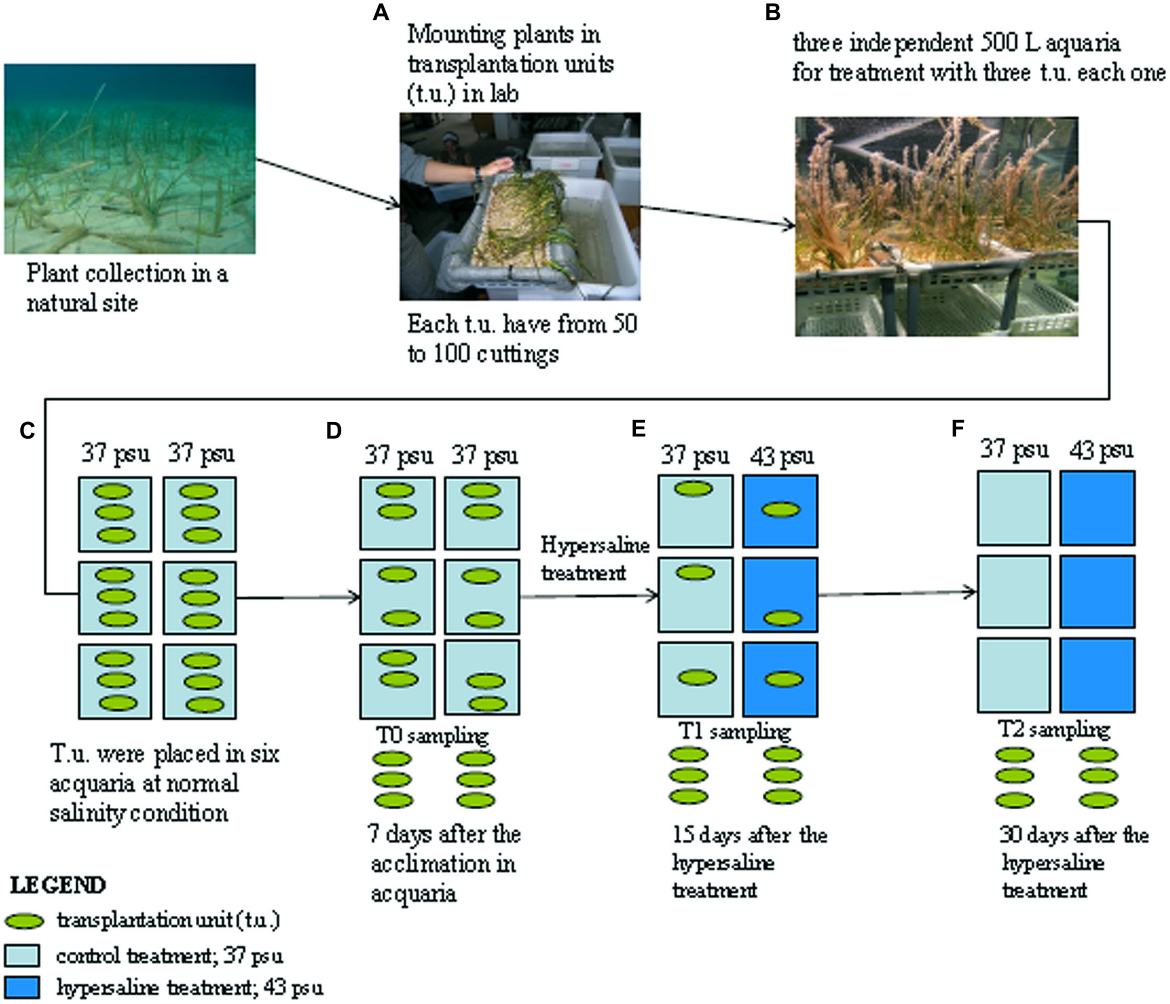
**Schematic representation of the experimental design.** *Cymodocea nodosa* cuttings sampled from the sea are grouped in transplantation units **(A)** and transferred in the aquaria **(B)** under normal salinity conditions **(C)** first sampling occurred after 7 days of acclimation in the aquaria and referred as control samples **(D)**; after this period three aquaria were treated with hypersaline water and plants have been sampled after 15 days **(E)** and 30 days **(F)**. See the text for details.

*Cymodocea nodosa* cuttings (i.e., rhizome apical segments composed of at least 20 connected vertical shoots) were collected by SCUBA divers in a shallow bed (5–6 m deep) located in Isla Plana (Murcia, Spain). Plants were transported under controlled temperature to the laboratory in less than 4 h and rapidly transplanted in the mesocosms. Plant cuttings were fixed onto a grid and mounted in plastic baskets (22 cm × 40 cm base and 10 cm height) filled with pre-washed sediments to a final plant density of 50–60 shoots basket^-1^; each basket represents a transplantation unit (t.u.) and three of them were arranged in each tank of the mesocosm system (**Figures 1A,B**).

The mesocosm system consists of six independent 500-L tanks each with its own source of illumination provided by a 400 W lamp (Aqua Medic Aqualight -400). The sea water used to fill the mesocosm was collected in a nearby pristine open water area. A detailed description of the system can be found in Marín-Guirao et al. (2011) and Sandoval-Gil et al. (2012). This system is able to maintain healthy plants with survival rates at 100% for several months, long enough to achieve the objectives of the experiment (e.g., Marín-Guirao et al., 2013; Sandoval-Gil et al., 2014).

The t.u. were maintained during 1-week acclimation period at 22°C, 37 psu and 300 μmol quanta m^-2^ s^1^ measured on the leaf tips on a 12 h/12 h light/dark cycle (i.e., 12.96 mol quanta m^-2^ day^-1^) according to mean environmental conditions experienced by the donor population during the experimental period (**Figure 1C**). After acclimation and before the application of the experimental treatments (T0), 2 g of fresh leaves were randomly sampled from t.u. collecting shoots from one t.u. of each tank (**Figure 1D**). Then, salinity was increased up to 43 psu in three of the tanks by adding high quality marine salt (Seachem^®^) as previously described (Marín-Guirao et al., 2011), with all other parameters remaining unchanged. Plants were sampled again from both the control and the hypersaline treatments after 15 and 30 days of hypersaline exposure (**Figures 1E,F**). At each sampling time three different replicates were processed each composed of the material from one t.u. of each independent tank. Mature non-damaged leaves were selected for the analysis to avoid old and necrotic tissues. Collected leaf tissue were washed in sea water, gently cleaned with a razor to remove epiphytes, washed again in distilled water to remove salt from the leaf epidermis, frozen in liquid nitrogen and stored at -80°C for further proteomic analysis.

### Physiological Measurements

Chlorophyll *a* fluorescence emissions were performed using a diving-PAM portable fluorometer (Walz, Germany). Measurements were done on plants in the mesocosm adapted to darkness overnight (i.e., before switching on the illumination system) to ensure full oxidation of the reaction centers and primary electron acceptors. This allowed us to calculate the maximum quantum yield of the photosystem II (PSII; Fv/F), which represents a measure of the maximum photochemical efficiency of the PSII (Schreiber, 2004). A more detailed description of the measurements can be found in Sandoval-Gil et al. (2012). Measurements were taken before the application of the hypersaline treatment and after 7, 15, and 30 days of hypersaline exposure. In each sampling time measurements were performed on three randomly selected shoots from each t.u. and averaged per tank to have a final number of three replicates (i.e., one replicate tank^-1^) per treatment.

Leaf-water relation variables (i.e., water potential Ψw, osmotic potential Ψp and turgor pressure P) of *C. nodosa* plants were analyzed before the application of the hypersaline treatment and after 15 and 30 days of hypersaline exposure. Three different shoots from each tank and sampling time were employed for the measurements of leaf-tissue osmolality (mmol kg^-1^ FW) using a Wescor Vapor Pressure Osmometer 5520 (Logan, Utah). Measurements were averaged per tank to have three replicates per experimental condition and sampling time. Osmolality was measured both in fresh and frozen blotted leaf segments to obtain Ψw and Ψp for each shoot, and expressed in megapascals (MPa), using the van’t Hoff relation (Tyerman, 1989). The leaf-tissue turgor pressure (P) was then calculated as the absolute difference between Ψw and Ψp. Ambient seawater osmolality was also determined by measurements of seawater in 6.5 mm sample disks, following the standard protocol (Wescor Inc.).

### Extraction and Purification of Total Protein from Leaves

Leaf tissues of marine plants are considered to be recalcitrant to the common protocols based on the aqueous buffers extraction because they are rich in secondary metabolites, such as phenols, disaccharides, lipids, that severely interfere with protein extraction and purification (Wang et al., 2006; Spadafora et al., 2008). Here we applied a multistep procedures that precipitated proteins prior to their extraction in a phenol phase. For each extraction 1.4 g of leaves were crushed in a mortar in liquid nitrogen until a fine powder was obtained. This powder was divided into 2 ml microfuge tubes; a volume of 10% TCA in acetone was added and centrifuged at 13000 rpm for 5 min at 4°C. Subsequently, four washes were performed in 80% acetone in water. TCA is a strong acid and precipitates the protein when it is still in the tissue powder; at the same time, phenols, sugars, and other soluble molecules are washed out by the TCA solution while the hydrophobic molecules are dissolved in the acetone. After centrifugation the pellet containing the precipitated proteins was dried at room temperature. The powder was collected in microfuge tubes and kept at -80°C for subsequent analysis or immediately processed for phenolic phase extraction of proteins.

Approximately 0.1 g of powdered tissue was dissolved in 0.8 ml of phenol (buffered with Tris-HCL, pH 8.0, Sigma, St. Louis, MO, USA) and 0.8 ml of SDS buffer (30% sucrose, 2% SDS, 0.1 M Tris -HCl, pH 8.0, 5% 2-mercaptoetanol) in a 2 ml microfuge tube. The samples were vortexed for 30 s and centrifuged at 13000 rpm for 5 min to allow proteins to solubilise in the phenol phase. The phenol phase was mixed with five volumes of 0.1 M ammonium acetate in cold methanol, and the mixture was stored at -20°C for 30 min to precipitate proteins. Proteins were collected by centrifugation at 13000 rpm for 5 min. Two washes were performed with 0.1 M ammonium acetate in cold methanol, and two with cold 80% acetone, and centrifuged at 13000 rpm for 7 min. The final pellet containing purified protein was dried and dissolved in Laemmli 1 DE separation buffer over-night. Proteins were then quantified by the Bradford assay. Protein yield was measured as mg of protein per g fresh tissue weight in five independent biological replicates at each time and treatment.

### Electrophoresis of Leaf Proteins, Protein In-Gel Digestion, and Mass Spectrometry Analyses

A gel was prepared at a concentration of 10% acrylamide/bisacry-lamide, according to the method of Laemmli (1970). The ratio of acrylamide/bisacrylamide was 12.5% in the running gel and 6% in the stacking gel. The samples were heated for 5 min at 100°C before being loaded on the gel. The electrophoretic run was carried out at 60 mA for the stacking gel and 120 mA in the running gel at constant power of 200 V. The electrophoresis ran for an average of 1 h and 15 min. The gels were stained with Coomassie Blue over-night and subsequently destained with several changes of destaining solution (45% methanol, 10% acetic acid).

**FIGURE 2:**
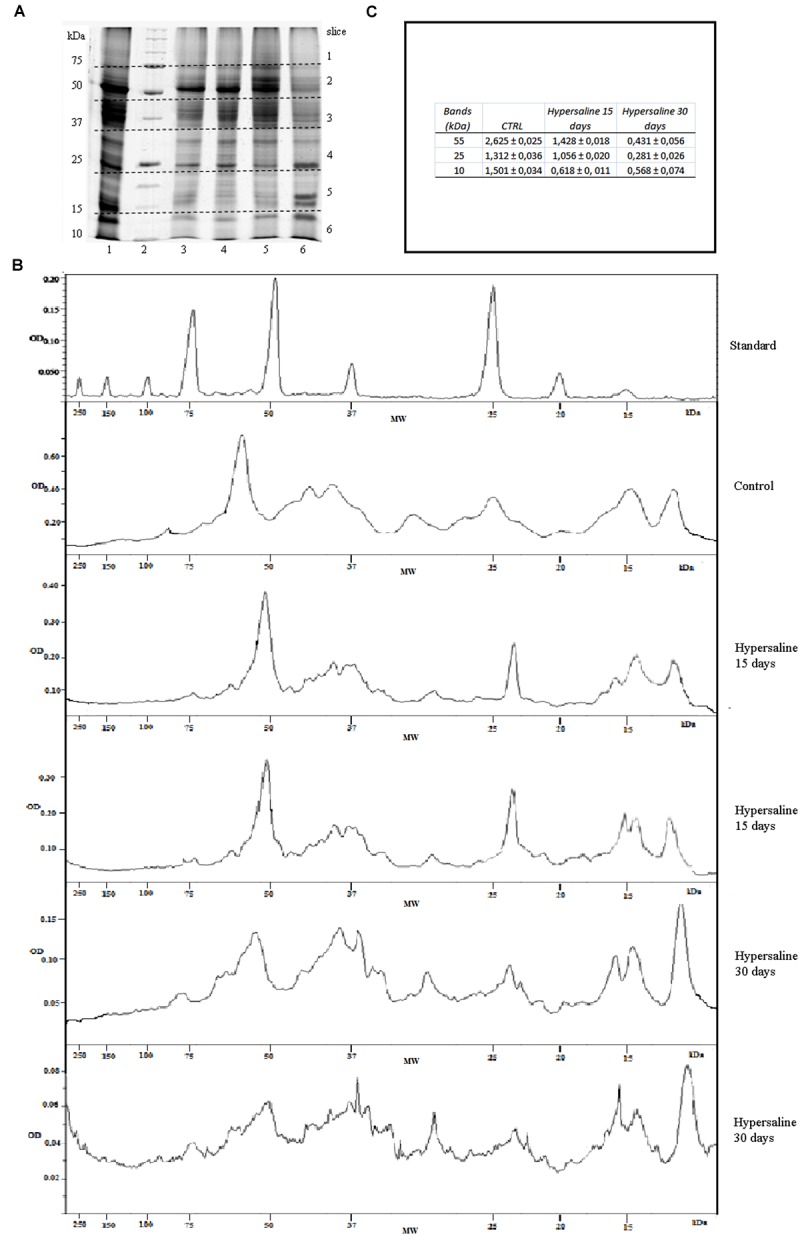
**(A)** SDS-PAGE of proteins extracted from leaf tissue of *C. nodosa* under normal (37 psu) and hypersaline (47 psu) conditions in mesocosm. Lanes 1, 2, 3 replicates from normal saline condition after 7 days of acclimation; lanes 4, 8) proteins standard mix (Bio-Rad, range from 200 to 10 kDa); lanes 5, 6, 7) replicates after 15 days hypersaline condition; lanes 9, 10, 11) replicates after 30 days hypersaline condition. **(B)** Examples of chromatograms of the optical densities (OD) from the lines of protein standard, control sample and hypersaline treatments in a same SDS-PAGEs as a function of molecular weights (kDa). **(C)** Protein concentration at the bands of 55, 25, and 10 kDa in the control samples and after 15 and 30 days hypersaline treatments.

Digitalized images of the stained SDS-PAGEs were analyzed by the Quantity One 1-D Analysis Software (Bio-Rad) to measure the optical densities at each lane of all biological replicates among the treatments. The amount of protein at bands of 55, 25, and 10 kDa was done using the marker reference bands at 75, 50, and 25 kDa that contained 150, 750, and 750 ng of proteins respectively (**Figures 2A–C**). Each lane of the same SDS-PAGE were divided in six slices from 200 to 10 kDa and manually excised from the gel, cut in small pieces, *S*-alkylated and digested overnight at 37°C with trypsin (Wilm et al., 1996). Digested peptides were extracted from the gel slices with 25 mM NH_4_HCO_3_/ACN 1:1 (v/v) and the peptide mixtures were concentrated by evaporation in a vacuum centrifuge. The gel slices were then treated with 5% (v/v) formic acid in acetonitrile (ACN). After drying, the tryptic peptides were analyzed by tandem mass spectrometry by means of liquid chromatography(LC-MS/MS) using a high resolution mass spectrometer LTQ- Orbitrap XL (Thermo Fisher Scientific). The chromatographic separations were carried out on a Waters XBridgeC18 column (300 μM ID × 100 mm in length and 3.5 μm per particle size) using a linear gradient of 5–90% ACN containing 0.1% formic acid with a flow of 4 μL/min, including the regeneration phase, a run lasted about 70 min; microflow conditions were employed in order to obtain more reproducible semi-quantitative data. Full scan MS high resolution spectra (resolution of 30,000) were acquired. Data were acquired in data dependent scan acquisition (DDA) conditions and the MS/MS spectra were acquired in an Ion Trap in low resolution mode to decrease the acquisition scan duty cycle. The most abundant peak was fragmented under dynamic exclusion conditions. In particular, the peak was fragmented two times and maintained in the dynamic exclusion list for 90 s. The acquisitions were undertaken in scanning mode data-dependent MS/MS (with full scan range of 250–1800 m/z).

### Bioinformatics Analysis and Identification of Proteins of *Cymodocea nodosa*

Spectra acquired by LC-MS/MS were used to identify peptide sequences using the open-source system global proteome machine (GPM) engine against the GPM public UniGene (NCBI) on-line plant database^1^. Since the GPM plant database lacks seagrass sequences and considers only few species that belong to *Liliopsida*, a search can lead to reduced peptide matches after mass spectrometry. Thus, spectra acquired by LC-MS/MS were also used to match peptide sequences using X!Tandem software (Fenyö et al., 2010) against a customized database built with a collection of protein sequences from multiple databases. This comprised sequences from seagrasses and other species belonging to *Liliopsida* available in the UniProtKB database and included sequences from *P. oceanica, Zostera marina* and from five EST libraries (Pooc_A, Pooc_B, Zoma_A, Zoma_B, and Zoma_C) collected from the *Dr.Zompo* database (Wissler et al., 2009^2^). In the latter instance, it has been necessary to first create a protein database from the nucleotide sequences as described in Dattolo et al. (2013). The use of all possible reading frames enabled the optimization of peptide identifications. The GPM and !XTandem searches were done in parallel and then results combined to obtain the final outcome. Molecular function and biological processes of each identified protein were obtained from the Gene Ontology (GO) website to assign metabolic pathways for each identified protein.

### Semi-Quantitative Analysis of Identified Proteins

Quantitation of the identified proteins was performed by spectral counting (Zhang et al., 2010). Differences in spectral counts are identified by applying the Normalized Spectral Abundance Factor (Zybailov et al., 2006). The false discovery rate (FDR) was calculated from the matched spectra against a reverse peptide database according to Elias and Gygi (2007); the threshold for protein FDR corresponded to 1%.

Each m/z ratio was subjected to a statistical discriminate analysis using the XCMS algorithm. Basically it can be summarized as a *t-*test peak per peak. Spectral count were undertaken only on statistically validated spectra to increase its accuracy. Consequently, it was used for quantitation comparisons. A peptide with less than two matches was discarded. Three biological replicates for each treatment was used for quantitative analyses. The missing values were considered to be undetectable and assumed they were under the limit of detection, but present. Thus when they were undetectable, a zero value was attributed and they were considered in the statistical calculation. Data from repeated measurements are shown as mean ± SD or ± SE. Comparison of differences among the groups was carried out using a Student’s *t-*test. Significance was defined as *p* ≤ 0.05.

## Results

### Physiological Analyses

**FIGURE 3:**
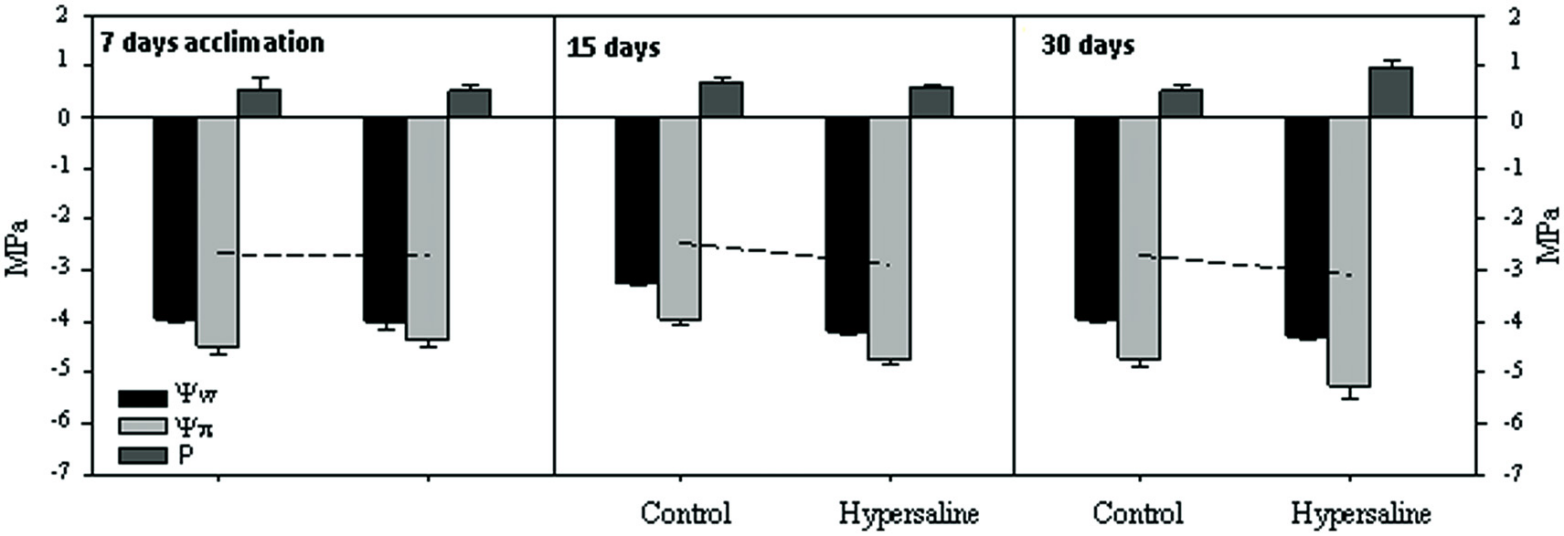
**Leaf-water relations variables (water potential Ψw, osmotic potential Ψp, and turgor pressure P) expressed in megapascals (MPa), of *C. nodosa* plants under normal and after 15 and 30 days of hypersaline exposure.** The leaf-tissue turgor pressure (P) was then calculated as the absolute difference between Ψw and Ψp. Measurements are the average of three replicates per experimental condition and sampling time.

All along the course of the experiment, plants subjected to hypersaline showed similar (*p* > 0.05) levels of maximum photochemical efficiency of PSII (Fv/Fm) to that of control plants, with mean values within the range 0.763–0.777. At the end of the experimental period, the potential photochemical efficiency of plants under cultured conditions in the mesocosm system (i.e., 0.775 ± 0.004) was similar to that measured in the field, natural population (0.780 ± 0.02). After 7 days acclimation in the mesocosm and before the application of the hypersaline treatment, water relation parameters did not change in either tank (**Figure 3**). Once under hypersaline conditions, *C. nodosa* plants reduced the water potential (Ψw) of their leaves as a response to the reduction in the osmotic potential of seawater caused by the addition of salts. After 30 days of hypersaline exposure the significant (*p* < 0.01) reduction in Ψw was attained through a significant (*p* < 0.05) reduction in the osmotic potential (i.e., Ψπ) of leaves indicating the accumulation of osmolytes, while the osmotic pressure remained unaltered. After an exposure of longer than 30 days, the osmotic potential of leaves further decreased, indicating a higher accumulation of osmolytes. It was at this sampling time that an increase in turgor pressure was observed in leaves of plants exposed to hypersalinity; observed values were on average 90% higher those of control plants.

### Protein Expression

The total amount of protein extracted from *C. nodosa* leaf tissues along the course of the experiment from both the control and hypersaline treatments are shown in **Table 1**. The protein yields decrease about 20 and 30% in hypersaline stressed plants after 15 and 30 days of exposure, respectively. Highly purified proteins from *C. nodosa* leaves are well resolved by SDS-PAGE. As shown in **Figure 2**, the SDS-PAGE pattern of control plants grown for 7 days in the mesocosm revealed a high number of polypeptide bands demonstrating the efficient protein extraction and purity by means of a multistep protocol. A considerable reduction of proteins at 55 and 25 kDa, corresponding to the RuBisCo large subunit and LHCP respectively, was detected in plants exposed to hypersalinity for 15 days with respect to the control (**Figures 2B,C**); overall a reduction in the number of major resolved protein bands occurred after 30 days, with a further decrease of protein at 55 and 25 kDa, while proteins with small molecular weights (10 kDa) increased (**Figures 2B,C**).

**Table 1:** Protein yield from leaf tissues of *Cymodocea nodosa* after 7, 15, and 30 days culture in the mesocosm (control) and after 15 and 30 days of hypersaline treatments.

	Protein yield (mg/g fresh weight)		
Sample	Seven days	Fifteen days	Thirty days
Control	3.80 ± 0.30	3.40 ± 0.31	3.50 ± 0.40
Hypersaline	–	3.04 ± 0.80	2.70 ± 0.82

Values are the main of five independent replicates (±SE).

**FIGURE 4:**
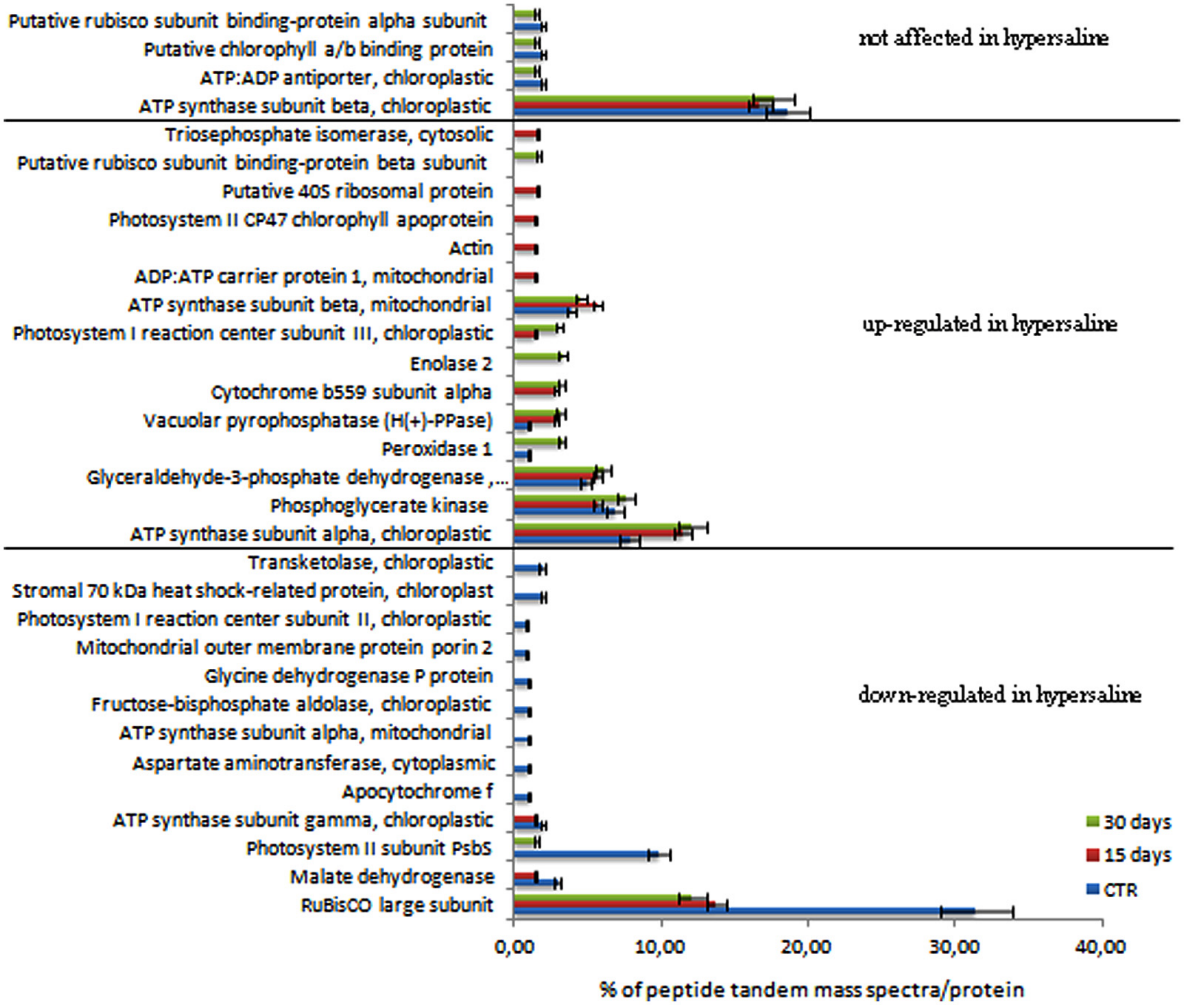
**Semi-quantitative analysis of proteins expressed in *C. nodosa* leaves under normal or under hypersaline condition at 15 and 30 days.** Plants after 7 days acclimation in normal condition are referred as the control samples. Values are the percentage of peptide tandem mass spectra assigned to each protein by means of the spectral counting method. Proteins were clustered according to their expression level in control and hypersaline-treated plants. Mean values (±SE) are calculated among three independent replicates each treatments. See the Supplementary Table S1 for details.

For mass spectrometry analyses, gel pieces were associated in pairs along a molecular weight gradient to compare the protein expression among different sample treatments and durations. Samples collected after 7 days acclimation and before the application of the experimental treatments were used as the control (**Figure 2** lanes 1, 2, 3). For each identified protein the expression level has been calculated using a mean of the spectral count and values were compared among control and hypersaline-treated plants at different times; the results of the analysis are reported in the **Figure 4**. Statistical parameters of each identified protein, number of MS spectra for each peptide assigned to each protein in the control and hypersaline-treated plants are reported in Supplementary Table S1; single peptide sequence assigned to each identified protein and their related accession numbers are reported in Supplementary Table S2.

Despite the LC-MS/MS analysis resulting in an array of MS/MS spectra, many could not be attributed to peptide sequences, even though their fragmentation pattern was typical to that of a peptide (data not shown). Many LC-MS spectra did not match, even after *de novo* sequencing and BLAST searches. Thus it can be concluded that many sequence were likely absent from our databases and this resulted in the lack of associated with a known protein.

**FIGURE 5:**
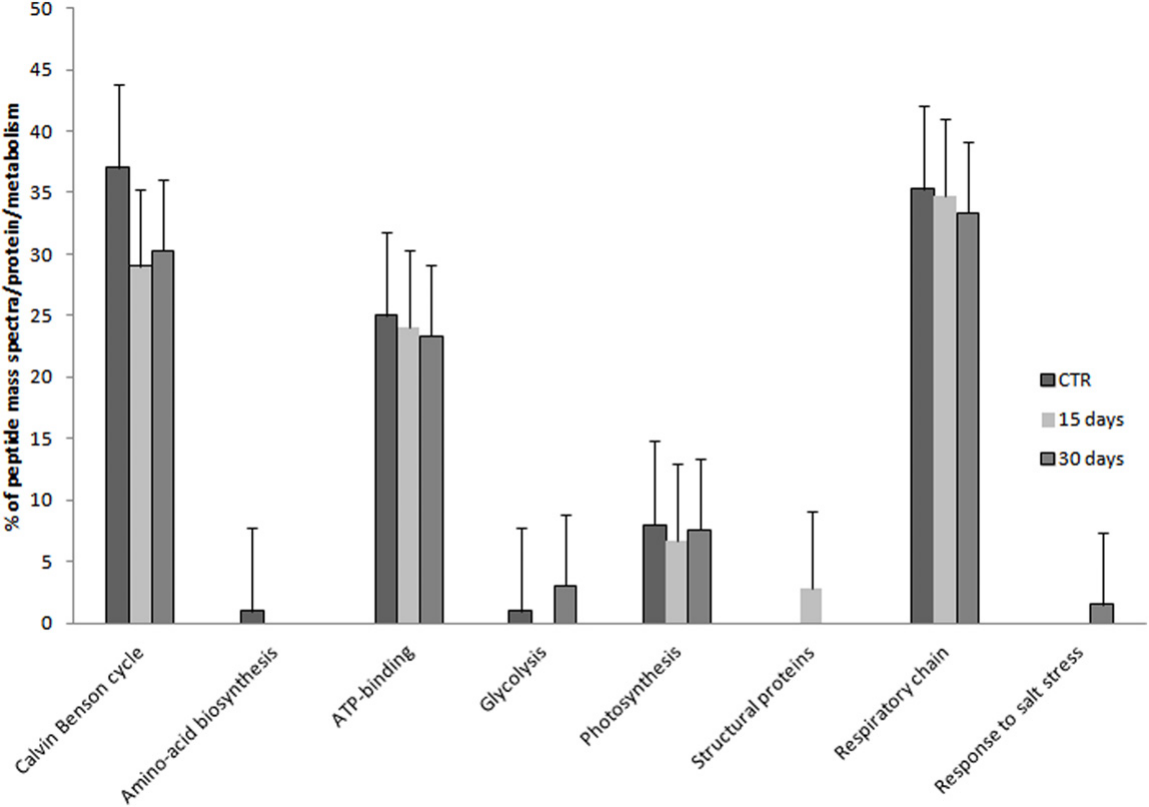
**Expression level of proteins associated as functional groups in *C. nodosa* leaves under normal or under hypersaline condition for 15 and 30 days.** Plants after 7 days acclimation in normal condition are referred as the control samples. Values are the cumulative percentages of tandem mass spectra assigned to each protein belonging to each metabolic category performed using the spectral counting method. Mean values (±SE) are calculated among three biological replicates each treatments. See the Supplementary Table S2 for details.

Among the identified proteins, a set of 32 differentially accumulated proteins was identified in controls vs. hypersaline-treated plants, including a group of proteins whose levels are lower in the hypersaline-treated plants, the PSII subunit PsbS, the RuBisCo large subunit and malate dehydrogenase all appeared down-regulated (**Figure 4**). This group of proteins progressively decreased over the duration of 30 days of hypersaline exposure. After this time, the long lasting exposure resulted also in the chloroplastic ATP synthase subunit gamma and the mitonchondrial ATP synthase alpha subunit being down-regulated in leaves of stressed plants. A second group of proteins showed a high number of spectral counts in the samples under hypersaline treatment; among these are the cytosolic glyceraldehyde-3-phosphate dehydrogenase, the cytochrome b559 subunit alpha (PSII subunit V), enolase 2, peroxidase 1 and the tonoplastic intrinsic protein pyrophosphate-energized inorganic pyrophosphatase (H(+)-PPase). Few proteins displayed lower or no significant variation in their spectral counts according to the treatments (**Figure 4**). Proteins were grouped according to their functional pathways assigned to each protein using GO. In **Figure 5**, the percentage of spectral counts assigned to each identified protein was grouped according to their function. In control plants the proteins involved in Calvin-Benson cycle and the respiratory chain accounted for 60% of total identified proteins; hypersaline treatments effected both these functional classes with a reduction of overall protein content that showed a strong decrease after the long-lasting treatment (30 days). The proteins allocated to photosynthetic metabolism were also drastically reduced by hypersaline conditions after 15 days, while enzymes involved in the glycolytic pathway were unchanged. Differences were not significantly detected in other metabolic pathways.

## Discussion

This research combined, for the first time, proteomic and physiological approaches to assess the effects of hypersalinity on *C. nodosa*. Even if a low number of differentially accumulated proteins were identified because of poor genomic resources in marine plants, results obtained support some of the current and previous physiological responses observed in mesocosm studies and analyses, shedding light on the possible molecular mechanisms underlying the physiological responses and alterations caused by hypersalinity on this seagrass species.

The maximum efficiency of PSII to drive the light absorbed by the leaf pigment matrix into photochemistry (i.e., Fv/Fm) was not altered in *C. nodosa* plants as a consequence of the hypersaline exposure, which is in agreement with previous studies (e.g., Sandoval-Gil et al., 2012, 2014). Interestingly when these plants are exposed to hypersalinity they display an over-expression of the cytochrome b559 alpha subunit in their leaves, together with the down-regulation of other structural proteins of both the PSII and the PSI. The cytochrome b559, which accumulates in the thylakoid membrane even in the absence of other PSII subunits, is considered as a prerequisite for PSII assembly (Morais et al., 1998), and together with protein D2, the PSII initial complex that serves as a receptor for other PSII core proteins during the biogenesis or the PSII repair process (Adir et al., 1990; van Wijk et al., 1997; Müller and Eichacker, 1999; Zhang and Aro, 2002). Therefore both processes, the repair of damaged PSII cores and the biogenesis of new PSII, could be enhanced during the hypersaline exposure to maintain PSII basal activity. The increased accumulation of this protein may also partially explain the absence of alterations at the level of PSII repair cycle previously reported for *C. nodosa* plants chronically stressed by hypersalinity, which contrasts with the alterations detected in the less tolerant species *P. oceanica* (Marín-Guirao et al., 2013). On the other hand, hypersaline-stressed *C. nodosa* plants have previously shown an improved capacity to harvest light as a response to counterbalance photosynthetic inhibition and this could probably be related to the genesis of new PSII.

In spite of the absence of alterations at the level of PSII photochemistry, the hypersaline exposure induced a significant alteration of the photosynthetic physiology of *C. nodosa* by means of a marked down-regulation of the key enzyme involved in photosynthesis, the carbon-fixing enzyme RuBisCo. As suggested by Sandoval-Gil et al. (2012), the reduction in this enzyme is more likely to be the cause of the salt-induced photosynthetic inhibition reported in this species instead of the damage to photosynthetic structures that has been reported for other seagrass species under more severe hypersaline conditions (e.g., Kahn and Durako, 2006; Koch et al., 2007). Indeed, the lower accumulation of the carbon-fixing enzyme RuBisCo is in agreement with Beer et al. (1980) who provided some experimental evidence of RuBisCo activity reduction in epidermic cells of *C. nodosa* in an *in vitro* high salt assay media. Interestingly, these authors also found that the activity of PEPcase (phosphoenolpyruvate carboxylase) increases in *C. nodosa* due to salt stress, potentially conferring the species the ability to compensate for the reduction in carbon assimilation as a consequence of the reduction in RuBisCo activity.

It has been demonstrated that the photosynthetic physiology of *C. nodosa* is significantly altered following exposure to chronic salinity increases, although the nature of the observed photosynthetic inhibition differed depending on the severity of the hypersaline stress, but also on the origin of plants (Sandoval-Gil et al., 2012, 2014). In relation to the intensity of the stress, at salinity levels slightly lower than the one employed here (i.e., 39–41), net photosynthetic rates of stressed plants declined due to a substantial respiratory increase. However, under a similar hypersaline stress level employed here (i.e., 43 psu), the respiration rate of plants was limited, but instead an inhibition of gross photosynthetic rates caused a significant reduction in carbon assimilation of stressed plants.

Regarding the reduction in the respiratory activity, proteomic analyses have revealed an overall down-regulation of both mitochondrial and chloroplastic ATP synthases suggesting a reduction of the oxidative and photoxidative phosphorylation processes that are directly related to respiration and photosynthesis. Interestingly in the same sample, the levels of some chloroplast ATP synthase subunits appeared up-regulated or not affected by treatment; this behavior is also found in other species subjected to long lasting salt stress; Wang et al. (2015) reported that chloroplast ATP synthase levels decrease in abundance under long term salt stress, with beta subunits down-regulated and gamma subunits unaffected with respect to controls, indicating that energy supply was affected. However, the authors limited their discussions of these finding, so there is little evidence to justify such a behavior. Anyway, we speculate that as the beta and gamma subunits are encoded by a chloroplastic and a nuclear gene respectively, timing of transcription and translation might not be synchronous and thus differently regulated (Drapier et al., 2007; Rott et al., 2011). A similar behavior has been found for the mitochondrial ATP synthase subunits under salinity stress leading to the up-regulation of the beta subunit and to the down-regulation of alpha subunit (Srivastava et al., 2009). Overall, these results are consistent with the reduction in ATP production from both photophosphorylation and oxidative phosphorylation, as suggested by the reduced levels of ATP synthase. This could be the reason for respiratory inhibition as a plant response to balance the altered ratio between production and consumption of ATP. This suggests that under NaCl stress conditions the carbon balance switches to favor the inorganic carbon increase in tissue as a response to the decrease in photosynthesis rate. In addition, hypersaline treated-plants showed higher levels of the key enzymes involved in glycolysis, cytosolic glyceraldehyde-3-phosphate dehydrogenase, as well as of other enzymes involved in this metabolic pathway such as enolase 2 and triose-phosphate isomerase. The increased concentration of these compounds in leaf tissues of salt-stressed plants reflect an overall up-regulation of the glucose reduction in leaf cells, and suggest that glycolysis may be balancing the demand for energy by producing ATP in the reduction steps from 1,3- diphosphoglycerate to phosphoglycerate and from phosphoenolpyruvate to pyruvate. This supports the idea that non-structural carbohydrates have a high functional plasticity under hypersaline stress, and together with their role as osmolytes (e.g., Touchette, 2007) they can also be involved in other key metabolic functions, for instance acting as respiratory substrates (Drew, 1978; Bisson and Kirst, 1995). This increased cell respiration, however, can jeopardize plant survival through the consumption of their energetic reserves in the long-term.

*Cymodocea nodosa* is able to maintain its leaf osmotic stability under hypersaline conditions through different dehydration avoidance strategies, including cell-wall hardening and osmoregulatory processes. During osmoregulation, inorganic ions sequestered into vacuoles and cytosolic compatible organic osmolytes (e.g., sugars, proline) have been reported to play a key role in seagrasses (e.g., Koch et al., 2007; Sandoval-Gil et al., 2012, 2014; Marín-Guirao et al., 2013) and must be responsible for the osmotic potential reduction observed in this study. Indeed, the participation of ions in leaf osmotic potential of *C. nodosa* at the early stages (i.e., 1-week) of its acclimation adjustment to hypersalinity has recently been shown to increase, in parallel with increases in salinity (Garrote-Moreno et al., 2014). In this case, after a longer exposure of 1 month we observed a marked increase in pressure of the leaf cell turgor, which is an atypical response previously reported in *P. oceanica* (Sánchez-Lizaso et al., 2008; Ruiz et al., 2009; Marín-Guirao et al., 2013; Sandoval-Gil et al., 2014), as well as in some terrestrial plants and marine algae exposed to salt stress (Behboudian et al., 1986; Kirst, 1989). In agreement, vacuolar metabolism of these hypersaline stressed plants showed signs of being perturbed as reflected by the over-expression of the tonoplast specific intrinsic protein pyrophosphate-energized inorganic pyrophosphatase (H(+)-PPase). This response suggests that vacuoles are engaged in Na^+^ sequestration accordingly with a high capacity of proton pumping and Na^+^ uptake via the Na^+^/H^+^-antiporter, a response previously observed in seagrasses (Muramatsu et al., 2002), halophytes (Debez et al., 2006) and in other higher plants under hypersaline stress (Fukuda et al., 2004; Epimashko et al., 2006). A possible explanation of these responses is the involvement of the *turgor sensing mechanisms*, by which the disturbance of the plasma membrane by turgor increments led to the activation of downstream signaling cascades involved in the osmoacclimation responses (Zimmerman, 1978; Kirst, 1989; Bisson and Kirst, 1995). Nonetheless, there is no reliable explanation for this behavior and further work is needed to determine which are the specific molecular and physiological mechanisms involved and their role in the acclimation capacity of the species to saline increments.

## Conclusion

We have found severe changes in the leaf primary metabolisms due to hypersalinity both at short and long-lasting treatments. Overall, the proteomic analysis revealed that the physiological tolerance of *C. nodosa* to sudden and chronic increases in external salinity is mediated by its capacity to modulate primary metabolism resulting in a new carbon balance combined with efficient Na^+^ sequestration against its electrochemical gradient toward the vacuoles of mesophyll cells. As would be required for cytoplasm protection and suitable osmotic adjustments (Barbourina et al., 2000; Taji et al., 2004). These drastic rearrangements at the level of primary metabolism allowed *C. nodosa* plants to survive for more than 1 month (plant mortality was not detected in the present work) evidence supporting their successful metabolic and physiological adaptation to the selected ionic and osmotic stressful conditions. The absence of mortality at this level of chronic stress have previously been reported for the species under controlled experimentation in mesocosm (Sandoval-Gil et al., 2012) contrary to the population decline observed in field studies associated with constant salinity fluctuations (Garrote-Moreno et al., 2014) or to the synergistic effects of hypersalinity with other products employed in the maintenance of seawater reverse osmosis plants (i.e., metabisulphite; Portillo et al., 2014).

## Conflict of Interest Statement

The authors declare that the research was conducted in the absence of any commercial or financial relationships that could be construed as a potential conflict of interest.

## Footnotes

1 http://plant.thegpm.org/tandem/thegpm_tandem.html

2 http://drzompo.uni-muenster.de/

## References

[B1] Adams J. B. Bate G. C. (1994). The effect of salinity and inundation on the estuarine macrophyte *Sarcocorniaperennis* (Mill.) *A. J. Scott. Aquatic Bot.* 47 341–348. 10.1016/0304-3770(94)90063-9

[B2] Adir N. Shochat S. Ohad I. (1990). Light-dependent D1 protein synthesis and translocation is regulated by reaction center II. Reaction center II serves as an acceptor for the D1 precursor. *J. Biol. Chem.* 265 1256 3–12568. 2197278

[B3] Barbourina O. Leonova T. Shabala S. Newman I. (2000). Effect of sudden salt stress on ion fluxes in intact wheat suspension cells. *Annu. Bot.* 85 759–767. 10.1006/anbo.2000.1136

[B4] Beer S. Shomer-Ilan A. Waisel Y. (1980). Carbon Metabolism in Seagrasses II. Patterns of photosynthetic CO_2_ incorporation. *J. Exp. Bot.* 31 1019–1026. 10.1093/jxb/31.4.1019

[B5] Behboudian M. H. Törökfalvi E. Walker R. R. (1986). Effects of salinity on ionic content, water relations and gas exchange parameters in some *Citrus* scion-rootstock combinations. *Sci. Hort.* 28 105–116. 10.1016/0304-4238(86)90130-5

[B6] Bisson M. A. Kirst G. O. (1995). Osmotic acclimation and turgor pressure regulation in algae. *Naturwissenschaften* 82 461–471. 10.1007/BF01131597

[B7] Boudouresque F. C. Bernard G. Shili A. Verlaque M. (2009). Regression of Mediterranean seagrasses caused by natural processes and anthropogenic disturbances and stress: a critical review. *Bot. Mar.* 52 395–418. 10.1515/BOT.2009.057

[B8] Coors A. Kuckelkorn J. Hammers-Wirtz M. Strauss T. (2006). Application of in-situ bioassays with macrophytes in aquatic mesocosm studies. *Ecotoxicology* 15 583–591. 10.1007/s10646-006-0095-z 16960660

[B9] Dattolo E. Gu J. Bayer P. E. Mazzuca S. Serra I. A. Spadafora A. (2013). Acclimation to different depths by the marine angiosperm *Posidonia oceanica*: transcriptomic and proteomic profiles. *Front. Plant Sci.* 4:195 10.3389/fpls.2013.00195 PMC3683636 23785376

[B10] Dattolo E. Ruocco M. Brunet C. Lorenti M. Lauritano C. D’Esposito D. (2014). Response of the seagrass *Posidonia oceanica* to different light environments: insights from a combined molecular and photo-physiological study. *Mar. Environ. Res.* 101 225–236. 10.1016/j.marenvres.2014.07.010 25129449

[B11] Debez A. Saadaoui D. Ramani B. Ouerghi Z. Koyro H. W. Huchzermeyer B. (2006). Leaf H^+^-ATPase activity and photosynthetic capacity of *Cakilemaritima* under increasing salinity. *Environ. Exp. Bot.* 57 285–295. 10.1016/j.envexpbot.2005.06.009

[B12] Drapier D. Rimbault B. Vallon O. Wollmanand F. A. Choquet Y. (2007). Intertwined translational regulations set neve stoichiometry of chloroplast ATP synthase subunits. *EMBO J.* 26 3581–3591. 10.1038/sj.emboj.7601802 17660748 PMC1948998

[B13] Drew E. A. (1978). Factors affecting photosynthesis and its seasonal variation in the seagrass *Cymodocea nodosa* (Ucria) Aschers, and *Posidonia oceanica* (L.) Delile in the Mediterranean. *J. Exp. Mar. Biol. Ecol.* 31 173–194. 10.1016/0022-0981(78)90128-4

[B14] Elias J. E. Gygi S. P. (2007). Target-decoy search strategy for increased confidence in large-scale protein identifications by mass spectrometry. *Nat. Methods* 4 207–214. 10.1038/nmeth1019 17327847

[B15] Epimashko S. Fischer-Schliebs E. Christian A. L. Thiel G. Lüttge U. (2006). Na^+^/H^+^-transporter, H^+^-pumps and an aquaporin in light and heavy tonoplast membranes from organic acid and NaCl accumulating vacuoles of the annual facultative CAM plant and halophyte *Mesembryanthemum crystallinum* L. *Planta* 224 944–951. 10.1007/s00425-006-0265-5 16575596

[B16] Fenyö D. Eriksson J. Beavis R. (2010). “Mass spectrometric protein identification using the global proteome machine,” in *Computational Biology, Methodsin Molecular Biology* Vol. 673 ed. Fenyö D. (New York, NY: The Rockefeller University) 189–202. 10.1007/978-1-60761-842-3_11 PMC3757509 20835799

[B17] Fukuda A. Chiba K. Maeda M. Nakamura A. Maeshima M. Tanaka Y. (2004). Effect of salt and osmotic stresses on the expression of genes for the vacuolar H^+^-pyrophosphatase, H^+^-ATPase subunit A, and Na^+^/H^+^ antiporter from barley. *J. Exp. Bot. Mar.* 55 585–594. 10.1093/jxb/erh070 14754922

[B18] Garrote-Moreno A. Fernández-Torquemada Y. Sánchez-Lizaso J. L. (2014). Salinity fluctuation of the brine discharge affects growth and survival of the seagrass *Cymodocea nodosa*. *Mar. Pollut. Bull.* 81 61–68. 10.1016/j.marpolbul.2014.02.019 24635986

[B19] Kahn A. E. Durako M. J. (2006). Thalassia testudinum seedling responses to changes in salinity and nitrogen. *J. Exp. Mar. Biol. Ecol.* 335 1–12. 10.1016/j.jembe.2006.02.011

[B20] Kirst G. O. (1989). Salinity tolerance of eukaryotic marine algae. *Annu. Rev. Plant. Physiol. Mol. Biol.* 40 21–53.

[B21] Koch M. S. Schopmeyer S. A. Kyhn-Hansen C. Madden C. J. Peters J. S. (2007). Tropical seagrass species tolerance to hypersalinity stress. *Aquatic Bot.* 86 14–24. 10.1016/j.aquabot.2006.08.003

[B22] Kuo J. Den Hartog C. (2000). Seagrasses: a profile of an ecological group. *Biol. Mar. Mediterr.* 7 3–17.

[B23] Laemmli U. K. (1970). Cleavage of structural proteins during the assembly of the head of bacteriophage T4. *Nature* 227 680–685. 10.1038/227680a0 5432063

[B24] Marín-Guirao L. Ruiz J. M. Sandoval-Gil J. M. Bernardeau-Esteller J. Stinco C. M. Meléndez-Martínez A. J. (2013). Xanthophyll cycle-related photoprotective mechanism in the *Mediterranean seagrasses Posidonia oceanica* and *Cymodocea nodosa* under normal and stressful hypersaline conditions. *Aquatic Bot.* 109 14–24. 10.1016/j.aquabot.2013.03.006

[B25] Marín-Guirao L. Sandoval-Gil J. M. Ruiz J. M. Sánchez-Lizaso J. L. (2011). Photosynthesis, growth and survival of the *Mediterranean seagrass Posidonia oceanica* in response to simulated salinity increases in a laboratory mesocosm system. *Estuar. Coast. Shelf. S.* 92 286–296. 10.1016/j.ecss.2011.01.003

[B26] Mazzuca S. Björk M. Beer S. Felisberto P. Gobert S. Procaccini G. (2013). Establishing research strategies, methodologies and technologies to link genomics and proteomics to seagrass productivity, community metabolism, and ecosystem carbon fluxes. *Front. Plant Sci.* 4:38 10.3389/fpls.2013.00038 PMC3601598 23515425

[B27] Mazzuca S. Cozza R. Pangaro T. (2009). Tissue expression pattern of two aquaporin-encoding genes in different organs of the seagrass *Posidonia oceanica*. *Aquat. Bot.* 91 117–121. 10.1016/j.aquabot.2009.03.007

[B28] Montague C. L. Ley J. A. (1993). A possible effect of salinity fluctuation on abundance of benthic vegetation and associated fauna in Northeastern Florida Bay. *Estuaries* 16 703–717. 10.2307/1352429

[B29] Morais F. Barber J. Nixon P. J. (1998). The chloroplast encoded alpha subunit of cytochrome b is required for assembly of the photosystem two complex in both the light and the dark in *Chlamydomonas reinhardtii*. *J. Biol. Chem.* 273 29315–29320. 10.1074/jbc.273.45.29315 9792631

[B30] Müller B. Eichacker L. A. (1999). Assembly of the D1 precursor in monomeric photosystem II –reaction center precomplexes precedes chlorophyll a triggered accumulation of reaction center II in barley etioplasts. *Plant Cell* 11 2365–2378. 10.1105/tpc.11.12.2365 10590164 PMC144137

[B31] Muramatsu Y. Harada A. Ohwaki Y. Kasahara Y. Takagi S. Fukuhara T. (2002). Salt-tolerant ATPase activity in the plasma membraneof the marine angiosperm *Zostera marina* L. *Plant Cell Physiol.* 43 1137–1145. 10.1093/pcp/pcf139 12407193

[B32] Portillo E. Ruiz de la Rosa M. Louzara G. Ruiz J. M. Marín-Guirao L. Quesada J. (2014). Assessment of the abiotic and biotic effects of sodium metabisulphite pulses discharged from desalination plant chemical treatments on seagrass (*Cymodocea nodosa*) habitats in the Canary Islands. *Mar. Pollut. Bull.* 80 222–233. 10.1016/j.marpolbul.2013.12.048 24495930

[B33] Procaccini G. Buia M. C. Gambi M. C. Perez M. Pergent G. Pergent-Martini C. (2003). “Seagrasses of the western mediterranean,” in: *World Atlas of Seagrasses* eds Green E. P. Short F. T. (Berkeley, CA: University of California Press) 48–58.

[B34] Rott M. Martins N. F. Thiele W. Lein W. Bock R. Kramer D. M. (2011). ATP synthase repression in tobacco restricts photosynthetic electron transport, CO2 assimilation, and plant growth by over-acidification of the thylakoid lumen. *Plant Cell* 23 304–332. 10.1105/tpc.110.079111 21278125 PMC3051256

[B35] Ruiz J. M. Marín-Guirao L. Sandoval-Gil J. M. (2009). Responses of the *Mediterranean seagrass Posidonia oceanica* to in situ simulated salinity increase. *Bot. Mar.* 52 459–470. 10.1515/BOT.2009.051

[B36] Sánchez-Lizaso J. Romero J. Ruiz J. Gacia E. Buceta J. Invers O. (2008). Salinity tolerance of the *Mediterranean seagrass Posidonia oceanica*: recommendations to minimize the impact of brine discharges from desalination plants. *Desalination* 221 602–607. 10.1016/j.desal.2007.01.119

[B37] Sandoval-Gil J. M. Marín-Guirao L. Ruiz J. M. (2012). Tolerance of *Mediterranean seagrasses* (*Posidonia oceanica* and *Cymodocea nodosa*) to hypersaline stress: water relations and osmolyte concentrations. *Estuarine Coast. Shelf Sci.* 115 260–271. 10.1016/j.ecss.2012.09.008

[B38] Sandoval-Gil J. M. Ruiz J. M. Marín-Guirao L. Bernardeau-Esteller J. (2014). Ecophysiological plasticity of shallow and deep populations of the *Mediterranean seagrasses Posidonia oceanica* and *Cymodocea nodosa* in response to hypersaline stress. *J. Mar. Environ. Res.* 95 39–61. 10.1016/j.marenvres.2013.12.011 24411277

[B39] Schreiber U. (2004). “Pulse-Amplitude (PAM) fluorometry and saturation pulse method,” in: *Chlorophyll Fluorescence: A Signature of Photosynthesis* eds Papageorgiou G. Govindjee (Dordrecht: Springer) 279–319.

[B40] Serra I. A. Lauritano C. Dattolo E. Puoti A. Nicastro S. Innocenti A. M. (2012). Reference genes assessment for the seagrass *Posidonia oceanica* in different salinity, pH and light conditions. *Mar. Biol.* 159 1269–1282. 10.1007/s00227-012-1907-8

[B41] Serra I. A. Mazzuca S. (2011). “*Posidonia oceanica*: from ecological status to genetic and proteomic resources,” in *Seagrass: Ecology, Uses and Threats* ed. Pirog R. S. (New York: Nova Science Publishers, Inc.) 71–116.

[B42] Serra I. A. Nicastro S. Mazzuca S. Natali L. Cavallini A. Innocenti A. M. (2013). Response to salt stress in seagrasses: PIP1; 1 aquaporin antibody localization in *Posidonia oceanica* leaves. *Aquatic Bot.* 104 213–219. 10.1016/j.aquabot.2011.05.008

[B43] Spadafora A. Filadoro D. Mazzuca S. Bracale M. Marsoni M. Cardilio M. (2008). 2-DE polypeptide mapping of *Posidonia oceanica* leaves, a molecular tool for marine environment studies. *Plant Biosyst.* 142 213–218. 10.1080/11263500802150316

[B44] Srivastava A. K. Ramaswamy N. K. Mukopadhyaya R. Chiramal Jincy M. G. D’Souza S. F. (2009). Thiourea modulates the expression and activity profile of mtATPase under salinity stress in seeds of *Brassica juncea*. *Ann. Bot.* 103 403–410. 10.1093/aob/mcn229 19033283 PMC2707324

[B45] Taji T. Seki M. Satou M. Sakurai T. Kobayashi M. Ishiyama K. (2004). Comparative genomics in salt tolerance between *Arabidopsis* and *Arabidopsis-*related halophyte salt cress using *Arabidopsis* microarray. *Plant Physiol.* 135 1697–1709. 10.1104/pp.104.039909 15247402 PMC519083

[B46] Touchette B. W. (2007). Seagrass-salinity interactions: physiological mechanisms used by submersed marine angiosperms for a life at sea. *J. Exp. Mar. Biol. Ecol.* 350 194–215. 10.1016/j.jembe.2007.05.037

[B47] Tyerman S. D. (1989). “Solute and water relations of seagrasses,” in: *Biology of Seagrasses. A Treatise on the Biology of Seagrasses with Special Reference to the Australian region* eds Larkum A. W. D. McComb A. J. Shepherd S. A. (Amsterdam: Elsevier) 723–759.

[B48] van Wijk K. J. Roobol-Boza M. Kettunen R. Andersson B. Aro E. M. (1997). Synthesis and assembly of the D1 protein into photosystem II: processing of the C-terminus and identification of the initial assembly partners and complexes during photosystem II repair. *Biochemistry* 36 6178–6186. 10.1021/bi962921l 9166790

[B49] Wang L. Pana D. Lia J. Tane F. Hoffmann-Benningc S. Liangd W. (2015). Proteomic analysis of changes in the *Kandelia candel* chloroplastproteins reveals pathways associated with salt tolerance. *Plant Sci.* 231 159–172. 10.1016/j.plantsci.2014.11.013 25576001

[B50] Wang W. Scali M. Cresti M. (2006). A universal and rapid protocol for protein extraction from recalcitrant plant tissues for proteomic analysis. *Electrophoresis* 27 2782–2786. 10.1002/elps.200500722 16732618

[B51] Wilm M. Shevchenko A. Houthaeve T. Breit S. Schweigerer L. Fotsis T. (1996). Fentomole sequencing of proteins from polyacrylamide gels by nano-electrospray mass spectrometry. *Nature* 379 466–469. 10.1038/379466a0 8559255

[B52] Wissler L. Dattolo E. Moore A. D. Reusch T. B. H. Olsen J. L. Migliaccio M. (2009). Dr. zompo: an online data repository for *zostera marina* and *Posidonia oceanica* ESTs. *Database (Oxford)* 2009:bap009 10.1093/database/bap009 PMC2790305 20157482

[B53] Zhang L. Aro E. M. (2002). Synthesis, membrane insertion and assembly of the chloroplast-encoded D1 protein into photosystem II. *FEBS Lett.* 512 13–18. 10.1016/S0014-5793(02)02218-4 11852043

[B54] Zhang Y. Wen Z. Washburn M. P. Florens L. (2010). Refinements to label free proteome quantitation: how to deal with peptides shared by multiple proteins. *Anal. Chem.* 82 2272–2281. 10.1021/ac9023999 20166708

[B55] Zimmerman U. (1978). Physics of turgor and osmoregulation. *Annu. Rev. Plant. Physiol.* 29 121–148. 10.1146/annurev.pp.29.060178.001005

[B56] Zybailov B. Mosley A. L. Sardiu M. E. Coleman M. K. Florens L. Washburn M. P. J. (2006). Statistical analysis of membrane proteome expression changes in *Saccharomyces cerevisiae*. *Proteome Res.* 5 2339–2347. 10.1021/pr060161n 16944946

