# Supplementary material for: The modulation of leaf metabolism plays a role in salt tolerance of Cymodocea nodosa exposed to hypersaline stress in mesocosms
Source: Front Plant Sci. 2015 Jun 26;6:464. doi: 10.3389/fpls.2015.00464 (PMC4482034; doi:10.3389/fpls.2015.00464)
Supplement: Supplementary file 1 [file Data_Sheet_1.DOCX]

**Supplementary Table 1**: List of proteins identified in control and hypersaline-treated plants of *Cymodocea nodosa* at 15 and 30 days treatment in the mesocosm. Statistical parameters, number of unique peptides found for each protein, spectra number assigned to each protein, protein molecular weight, percentage of spectra assigned to each identified protein. NCBI description and accession ID obtained through BLAST search (blastp) of the recognized proteins are also shown.

| Control sample 1 | | | | | | | | | | | | | | | |  |
| --- | --- | --- | --- | --- | --- | --- | --- | --- | --- | --- | --- | --- | --- | --- | --- | --- |
| rank | log (e) | log (I) | % (m) | % (c) | | | unique | | total | | Mr | | Accession | Description | | % spectra |
| 1 | -8 | 6,9 | 4,2 | 9 | | | 2 | | 3 | | 50,4 | | tr\|Q6L9Z6\|Q6L9Z6_9LILI | RuBisCO large subunit; | | 2,88 |
| 2 | -3,9 | 5,6 | 2,1 | 3 | | | 1 | | 1 | | 74 | | tr\|Q2QV45\|Q2QV45_ORYSJ | 70 kDa heat shock protein; Os12g0244100 protein; | | 0,96 |
| 3 | -3,5 | 6,2 | 4,3 | 5 | | | 1 | | 3 | | 33,2 | | sp\|P08477\|G3PC_HORVU | Glyceraldehyde-3-phosphate dehydrogenase, cytosolic; | | 2,88 |
| 4 | -1,8 | 5,9 | 1,6 | 2 | | | 1 | | 1 | | 72,9 | | sp\|Q7SIC9\|TKTC_MAIZE | Transketolase:p , chloroplastic; TK; EC 2.2.1.1; | | 0,96 |
| 5 | -1,7 | 5,6 | 1,5 | 2 | | | 1 | | 1 | | 111,4 | | tr\|Q6V9T1\|Q6V9T1_ORYSJ | Glycine dehydrogenase P protein; Os01g0711400 protein; | | 0,96 |
| 1 | -54 | 7,4 | 17 | 21 | | | 6 | | 13 | | 53,6 | | tr\|H2CPP4\|H2CPP4_COLES | EC 3.6.3.14; ATP synthase F1 sector subunit beta; | | 12,5 |
| 2 | -44 | 7,3 | 3,2 | 3 | | | 1 | | 1 | | 59,1 | | tr\|Q4FGI4\|Q4FGI4_TYPLA | ATP synthase subunit beta; EC 3.6.3.14; | | 0,96 |
| 3 | -42 | 7,3 | 2 | 2 | | | 1 | | 2 | | 55,3 | | sp\|Q95AD6\|ATPB_WHIBI | EC 3.6.3.14; ATP synthase F1 sector subunit beta; | | 1,92 |
| 4 | -38 | 7,2 | 12 | 21 | | | 5 | | 8 | | 55,3 | | sp\|A9LYH0\|ATPA_ACOAM | EC 3.6.3.14; ATP synthase F1 sector subunit alpha; | | 7,69 |
| 5 | -36 | 7,4 | 11 | 24 | | | 5 | | 8 | | 50,4 | | tr\|Q6L9Z6\|Q6L9Z6_9LILI | RuBisCO large subunit; | | 7,69 |
| 6 | -28 | 7,1 | 2 | 2 | | | 1 | | 1 | | 55,3 | | sp\|P62626\|ATPB_AEGCO | EC 3.6.3.14; ATP synthase F1 sector subunit beta; | | 0,96 |
| 7 | -19 | 6,9 | 2,3 | 2 | | | 1 | | 1 | | 50,4 | | tr\|C6G4V9\|C6G4V9_9ASPA | Ribulose-1:p,5-bisphosphate carboxylase/oxygenase large subunit; | | 0,96 |
| 8 | -19 | 6,2 | 6,3 | 8 | | | 3 | | 3 | | 59,1 | | sp\|P19023\|ATPBM_MAIZE | ATP synthase subunit beta, mitochondrial; EC 3.6.3.14; | | 2,88 |
| 9 | -9,3 | 5,9 | 4,6 | 6 | | | 2 | | 2 | | 61,4 | | tr\|Q7X9A7\|Q7X9A7_ORYSJ | 60 kDa chaperonin alpha subunit; Putative rubisco subunit binding-protein alpha subunit; | | 1,92 |
| 10 | -5,1 | 5,2 | 4,1 | 6 | | | 1 | | 1 | | 55,1 | | sp\|P05494\|ATPAM_MAIZE | ATP synthase subunit alpha, mitochondrial; | | 0,96 |
| 1 | -22 | 6,8 | 10 | 15 | | | 3 | | 5 | | 42,7 | | tr\|F2D714\|F2D714_HORVD | Predicted protein; | | 4,8 |
| 2 | -22 | 6,7 | 7,9 | 9 | | | 3 | | 4 | | 50 | | tr\|Q1ENY9\|Q1ENY9_MUSAC | Phosphoglycerate kinase, chloroplast, putative; EC 2.7.2.3; | | 3,8 |
| 3 | -21 | 6,6 | 3,5 | 4 | | | 1 | | 1 | | 0 | | sp\|P12782\|PGKH_WHEAT | no protein information available | | 0,96 |
| 4 | -20 | 6,7 | 1,9 | 2 | | | 1 | | 1 | | 50 | | tr\|B6STH5\|B6STH5_MAIZE | Phosphoglycerate kinase; EC 2.7.2.3; | | 0,96 |
| 5 | -18 | 6,3 | 7,8 | 10 | | | 3 | | 3 | | 53,6 | | sp\|Q3V527\|ATPB_ACOCL | EC 3.6.3.14; ATP synthase F1 sector subunit beta; | | 2,88 |
| 6 | -11 | 6,3 | 5,4 | 6 | | | 1 | | 1 | | 31,4 | | tr\|C1JYE2\|C1JYE2_9POAL | Phosphoglycerate kinase; EC 2.7.2.3; | | 0,96 |
| 7 | -7,4 | 6,1 | 5 | 12 | | | 2 | | 2 | | 48,9 | | tr\|O78641\|O78641_9ASPA | no protein information available | | 1,92 |
| 8 | -4,7 | 5,7 | 4,2 | 6 | | | 1 | | 1 | | 47,2 | | sp\|Q42450\|RCAB_HORVU | no protein information available | | 0,96 |
| 9 | -3,7 | 6,4 | 3,3 | 5 | | | 1 | | 2 | | 39,8 | | sp\|P0C1M0\|ATPG_MAIZE | ATP synthase subunit gamma, chloroplastic; F-ATPase gamma sub. | | 1,92 |
| 10 | -3,5 | 6,1 | 5,1 | 8 | | | 1 | | 1 | | 33 | | sp\|P27337\|PER1_HORVU | Peroxidase 1; EC 1.11.1.7; | | 0,96 |
| 11 | -3,4 | 5,7 | 2,9 | 4 | | | 1 | | 1 | | 47,1 | | tr\|Q9SNK3\|Q9SNK3_ORYSJ | Glyceraldehyde-3-phosphate dehydrogenase B, chloroplast | | 0,96 |
| 12 | -3,1 | 5,4 | 3,4 | 5 | | | 1 | | 1 | | 42 | | sp\|Q40677\|ALFC_ORYSJ | Fructose-bisphosphate aldolase, chloroplastic; ALDP; EC 4.1.2.13; | | 0,96 |
| 13 | -2,7 | 5,7 | 4,7 | 6 | | | 1 | | 1 | | 35,2 | | sp\|A6MMM0\|CYF_DIOEL | Apocytochrome f; | | 0,96 |
| 15 | -2,1 | 5,8 | 3,2 | 4 | | | 1 | | 1 | | 44,5 | | sp\|P37833\|AATC_ORYSJ | Aspartate aminotransferase, cytoplasmic; EC 2.6.1.1; Transaminase A; | | 0,96 |
| 19 | -1,3 | 6,7 | 3,3 | 5 | | | 1 | | 3 | | 35,6 | | sp\|Q08062\|MDHC_MAIZE | Malate dehydrogenase, cytoplasmic; EC 1.1.1.37; | | 2,88 |
| 1 | -11 | 6,2 | 6,1 | 9 | | | 2 | | 2 | | 41 | | tr\|Q0DJC0\|Q0DJC0_ORYSJ | Os05g0302700 protein; cDNA clone:001-036-B04, full insert sequence; | | 1,92 |
| 2 | -8,2 | 6,2 | 5 | 12 | | | 2 | | 2 | | 49,2 | | tr\|Q8WL39\|Q8WL39_9ASPA | Ribulose-bisphosphate carboxylase large subunit; | | 1,92 |
| 3 | -2,5 | 5,5 | 3 | 3 | | | 1 | | 1 | | 42,3 | | tr\|Q1EPF8\|Q1EPF8_MUSAC | Phosphoglycerate kinase 2; EC 2.7.2.3; | | 0,98 |
| 4 | -2,5 | 5,7 | 3,9 | 6 | | | 1 | | 1 | | 29,6 | | sp\|Q6L5I5\|VDAC2_ORYSJ | Mitochondrial outer membrane protein porin 2; OsVDAC2; | | 0,98 |
| 5 | -2,4 | 5,4 | 2 | 2 | | | 1 | | 1 | | 63,8 | | tr\|Q6ZFJ9\|Q6ZFJ9_ORYSJ | 60 kDa chaperonin beta subunit; Os02g0102900 protein; | | 0,98 |
| 6 | -2 | 6,1 | 4,9 | 6 | | | 1 | | 2 | | 34,4 | | tr\|F2CRK1\|F2CRK1_HORVD | Predicted protein; | | 1,92 |
| 9 | -1,5 | 5,8 | 5,7 | 8 | | | 1 | | 2 | | 29,8 | | tr\|G0YLW6\|G0YLW6_9ARAE | Putative chlorophyll a/b binding protein; | | 1,92 |
| 1 | -23 | 6,7 | 15 | 25 | | | 3 | | 10 | | 27,7 | | tr\|Q6WFB1\|Q6WFB1_MAIZE | Photosystem II subunit PsbS | | 9,6 |
| 2 | -4 | 5,5 | 6,8 | 12 | | | 1 | | 1 | | 21,9 | | sp\|P36213\|PSAD_HORVU | Photosystem I reaction center subunit II, chloroplastic; Photosystem I 20 kDa subunit; | | 0,96 |
| 7 | -1,4 | 5,7 | 4,3 | 5 | | | 1 | | 1 | | 33,2 | | sp\|P08477\|G3PC_HORVU | Glyceraldehyde-3-phosphate dehydrogenase, cytosolic; EC 1.2.1.12; | | 0,96 |
| 1 | -1,9 | 5,7 | 13 | 14 | | | 1 | | 1 | | 9,3 | | tr\|I1IWU7\|I1IWU7_BRADI | Uncharacterized protein; | | 0,96 |
| 2 | -1,8 | 5,8 | 13 | 28 | | | 1 | | 1 | | 9,4 | | sp\|A1EA25\|PSBE_AGRST | no protein information available | | 0,96 |
|  |  |  |  |  | | |  | |  | |  | |  |  | |  |
| Hypersaline sample 1 (15 days) | | | | | | | | | | | | | | | |  |
| rank | log(e) | log(I) | % (m) | % (c) | | unique | | total | | Mr | | Accession | | | Description |  |
| 1 | -37 | 6,8 | 11 | 24 | | 5 | | 8 | | 50,4 | | tr\|Q6L9Z6\|Q6L9Z6_9LILI | | | RuBisCO large subunit; | 7,69 |
| 2 | -3,3 | 5,2 | 2,6 | 7 | | 1 | | 1 | | 56 | | sp\|A9LYC6\|PSBB_ACOAM | | | Photosystem II CP47 chlorophyll apoprotein; | 0,96 |
| 3 | -2,3 | 5,4 | 2 | 3 | | 1 | | 1 | | 71,5 | | tr\|C5YWM8\|C5YWM8_SORBI | | | no protein information available | 0,96 |
| 4 | -2,2 | 5,7 | 4,3 | 5 | | 1 | | 1 | | 33,2 | | sp\|P08477\|G3PC_HORVU | | | Glyceraldehyde-3-phosphate dehydrogenase, cytosolic; EC 1.2.1.12; | 0,96 |
| 5 | -1,8 | 5,7 | 3 | 5 | | 1 | | 1 | | 55,3 | | sp\|A9LYH0\|ATPA_ACOAM | | | EC 3.6.3.14; ATP synthase F1 sector subunit alpha; F-ATPase subunit alpha; | 0,96 |
| 1 | -46 | 6,8 | 14 | 17 | | 5 | | 9 | | 53,6 | | tr\|H2CPP4\|H2CPP4_COLES | | | EC 3.6.3.14; ATP synthase F1 sector subunit beta; F-ATPase subunit beta; | 8,65 |
| 2 | -43 | 6,8 | 5 | 5 | | 1 | | 1 | | 59,1 | | tr\|H6THB0\|H6THB0_9LILI | | | ATP synthase subunit beta; EC 3.6.3.14; | 0,98 |
| 3 | -38 | 6,8 | 7 | 16 | | 5 | | 9 | | 49 | | tr\|B5RHG8\|B5RHG8_9ASPA | | | Ribulose-bisphosphate carboxylase large subunit; | 0,86 |
| 4 | -32 | 6,8 | 9,7 | 18 | | 4 | | 6 | | 55,3 | | sp\|A9LYH0\|ATPA_ACOAM | | | EC 3.6.3.14; ATP synthase F1 sector subunit alpha; F-ATPase subunit alpha; | 5,76 |
| 5 | -29 | 6,7 | 4,7 | 11 | | 2 | | 2 | | 51,6 | | tr\|B0B735\|B0B735_9POAL | | | Ribulose-1:p,5-bisphosphate carboxylase/oxygenase large subunit; EC 4.1.1.39; | 1,92 |
| 6 | -29 | 6,6 | 2,2 | 2 | | 1 | | 1 | | 55,1 | | tr\|F8RS97\|F8RS97_JUNEF | | | ATP synthase subunit alpha | 0,96 |
| 7 | -29 | 7,1 | 2,2 | 2 | | 1 | | 3 | | 50,4 | | tr\|Q6L9Z6\|Q6L9Z6_9LILI | | | RuBisCO large subunit; | 2,88 |
| 8 | -20 | 6,7 | 2 | 2 | | 1 | | 1 | | 55,3 | | sp\|P62626\|ATPB_AEGCO | | | EC 3.6.3.14; ATP synthase F1 sector subunit beta; | 0,96 |
| 9 | -18 | 6,2 | 7,4 | 9 | | 3 | | 4 | | 59,1 | | sp\|P19023\|ATPBM_MAIZE | | | ATP synthase subunit beta, mitochondrial; EC 3.6.3.14; | 3,84 |
| 10 | -6,1 | 5,9 | 2 | 2 | | 1 | | 2 | | 63,8 | | tr\|Q6ZFJ9\|Q6ZFJ9_ORYSJ | | | 60 kDa chaperonin beta subunit; Os02g0102900 protein; | 1,92 |
| 1 | -22 | 6,5 | 7,9 | 9 | | 3 | | 3 | | 50 | | tr\|Q1ENY9\|Q1ENY9_MUSAC | | | Phosphoglycerate kinase, chloroplast, putative; EC 2.7.2.3; | 2,88 |
| 2 | -20 | 6,4 | 3,3 | 3 | | 1 | | 1 | | 50 | | tr\|B6STH5\|B6STH5_MAIZE | | | Phosphoglycerate kinase; EC 2.7.2.3; | 0,96 |
| 3 | -9,8 | 6,6 | 14 | 19 | | 2 | | 3 | | 20,8 | | tr\|F8UCA0\|F8UCA0_9LILI | | | Glyceraldehyde-3-phosphate dehydrogenase; EC 1.2.1.12; | 2,88 |
| 4 | -9 | 6,5 | 6,7 | 10 | | 2 | | 2 | | 42,7 | | tr\|F2D714\|F2D714_HORVD | | | Predicted protein; | 1,92 |
| 5 | -2,8 | 5,4 | 5,3 | 6 | | 1 | | 1 | | 31,5 | | tr\|G3FBL3\|G3FBL3_9LILI | | | Actin; Flags: Fragment | 0,96 |
| 7 | -1,6 | 5,6 | 5,1 | 6 | | 1 | | 1 | | 37 | | tr\|Q7XZW5\|Q7XZW5_ORYSJ | | | Malate dehydrogenase; EC 1.1.1.37 | 0,96 |
| 12 | -1,2 | 5,8 | 3,3 | 5 | | 1 | | 1 | | 39,8 | | sp\|P0C1M0\|ATPG_MAIZE | | | ATP synthase subunit gamma, chloroplastic; F-ATPase gamma subunit; | 0,96 |
| 1 | -3,5 | 5,5 | 4,3 | 6 | | 1 | | 1 | | 26,7 | | sp\|P34937\|TPIS_HORVU | | | Triosephosphate isomerase, cytosolic; TIM; Triose-phosphate isomerase; EC 5.3.1.1 | 0,96 |
| 2 | -2,4 | 5,8 | 4,9 | 6 | | 1 | | 2 | | 34,4 | | tr\|F2CRK1\|F2CRK1_HORVD | | | Predicted protein; | 1,92 |
| 3 | -1,9 | 5,2 | 5,2 | 6 | | 1 | | 1 | | 26 | | tr\|Q6YTY2\|Q6YTY2_ORYSJ | | | Os07g0608500 protein; Putative 40S ribosomal protein; | 0,96 |
| 4 | -1,6 | 5,6 | 2,8 | 4 | | 1 | | 1 | | 42,4 | | sp\|P04709\|ADT1_MAIZE | | | ADP:p ,ATP carrier protein 1, mitochondrial; ADP/ATP translocase 1; | 0,96 |
| 1 | -3,8 | 5,8 | 3,8 | 6 | | 1 | | 1 | | 24,8 | | sp\|P13192\|PSAF_HORVU | | | Light-harvesting complex I 17 kDa protein; | 0,96 |
| 1 | -2,3 | 5,7 | 13 | 28 | | 1 | | 2 | | 9,4 | | sp\|A1EA25\|PSBE_AGRST | | | Cytochrome b559 subunit alpha; PSII reaction center subunit V; | 1,92 |
|  |  |  |  |  | |  | |  | |  | |  | | |  |  |
| Hypersaline sample 1 (30 days) | | | | | | | | | | | | | | | |  |
| rank | log(e) | log(I) | % (m) | % (c) | | unique | | total | | Mr | | Accession | | | Description |  |
| 1 | -18 | 6,3 | 8,2 | 12 | | 3 | | 3 | | 41,7 | | tr\|C7IWD0\|C7IWD0_ORYSJ | | | Os01g0791600 protein; | 4,54 |
| 2 | -9,3 | 6,2 | 4,3 | 8 | | 2 | | 3 | | 55,3 | | sp\|A9LYH0\|ATPA_ACOAM | | | EC 3.6.3.14; ATP synthase F1 sector subunit alpha; F-ATPase subunit alpha; | 4,54 |
| 3 | -7,7 | 6,4 | 2,2 | 2 | | 1 | | 2 | | 50,4 | | tr\|Q6L9Z6\|Q6L9Z6_9LILI | | | RuBisCO large subunit; | 3,03 |
| 4 | -4,3 | 5,4 | 3,8 | 4 | | 1 | | 1 | | 48,1 | | sp\|P42895\|ENO2_MAIZE | | | no protein information available | 1,52 |
| 6 | -2,4 | 5,5 | 1,5 | 2 | | 1 | | 1 | | 104,8 | | sp\|Q7XPY2\|PMA1_ORYSJ | | | no protein information available | 1,52 |
| 7 | -2,2 | 5,5 | 3,5 | 11 | | 1 | | 1 | | 50,8 | | tr\|G1C6J9\|G1C6J9_9LILI | | | no protein information available | 1,52 |
| 10 | -1,8 | 5,5 | 1,3 | 2 | | 5 | | 5 | | 79,5 | | sp\|Q06572\|AVP_HORVU | | | EC 3.6.1.1; Pyrophosphate-energized inorganic pyrophosphatase; | 7,57 |
| 1 | -54 | 6,9 | 18 | 22 | | 6 | | 10 | | 53,6 | | sp\|A9L9A3\|ATPB_LEMMI | | | EC 3.6.3.14; ATP synthase F1 sector subunit beta; F-ATPase subunit beta; | 14,3 |
| 2 | -52 | 6,9 | 3,2 | 3 | | 1 | | 1 | | 53,6 | | tr\|H2CPP4\|H2CPP4_COLES | | | EC 3.6.3.14; ATP synthase F1 sector subunit beta; F-ATPase subunit beta; | 1,52 |
| 3 | -32 | 6,7 | 9,7 | 18 | | 4 | | 5 | | 55,3 | | sp\|A9LYH0\|ATPA_ACOAM | | | EC 3.6.3.14; ATP synthase F1 sector subunit alpha; F-ATPase subunit alpha; | 7,57 |
| 4 | -12 | 5,8 | 7 | 8 | | 2 | | 2 | | 48,1 | | sp\|P42895\|ENO2_MAIZE | | | Enolase 2; EC 4.2.1.11; 2-phospho-D-glycerate hydro-lyase 2; | 3,03 |
| 5 | -10 | 6,3 | 4,7 | 6 | | 2 | | 3 | | 59,1 | | sp\|P19023\|ATPBM_MAIZE | | | ATP synthase subunit beta, mitochondrial; EC 3.6.3.14; | 4,54 |
| 6 | -9,5 | 6,6 | 4,4 | 10 | | 2 | | 3 | | 50,4 | | tr\|Q6L9Z6\|Q6L9Z6_9LILI | | | RuBisCO large subunit; | 4,54 |
| 7 | -3,5 | 5,7 | 2 | 2 | | 1 | | 2 | | 63,8 | | tr\|Q6ZFJ9\|Q6ZFJ9_ORYSJ | | | 60 kDa chaperonin beta subunit; Os02g0102900 protein; | 3,03 |
| 12 | -1,5 | 5,3 | 2,1 | 3 | | 1 | | 1 | | 61,4 | | tr\|Q7X9A7\|Q7X9A7_ORYSJ | | | 60 kDa chaperonin alpha subunit; | 1,52 |
| 1 | -23 | 6,3 | 8,8 | 11 | | 3 | | 4 | | 49,8 | | tr\|B6STH5\|B6STH5_MAIZE | | | Phosphoglycerate kinase; EC 2.7.2.3; | 6,06 |
| 2 | -21 | 6,3 | 2,5 | 3 | | 1 | | 1 | | 50 | | tr\|Q1ENY9\|Q1ENY9_MUSAC | | | Phosphoglycerate kinase, chloroplast, putative; EC 2.7.2.3; | 1,52 |
| 3 | -11 | 6,1 | 4 | 4 | | 1 | | 1 | | 0 | | tr\|Q655T1\|Q655T1_ORYSJ | | | no protein information available | 1,52 |
| 4 | -11 | 6,7 | 14 | 19 | | 2 | | 3 | | 20,8 | | tr\|F8UCA0\|F8UCA0_9LILI | | | Glyceraldehyde-3-phosphate dehydrogenase; EC 1.2.1.12; | 4,54 |
| 5 | -9,9 | 6,7 | 3,9 | 4 | | 1 | | 1 | | 20,1 | | tr\|Q7FAH2\|Q7FAH2_ORYSJ | | | Glyceraldehyde-3-phosphate dehydrogenase 2, cytosolic; EC 1.2.1.12 | 1,52 |
| 6 | -2,9 | 6,1 | 5,1 | 8 | | 1 | | 1 | | 33 | | sp\|P27337\|PER1_HORVU | | | Peroxidase 1; EC 1.11.1.7; | 1,52 |
| 7 | -2,1 | 5,4 | 3,2 | 5 | | 1 | | 1 | | 42,7 | | tr\|F2D714\|F2D714_HORVD | | | Predicted protein; | 1,52 |
| 11 | -1,5 | 5,9 | 3,3 | 8 | | 1 | | 2 | | 50,1 | | sp\|P25776\|ORYA_ORYSJ | | | no protein information available | 3,3 |
| 1 | -9,4 | 6,2 | 9,8 | 12 | | 2 | | 4 | | 34,4 | | tr\|F2CRK1\|F2CRK1_HORVD | | | Predicted protein; | 6,06 |
| 2 | -4,9 | 5,9 | 3,2 | 5 | | 1 | | 1 | | 41 | | tr\|Q0DJC0\|Q0DJC0_ORYSJ | | | Os05g0302700 protein; | 1,52 |
| 3 | -4,6 | 5,6 | 8,5 | 12 | | 1 | | 1 | | 24,7 | | tr\|F2DTJ2\|F2DTJ2_HORVD | | | Predicted protein | 1,52 |
| 4 | -3,4 | 5,8 | 5,1 | 8 | | 1 | | 1 | | 33 | | sp\|P27337\|PER1_HORVU | | | Peroxidase 1; EC 1.11.1.7; | 1,52 |
| 7 | -2 | 5,7 | 5,4 | 7 | | 1 | | 1 | | 29,8 | | tr\|G0YLW6\|G0YLW6_9ARAE | | | Putative chlorophyll a/b binding protein; | 1,52 |
| 1 | -2,7 | 6,2 | 3,8 | 6 | | 1 | | 2 | | 24,8 | | sp\|P13192\|PSAF_HORVU | | | Light-harvesting complex I 17 kDa protein; PSI-F; | 3,3 |
| 2 | -2,4 | 5,4 | 5,7 | 9 | | 1 | | 1 | | 27,7 | | tr\|Q6WFB1\|Q6WFB1_MAIZE | | | Photosystem II subunit PsbS; | 1,52 |
| 1 | -1,5 | 5,5 | 13 | 28 | | 1 | | 2 | | 9,4 | | sp\|A1EA25\|PSBE_AGRST | | | Cytochrome b559 subunit alpha; PSII reaction center subunit V; | 3,3 |
|  | | | | | | | | | | | | | | | |  |
|  | | | | | | | | | | | | | | | |  |
|  | | | | | | | | | | | | | | | |  |
|  | | | | | | | | | | | | | | | |  |
|  | | | | | | | | | | | | | | | |  |
|  | | | | | | | | | | | | | | | |  |
|  | | | | | | | | | | | | | | | |  |
|  | | | | |  | | | | | | | | | | |  |

| Control sample 2 | | | | | | | | | | | | | | | | |  |
| --- | --- | --- | --- | --- | --- | --- | --- | --- | --- | --- | --- | --- | --- | --- | --- | --- | --- |
| **rank** | **log (e)** | **log (I)** | **% (m)** | **% (cc)** | | **unique** | | **total** | | | **Mr** | | | **Accession** | | **Description** | **% of spectra** |
| 1 | -9 | 8,9 | 4,3 | 9 | | 4 | | 4 | | | 50,4 | | | tr\|Q6L9Z6\|Q6L9Z6_9LILI | | RuBisCO large subunit; | 3,57 |
| 2 | -3,7 | 6,5 | 2,3 | 4 | | 3 | | 3 | | | 74 | | | tr\|Q2QV45\|Q2QV45_ORYSJ | | 70 kDa heat shock protein; Os12g0244100 protein; | 2,67 |
| 3 | -4,1 | 7,2 | 3,3 | 6 | | 1 | | 3 | | | 33,2 | | | sp\|P08477\|G3PC_HORVU | | Glyceraldehyde-3-phosphate dehydrogenase, cytosolic; | 2,67 |
| 4 | -2,8 | 6,5 | 2,1 | 3 | | 2 | | 2 | | | 72,9 | | | sp\|Q7SIC9\|TKTC_MAIZE | | Transketolase:p , chloroplastic; TK; EC 2.2.1.1; | 1,78 |
| 5 | -1,2 | 6,6 | 1,3 | 2 | | 1 | | 1 | | | 111,4 | | | tr\|Q6V9T1\|Q6V9T1_ORYSJ | | Glycine dehydrogenase P protein; Os01g0711400 protein; | 0,89 |
| 1 | -63 | 6,2 | 15 | 20 | | 7 | | 15 | | | 53,6 | | | tr\|H2CPP4\|H2CPP4_COLES | | EC 3.6.3.14; ATP synthase F1 sector subunit beta; | 13,39 |
| 2 | -35 | 7,8 | 4,2 | 4 | | 1 | | 1 | | | 59,1 | | | tr\|Q4FGI4\|Q4FGI4_TYPLA | | ATP synthase subunit beta; EC 3.6.3.14; | 0,89 |
| 3 | -38 | 7,0 | 2 | 2 | | 1 | | 2 | | | 55,3 | | | sp\|Q95AD6\|ATPB_WHIBI | | EC 3.6.3.14; ATP synthase F1 sector subunit beta; | 1,78 |
| 4 | -38 | 6,8 | 13 | 22 | | 5 | | 8 | | | 55,3 | | | sp\|A9LYH0\|ATPA_ACOAM | | EC 3.6.3.14; ATP synthase F1 sector subunit alpha; | 7,14 |
| 5 | -29 | 8,0 | 12 | 26 | | 6 | | 9 | | | 50,4 | | | tr\|Q6L9Z6\|Q6L9Z6_9LILI | | RuBisCO large subunit; | 8,03 |
| 6 | -27 | 8,1 | 2 | 2 | | 1 | | 1 | | | 55,3 | | | sp\|P62626\|ATPB_AEGCO | | EC 3.6.3.14; ATP synthase F1 sector subunit beta; | 0,89 |
| 7 | -17 | 7,3 | 2,5 | 2 | | 1 | | 1 | | | 50,4 | | | tr\|C6G4V9\|C6G4V9_9ASPA | | Ribulose-1:p,5-bisphosphate carboxylase/oxygenase large subunit; | 0,89 |
| 8 | -17 | 6,5 | 5,8 | 8 | | 3 | | 3 | | | 59,1 | | | sp\|P19023\|ATPBM_MAIZE | | ATP synthase subunit beta, mitochondrial; EC 3.6.3.14; | 2,67 |
| 9 | -9,5 | 6,2 | 4,1 | 6 | | 2 | | 2 | | | 61,4 | | | tr\|Q7X9A7\|Q7X9A7_ORYSJ | | 60 kDa chaperonin alpha subunit; Putative rubisco subunit binding-protein alpha subunit; | 1,78 |
| 10 | -5,21 | 5,5 | 4,3 | 6 | | 1 | | 1 | | | 55,1 | | | sp\|P05494\|ATPAM_MAIZE | | ATP synthase subunit alpha, mitochondrial; | 0,89 |
| 1 | -23 | 6,5 | 12 | 15 | | 3 | | 5 | | | 42,7 | | | tr\|F2D714\|F2D714_HORVD | | Predicted protein; | 4,46 |
| 2 | -22 | 6,7 | 7,9 | 9 | | 3 | | 4 | | | 50 | | | tr\|Q1ENY9\|Q1ENY9_MUSAC | | Phosphoglycerate kinase, chloroplast, putative; EC 2.7.2.3; | 3,57 |
| 3 | -24 | 6,9 | 3,9 | 4 | | 1 | | 1 | | | 0 | | | sp\|P12782\|PGKH_WHEAT | | no protein information available | 0,89 |
| 4 | -22 | 6,8 | 2,3 | 2 | | 1 | | 1 | | | 50 | | | tr\|B6STH5\|B6STH5_MAIZE | | Phosphoglycerate kinase; EC 2.7.2.3; | 0,89 |
| 5 | -19 | 6,5 | 7,9 | 11 | | 3 | | 3 | | | 53,6 | | | sp\|Q3V527\|ATPB_ACOCL | | EC 3.6.3.14; ATP synthase F1 sector subunit beta; | 2,67 |
| 6 | -11 | 6,3 | 5,4 | 6 | | 1 | | 1 | | | 31,4 | | | tr\|C1JYE2\|C1JYE2_9POAL | | Phosphoglycerate kinase; EC 2.7.2.3; | 0,89 |
| 7 | -7,4 | 6,1 | 5 | 12 | | 2 | | 2 | | | 48,9 | | | tr\|O78641\|O78641_9ASPA | | no protein information available | 1,75 |
| 8 | -4,7 | 5,7 | 4,2 | 6 | | 1 | | 1 | | | 47,2 | | | sp\|Q42450\|RCAB_HORVU | | no protein information available | 0,89 |
| 9 | -3,7 | 6,4 | 3,3 | 5 | | 1 | | 2 | | | 39,8 | | | sp\|P0C1M0\|ATPG_MAIZE | | ATP synthase subunit gamma, chloroplastic; F-ATPase gamma subunit; | 1,75 |
| 10 | -3,5 | 6,1 | 5,1 | 8 | | 1 | | 1 | | | 33 | | | sp\|P27337\|PER1_HORVU | | Peroxidase 1; EC 1.11.1.7; | 0,89 |
| 11 | -3,4 | 5,7 | 2,9 | 4 | | 1 | | 1 | | | 47,1 | | | tr\|Q9SNK3\|Q9SNK3_ORYSJ | | Glyceraldehyde-3-phosphate dehydrogenase B, chloroplast | 0,89 |
| 12 | -3,1 | 5,4 | 3,4 | 5 | | 1 | | 1 | | | 42 | | | sp\|Q40677\|ALFC_ORYSJ | | Fructose-bisphosphate aldolase, chloroplastic; ALDP; EC 4.1.2.13; | 0,89 |
| 13 | -2,7 | 5,7 | 4,7 | 6 | | 1 | | 1 | | | 35,2 | | | sp\|A6MMM0\|CYF_DIOEL | | Apocytochrome f; | 0,89 |
| 15 | -2,1 | 5,8 | 3,2 | 4 | | 1 | | 1 | | | 44,5 | | | sp\|P37833\|AATC_ORYSJ | | Aspartate aminotransferase, cytoplasmic; EC 2.6.1.1; Transaminase A; | 0,89 |
| 19 | -1,3 | 6,7 | 3,3 | 5 | | 1 | | 3 | | | 35,6 | | | sp\|Q08062\|MDHC_MAIZE | | Malate dehydrogenase, cytoplasmic; EC 1.1.1.37; | 2,67 |
| 1 | -11 | 6,2 | 6,1 | 9 | | 2 | | 2 | | | 41 | | | tr\|Q0DJC0\|Q0DJC0_ORYSJ | | Os05g0302700 protein; cDNA clone:001-036-B04, full insert sequence; | 1,75 |
| 2 | -8,2 | 6,2 | 5 | 12 | | 3 | | 3 | | | 49,2 | | | tr\|Q8WL39\|Q8WL39_9ASPA | | Ribulose-bisphosphate carboxylase large subunit; | 2,67 |
| 3 | -2,5 | 5,5 | 3 | 3 | | 1 | | 1 | | | 42,3 | | | tr\|Q1EPF8\|Q1EPF8_MUSAC | | Phosphoglycerate kinase 2; EC 2.7.2.3; | 0,89 |
| 4 | -2,5 | 5,7 | 3,9 | 6 | | 1 | | 1 | | | 29,6 | | | sp\|Q6L5I5\|VDAC2_ORYSJ | | Mitochondrial outer membrane protein porin 2; OsVDAC2; | 0,89 |
| 5 | -2,4 | 5,4 | 2 | 2 | | 1 | | 1 | | | 63,8 | | | tr\|Q6ZFJ9\|Q6ZFJ9_ORYSJ | | 60 kDa chaperonin beta subunit; Os02g0102900 protein; | 0,89 |
| 6 | -2 | 6,1 | 4,9 | 6 | | 1 | | 2 | | | 34,4 | | | tr\|F2CRK1\|F2CRK1_HORVD | | Predicted protein; | 1,75 |
| 9 | -1,3 | 5,2 | 5,5 | 8 | | 1 | | 2 | | | 29,8 | | | tr\|G0YLW6\|G0YLW6_9ARAE | | Putative chlorophyll a/b binding protein; | 1,75 |
| 1 | -24 | 6,9 | 16 | 26 | | 4 | | 12 | | | 27,7 | | | tr\|Q6WFB1\|Q6WFB1_MAIZE | | Photosystem II subunit PsbS | 10,52 |
| 2 | -5 | 6,5 | 7,1 | 12 | | 1 | | 1 | | | 21,9 | | | sp\|P36213\|PSAD_HORVU | | Photosystem I reaction center subunit II, chloroplastic; Photosystem I 20 kDa subunit; | 0,89 |
| 7 | -1,7 | 5,9 | 4,5 | 5 | | 1 | | 1 | | | 33,2 | | | sp\|P08477\|G3PC_HORVU | | Glyceraldehyde-3-phosphate dehydrogenase, cytosolic; EC 1.2.1.12; | 0,89 |
| 1 | -1,7 | 5,5 | 12 | 13 | | 1 | | 1 | | | 9,3 | | | tr\|I1IWU7\|I1IWU7_BRADI | | Uncharacterized protein; | 0,89 |
| 2 | -1,7 | 5,6 | 12 | 27 | | 1 | | 1 | | | 9,4 | | | sp\|A1EA25\|PSBE_AGRST | | no protein information available | 0,89 |
|  |  |  |  |  | |  | |  | | |  | | |  | |  |  |
| Hypersaline sample 2 (15 days) | | | | | | | | | | | | | | | | |  |
| **rank** | **log(e)** | **log(I)** | **% (m)** | **% (c)** | **unique** | | **total** | | | **Mr** | | | **Accession** | | | **Description** |  |
| 1 | -38 | 6,9 | 11 | 24 | 6 | | 10 | | | 50,4 | | | tr\|Q6L9Z6\|Q6L9Z6_9LILI | | | RuBisCO large subunit; | 13,88 |
| 2 | -3,5 | 5,0 | 2,2 | 7 | 1 | | 1 | | | 56 | | | sp\|A9LYC6\|PSBB_ACOAM | | | Photosystem II CP47 chlorophyll apoprotein; | 1,38 |
| 3 | -2,2 | 5,4 | 2 | 3 | 1 | | 1 | | | 71,5 | | | tr\|C5YWM8\|C5YWM8_SORBI | | | no protein information available | 1,38 |
| 4 | -2,7 | 5,9 | 4,5 | 5 | 1 | | 1 | | | 33,2 | | | sp\|P08477\|G3PC_HORVU | | | Glyceraldehyde-3-phosphate dehydrogenase, cytosolic; EC 1.2.1.12; | 1,38 |
| 5 | -1,9 | 5,9 | 3 | 5 | 1 | | 1 | | | 55,3 | | | sp\|A9LYH0\|ATPA_ACOAM | | | EC 3.6.3.14; ATP synthase F1 sector subunit alpha; F-ATPase subunit alpha; | 1,38 |
| 1 | -42 | 6,5 | 12 | 17 | 6 | | 10 | | | 53,6 | | | tr\|H2CPP4\|H2CPP4_COLES | | | EC 3.6.3.14; ATP synthase F1 sector subunit beta; F-ATPase subunit beta; | 13,88 |
| 2 | -39 | 7,8 | 4 | 5 | 1 | | 1 | | | 59,1 | | | tr\|H6THB0\|H6THB0_9LILI | | | ATP synthase subunit beta; EC 3.6.3.14; | 1,31 |
| 3 | -39 | 7,1 | 7 | 16 | 4 | | 8 | | | 49 | | | tr\|B5RHG8\|B5RHG8_9ASPA | | | Ribulose-bisphosphate carboxylase large subunit; | 11,11 |
| 4 | -35 | 7,5 | 9,7 | 18 | 3 | | 5 | | | 55,3 | | | sp\|A9LYH0\|ATPA_ACOAM | | | EC 3.6.3.14; ATP synthase F1 sector subunit alpha; F-ATPase subunit alpha; | 6,92 |
| 5 | -27 | 6,5 | 4,7 | 11 | 2 | | 2 | | | 51,6 | | | tr\|B0B735\|B0B735_9POAL | | | Ribulose-1:p,5-bisphosphate carboxylase/oxygenase large subunit; EC 4.1.1.39; | 2,77 |
| 6 | -28 | 6,3 | 2,1 | 2 | 1 | | 1 | | | 55,1 | | | tr\|F8RS97\|F8RS97_JUNEF | | | ATP synthase subunit alpha | 1,38 |
| 7 | -27 | 6,8 | 2,1 | 2 | 1 | | 3 | | | 50,4 | | | tr\|Q6L9Z6\|Q6L9Z6_9LILI | | | RuBisCO large subunit; | 4,16 |
| 8 | -21 | 6,8 | 2 | 2 | 1 | | 1 | | | 55,3 | | | sp\|P62626\|ATPB_AEGCO | | | EC 3.6.3.14; ATP synthase F1 sector subunit beta; | 1,38 |
| 9 | -19 | 6,1 | 7,4 | 9 | 3 | | 4 | | | 59,1 | | | sp\|P19023\|ATPBM_MAIZE | | | ATP synthase subunit beta, mitochondrial; EC 3.6.3.14; | 5,55 |
| 10 | -6,0 | 5,5 | 2 | 2 | 1 | | 2 | | | 63,8 | | | tr\|Q6ZFJ9\|Q6ZFJ9_ORYSJ | | | 60 kDa chaperonin beta subunit; Os02g0102900 protein; | 2,77 |
| 1 | -23 | 6,8 | 7,9 | 9 | 3 | | 3 | | | 50 | | | tr\|Q1ENY9\|Q1ENY9_MUSAC | | | Phosphoglycerate kinase, chloroplast, putative; EC 2.7.2.3; | 4,16 |
| 2 | -22 | 6,6 | 3,3 | 3 | 1 | | 1 | | | 50 | | | tr\|B6STH5\|B6STH5_MAIZE | | | Phosphoglycerate kinase; EC 2.7.2.3; | 1,38 |
| 3 | -10 | 6,7 | 14 | 19 | 2 | | 3 | | | 20,8 | | | tr\|F8UCA0\|F8UCA0_9LILI | | | Glyceraldehyde-3-phosphate dehydrogenase; EC 1.2.1.12; | 5,55 |
| 4 | -9 | 6,5 | 6,7 | 10 | 2 | | 2 | | | 42,7 | | | tr\|F2D714\|F2D714_HORVD | | | Predicted protein; | 2,77 |
| 5 | -2,1 | 5,1 | 5,3 | 6 | 1 | | 1 | | | 31,5 | | | tr\|G3FBL3\|G3FBL3_9LILI | | | Actin; Flags: Fragment | 1,38 |
| 7 | -1,4 | 5,5 | 5,1 | 6 | 2 | | 2 | | | 37 | | | tr\|Q7XZW5\|Q7XZW5_ORYSJ | | | Malate dehydrogenase; EC 1.1.1.37 | 2,77 |
| 12 | -1,3 | 5,4 | 3,1 | 5 | 1 | | 1 | | | 39,8 | | | sp\|P0C1M0\|ATPG_MAIZE | | | ATP synthase subunit gamma, chloroplastic; F-ATPase gamma subunit; | 1,38 |
| 1 | -3,4 | 5,1 | 4,3 | 6 | 1 | | 1 | | | 26,7 | | | sp\|P34937\|TPIS_HORVU | | | Triosephosphate isomerase, cytosolic; TIM; Triose-phosphate isomerase; EC 5.3.1.1 | 1,38 |
| 2 | -2,5 | 5,3 | 5 | 6 | 1 | | 2 | | | 34,4 | | | tr\|F2CRK1\|F2CRK1_HORVD | | | Predicted protein; | 2,77 |
| 3 | -1,7 | 5,0 | 5 | 6 | 1 | | 1 | | | 26 | | | tr\|Q6YTY2\|Q6YTY2_ORYSJ | | | Os07g0608500 protein; Putative 40S ribosomal protein; | 1,38 |
| 4 | -1,5 | 5,5 | 2,7 | 4 | 1 | | 1 | | | 42,4 | | | sp\|P04709\|ADT1_MAIZE | | | ADP:p ,ATP carrier protein 1, mitochondrial; ADP/ATP translocase 1; | 1,38 |
| 1 | -3,4 | 5,4 | 3,4 | 6 | 1 | | 1 | | | 24,8 | | | sp\|P13192\|PSAF_HORVU | | | Light-harvesting complex I 17 kDa protein; | 1,38 |
| 1 | -2,2 | 5,3 | 12 | 28 | 1 | | 2 | | | 9,4 | | | sp\|A1EA25\|PSBE_AGRST | | | Cytochrome b559 subunit alpha; PSII reaction center subunit V; | 2,77 |
|  |  |  |  |  |  | |  | | |  | | |  | | |  |  |
| Hypersaline sample 2 (30 days) | | | | | | | | | | | | | | | | |  |
| **rank** | **log(e)** | **log(I)** | **% (m)** | **% (c)** | **unique** | | **total** | | **Mr** | | | **Accession** | | | **Description** | | **% of spectra** |
| 1 | -17 | 6,2 | 8,1 | 12 | 4 | | 4 | | 41,7 | | | tr\|C7IWD0\|C7IWD0_ORYSJ | | | Os01g0791600 protein; | | 4,82 |
| 2 | -9,1 | 6,0 | 4,3 | 8 | 2 | | 3 | | 55,3 | | | sp\|A9LYH0\|ATPA_ACOAM | | | EC 3.6.3.14; ATP synthase F1 sector subunit alpha; F-ATPase subunit alpha; | | 3,61 |
| 3 | -7,5 | 6,3 | 2,2 | 2 | 5 | | 8 | | 50,4 | | | tr\|Q6L9Z6\|Q6L9Z6_9LILI | | | RuBisCO large subunit; | | 9,64 |
| 4 | -4,1 | 5,0 | 3,8 | 4 | 2 | | 2 | | 48,1 | | | sp\|P42895\|ENO2_MAIZE | | | no protein information available | | 2,41 |
| 6 | -2,3 | 5,1 | 1,5 | 2 | 2 | | 2 | | 104,8 | | | sp\|Q7XPY2\|PMA1_ORYSJ | | | no protein information available | | 2,41 |
| 7 | -2,4 | 5,6 | 3,6 | 11 | 1 | | 1 | | 50,8 | | | tr\|G1C6J9\|G1C6J9_9LILI | | | no protein information available | | 1,20 |
| 10 | -1,7 | 5,4 | 1,2 | 2 | 4 | | 5 | | 79,5 | | | sp\|Q06572\|AVP_HORVU | | | EC 3.6.1.1; Pyrophosphate-energized inorganic pyrophosphatase; | | 6,02 |
| 1 | -52 | 6,79 | 17 | 22 | 6 | | 10 | | 53,6 | | | sp\|A9L9A3\|ATPB_LEMMI | | | EC 3.6.3.14; ATP synthase F1 sector subunit beta; F-ATPase subunit beta; | | 12,05 |
| 2 | -51 | 6,4 | 3,2 | 3 | 1 | | 1 | | 53,6 | | | tr\|H2CPP4\|H2CPP4_COLES | | | EC 3.6.3.14; ATP synthase F1 sector subunit beta; F-ATPase subunit beta; | | 1,20 |
| 3 | -35 | 6,97 | 9,7 | 18 | 4 | | 5 | | 55,3 | | | sp\|A9LYH0\|ATPA_ACOAM | | | EC 3.6.3.14; ATP synthase F1 sector subunit alpha; F-ATPase subunit alpha; | | 6,02 |
| 4 | -11 | 5,7 | 7 | 8 | 3 | | 3 | | 48,1 | | | sp\|P42895\|ENO2_MAIZE | | | Enolase 2; EC 4.2.1.11; 2-phospho-D-glycerate hydro-lyase 2; | | 3,61 |
| 5 | -11 | 6,3 | 4,7 | 6 | 2 | | 3 | | 59,1 | | | sp\|P19023\|ATPBM_MAIZE | | | ATP synthase subunit beta, mitochondrial; EC 3.6.3.14; | | 3,61 |
| 6 | -9,1 | 6,5 | 4,4 | 10 | 6 | | 10 | | 50,4 | | | tr\|Q6L9Z6\|Q6L9Z6_9LILI | | | RuBisCO large subunit; | | 12,05 |
| 7 | -3,4 | 5,6 | 2 | 2 | 1 | | 2 | | 63,8 | | | tr\|Q6ZFJ9\|Q6ZFJ9_ORYSJ | | | 60 kDa chaperonin beta subunit; Os02g0102900 protein; | | 2,41 |
| 12 | -1,7 | 5,5 | 2,1 | 3 | 1 | | 1 | | 61,4 | | | tr\|Q7X9A7\|Q7X9A7_ORYSJ | | | 60 kDa chaperonin alpha subunit; | | 1,20 |
| 1 | -22 | 6,2 | 8,8 | 11 | 3 | | 4 | | 49,8 | | | tr\|B6STH5\|B6STH5_MAIZE | | | Phosphoglycerate kinase; EC 2.7.2.3; | | 4,82 |
| 2 | -22 | 6,5 | 2,5 | 3 | 1 | | 1 | | 50 | | | tr\|Q1ENY9\|Q1ENY9_MUSAC | | | Phosphoglycerate kinase, chloroplast, putative; EC 2.7.2.3; | | 1,20 |
| 3 | -12 | 6,2 | 4 | 4 | 1 | | 1 | | 0 | | | tr\|Q655T1\|Q655T1_ORYSJ | | | no protein information available | | 1,20 |
| 4 | -12 | 6,8 | 14 | 19 | 2 | | 3 | | 20,8 | | | tr\|F8UCA0\|F8UCA0_9LILI | | | Glyceraldehyde-3-phosphate dehydrogenase; EC 1.2.1.12; | | 3,61 |
| 5 | -9,7 | 6,5 | 3,9 | 4 | 1 | | 1 | | 20,1 | | | tr\|Q7FAH2\|Q7FAH2_ORYSJ | | | Glyceraldehyde-3-phosphate dehydrogenase 2, cytosolic; EC 1.2.1.12 | | 1,20 |
| 6 | -2,8 | 6,0 | 5,1 | 8 | 1 | | 1 | | 33 | | | sp\|P27337\|PER1_HORVU | | | Peroxidase 1; EC 1.11.1.7; | | 1,20 |
| 7 | -2,0 | 5,2 | 3,2 | 5 | 1 | | 1 | | 42,7 | | | tr\|F2D714\|F2D714_HORVD | | | Predicted protein; | | 1,20 |
| 11 | -1,6 | 6,0 | 3,3 | 8 | 1 | | 2 | | 50,1 | | | sp\|P25776\|ORYA_ORYSJ | | | no protein information available | | 2,41 |
| 1 | -9,5 | 6,3 | 9,8 | 12 | 2 | | 4 | | 34,4 | | | tr\|F2CRK1\|F2CRK1_HORVD | | | Predicted protein; | | 4,82 |
| 2 | -4,7 | 5,7 | 3,2 | 5 | 1 | | 1 | | 41 | | | tr\|Q0DJC0\|Q0DJC0_ORYSJ | | | Os05g0302700 protein; | | 1,20 |
| 3 | -4,7 | 5,7 | 8,5 | 12 | 1 | | 1 | | 24,7 | | | tr\|F2DTJ2\|F2DTJ2_HORVD | | | Predicted protein | | 1,20 |
| 4 | -3,5 | 5,9 | 5,1 | 8 | 1 | | 1 | | 33 | | | sp\|P27337\|PER1_HORVU | | | Peroxidase 1; EC 1.11.1.7; | | 1,20 |
| 7 | -3 | 5,9 | 5,4 | 7 | 1 | | 1 | | 29,8 | | | tr\|G0YLW6\|G0YLW6_9ARAE | | | Putative chlorophyll a/b binding protein; | | 1,20 |
| 1 | -2,8 | 6,3 | 3,8 | 6 | 1 | | 2 | | 24,8 | | | sp\|P13192\|PSAF_HORVU | | | Light-harvesting complex I 17 kDa protein; PSI-F; | | 2,41 |
| 2 | -2,5 | 5,5 | 5,7 | 9 | 1 | | 1 | | 27,7 | | | tr\|Q6WFB1\|Q6WFB1_MAIZE | | | Photosystem II subunit PsbS; | | 1,20 |
| 1 | -1,6 | 5,6 | 13 | 28 | 1 | | 2 | | 9,4 | | | sp\|A1EA25\|PSBE_AGRST | | | Cytochrome b559 subunit alpha; PSII reaction center subunit V; | | 2,41 |

| Control sample 3 | | | | | | | | | | | | |  | |
| --- | --- | --- | --- | --- | --- | --- | --- | --- | --- | --- | --- | --- | --- | --- |
| **rank** | | **log (e)** | **log (I)** | **% (m)** | **% (c)** | **unique** | **total** | | **Mr** | **Accession** | | **Description** | **% of spectra** | |
| 1 | | -9 | 7,1 | 4,2 | 9 | 3 | 5 | | 50,4 | tr\|Q6L9Z6\|Q6L9Z6_9LILI | | RuBisCO large subunit; | 4,10 | |
| 2 | | -3,8 | 5,5 | 2,1 | 3 | 1 | 1 | | 74 | tr\|Q2QV45\|Q2QV45_ORYSJ | | 70 kDa heat shock protein; Os12g0244100 protein; | 0,82 | |
| 3 | | -3,4 | 6,1 | 4,3 | 5 | 1 | 3 | | 33,2 | sp\|P08477\|G3PC_HORVU | | Glyceraldehyde-3-phosphate dehydrogenase, cytosolic; | 2,46 | |
| 4 | | -1,7 | 5,8 | 1,6 | 2 | 1 | 1 | | 72,9 | sp\|Q7SIC9\|TKTC_MAIZE | | Transketolase:p , chloroplastic; TK; EC 2.2.1.1; | 0,82 | |
| 5 | | -1,8 | 5,7 | 1,5 | 2 | 1 | 1 | | 111,4 | tr\|Q6V9T1\|Q6V9T1_ORYSJ | | Glycine dehydrogenase P protein; Os01g0711400 protein; | 0,82 | |
| 1 | | -55 | 7,5 | 17 | 21 | 6 | 13 | | 53,6 | tr\|H2CPP4\|H2CPP4_COLES | | EC 3.6.3.14; ATP synthase F1 sector subunit beta; | 10,66 | |
| 2 | | -47 | 7,5 | 3,2 | 3 | 1 | 1 | | 59,1 | tr\|Q4FGI4\|Q4FGI4_TYPLA | | ATP synthase subunit beta; EC 3.6.3.14; | 0,82 | |
| 3 | | -41 | 7,3 | 2 | 2 | 1 | 2 | | 55,3 | sp\|Q95AD6\|ATPB_WHIBI | | EC 3.6.3.14; ATP synthase F1 sector subunit beta; | 1,64 | |
| 4 | | -37 | 7,2 | 12 | 21 | 5 | 8 | | 55,3 | sp\|A9LYH0\|ATPA_ACOAM | | EC 3.6.3.14; ATP synthase F1 sector subunit alpha; | 6,56 | |
| 5 | | -37 | 7,5 | 11 | 24 | 5 | 8 | | 50,4 | tr\|Q6L9Z6\|Q6L9Z6_9LILI | | RuBisCO large subunit; | 6,56 | |
| 6 | | -29 | 7,2 | 2 | 2 | 1 | 1 | | 55,3 | sp\|P62626\|ATPB_AEGCO | | EC 3.6.3.14; ATP synthase F1 sector subunit beta; | 0,82 | |
| 7 | | -18 | 6,7 | 2,3 | 2 | 4 | 6 | | 50,4 | tr\|C6G4V9\|C6G4V9_9ASPA | | Ribulose-1:p,5-bisphosphate carboxylase/oxygenase large subunit; | 4,92 | |
| 8 | | -17 | 6,0 | 6,3 | 8 | 4 | 5 | | 59,1 | sp\|P19023\|ATPBM_MAIZE | | ATP synthase subunit beta, mitochondrial; EC 3.6.3.14; | 4,10 | |
| 9 | | -9,1 | 5,7 | 4,6 | 6 | 3 | 4 | | 61,4 | tr\|Q7X9A7\|Q7X9A7_ORYSJ | | 60 kDa chaperonin alpha subunit; Putative rubisco subunit binding-protein alpha subunit; | 3,28 | |
| 10 | | -5,0 | 5,0 | 4,1 | 6 | 1 | 1 | | 55,1 | sp\|P05494\|ATPAM_MAIZE | | ATP synthase subunit alpha, mitochondrial; | 0,82 | |
| 1 | | -23 | 6,9 | 10 | 15 | 3 | 5 | | 42,7 | tr\|F2D714\|F2D714_HORVD | | Predicted protein; | 4,10 | |
| 2 | | -21 | 6,5 | 7,9 | 9 | 3 | 4 | | 50 | tr\|Q1ENY9\|Q1ENY9_MUSAC | | Phosphoglycerate kinase, chloroplast, putative; EC 2.7.2.3; | 3,28 | |
| 3 | | -23 | 6,9 | 3,5 | 4 | 2 | 2 | | 0 | sp\|P12782\|PGKH_WHEAT | | no protein information available | 1,64 | |
| 4 | | -22 | 6,9 | 1,9 | 2 | 1 | 1 | | 50 | tr\|B6STH5\|B6STH5_MAIZE | | Phosphoglycerate kinase; EC 2.7.2.3; | 0,82 | |
| 5 | | -15 | 6,0 | 7,8 | 10 | 3 | 3 | | 53,6 | sp\|Q3V527\|ATPB_ACOCL | | EC 3.6.3.14; ATP synthase F1 sector subunit beta; | 2,46 | |
| 6 | | -12 | 6,4 | 5,4 | 6 | 1 | 1 | | 31,4 | tr\|C1JYE2\|C1JYE2_9POAL | | Phosphoglycerate kinase; EC 2.7.2.3; | 0,82 | |
| 7 | | -7,5 | 6,3 | 5 | 12 | 2 | 2 | | 48,9 | tr\|O78641\|O78641_9ASPA | | no protein information available | 1,64 | |
| 8 | | -4,8 | 5,6 | 4,2 | 6 | 1 | 1 | | 47,2 | sp\|Q42450\|RCAB_HORVU | | no protein information available | 0,82 | |
| 9 | | -3,3 | 6,0 | 3,3 | 5 | 1 | 2 | | 39,8 | sp\|P0C1M0\|ATPG_MAIZE | | ATP synthase subunit gamma, chloroplastic; F-ATPase gamma subunit; | 1,64 | |
| 10 | | -3,6 | 6,2 | 5,1 | 8 | 1 | 1 | | 33 | sp\|P27337\|PER1_HORVU | | Peroxidase 1; EC 1.11.1.7; | 0,82 | |
| 11 | | -3,5 | 5,8 | 2,9 | 4 | 1 | 1 | | 47,1 | tr\|Q9SNK3\|Q9SNK3_ORYSJ | | Glyceraldehyde-3-phosphate dehydrogenase B, chloroplast | 0,82 | |
| 12 | | -3,2 | 5,5 | 3,4 | 5 | 1 | 1 | | 42 | sp\|Q40677\|ALFC_ORYSJ | | Fructose-bisphosphate aldolase, chloroplastic; ALDP; EC 4.1.2.13; | 0,82 | |
| 13 | | -2,5 | 5,5 | 4,7 | 6 | 1 | 1 | | 35,2 | sp\|A6MMM0\|CYF_DIOEL | | Apocytochrome f; | 0,82 | |
| 15 | | -2,2 | 5,9 | 3,2 | 4 | 1 | 1 | | 44,5 | sp\|P37833\|AATC_ORYSJ | | Aspartate aminotransferase, cytoplasmic; EC 2.6.1.1; Transaminase A; | 0,82 | |
| 19 | | -1,5 | 6,9 | 3,3 | 5 | 1 | 3 | | 35,6 | sp\|Q08062\|MDHC_MAIZE | | Malate dehydrogenase, cytoplasmic; EC 1.1.1.37; | 2,46 | |
| 1 | | -12 | 6,3 | 6,1 | 9 | 2 | 2 | | 41 | tr\|Q0DJC0\|Q0DJC0_ORYSJ | | Os05g0302700 protein; cDNA clone:001-036-B04, full insert sequence; | 1,64 | |
| 2 | | -8,3 | 6,3 | 5 | 12 | 5 | 8 | | 49,2 | tr\|Q8WL39\|Q8WL39_9ASPA | | Ribulose-bisphosphate carboxylase large subunit; | 6,56 | |
| 3 | | -2,7 | 5,7 | 3 | 3 | 1 | 1 | | 42,3 | tr\|Q1EPF8\|Q1EPF8_MUSAC | | Phosphoglycerate kinase 2; EC 2.7.2.3; | 0,82 | |
| 4 | | -2,6 | 5,6 | 3,9 | 6 | 1 | 1 | | 29,6 | sp\|Q6L5I5\|VDAC2_ORYSJ | | Mitochondrial outer membrane protein porin 2; OsVDAC2; | 0,82 | |
| 5 | | -2,5 | 5,5 | 2 | 2 | 1 | 1 | | 63,8 | tr\|Q6ZFJ9\|Q6ZFJ9_ORYSJ | | 60 kDa chaperonin beta subunit; Os02g0102900 protein; | 0,82 | |
| 6 | | -2 | 6,1 | 4,9 | 6 | 1 | 2 | | 34,4 | tr\|F2CRK1\|F2CRK1_HORVD | | Predicted protein; | 1,64 | |
| 9 | | -1,3 | 5,5 | 5,7 | 8 | 1 | 2 | | 29,8 | tr\|G0YLW6\|G0YLW6_9ARAE | | Putative chlorophyll a/b binding protein; | 1,64 | |
| 1 | | -25 | 6,9 | 15 | 25 | 5 | 12 | | 27,7 | tr\|Q6WFB1\|Q6WFB1_MAIZE | | Photosystem II subunit PsbS | 9,84 | |
| 2 | | -4 | 5,5 | 6,8 | 12 | 1 | 1 | | 21,9 | sp\|P36213\|PSAD_HORVU | | Photosystem I reaction center subunit II, chloroplastic; Photosystem I 20 kDa subunit; | 0,82 | |
| 7 | | -1,3 | 5,6 | 4,3 | 5 | 1 | 1 | | 33,2 | sp\|P08477\|G3PC_HORVU | | Glyceraldehyde-3-phosphate dehydrogenase, cytosolic; EC 1.2.1.12; | 0,82 | |
| 1 | | -1,8 | 5,6 | 13 | 14 | 1 | 1 | | 9,3 | tr\|I1IWU7\|I1IWU7_BRADI | | Uncharacterized protein; | 0,82 | |
| 2 | | -1,2 | 5,2 | 13 | 28 | 1 | 1 | | 9,4 | sp\|A1EA25\|PSBE_AGRST | | no protein information available | 0,82 | |
|  | |  |  |  |  |  |  | |  |  | |  |  | |
| Hypersaline sample 3 (15 days) | | | | | | | | | | | | |  | |
| **rank** | | **log(e)** | **log(I)** | **% (m)** | **% (c)** | **unique** | **total** | | **Mr** | **Accession** | | **Description** |  | |
| 1 | | -35 | 6,5 | 11 | 24 | 6 | 10 | | 50,4 | tr\|Q6L9Z6\|Q6L9Z6_9LILI | | RuBisCO large subunit; | 13,89 | |
| 2 | | -3,2 | 5,2 | 2,6 | 7 | 1 | 1 | | 56 | sp\|A9LYC6\|PSBB_ACOAM | | Photosystem II CP47 chlorophyll apoprotein; | 1,39 | |
| 3 | | -2,4 | 5,3 | 2 | 3 | 1 | 1 | | 71,5 | tr\|C5YWM8\|C5YWM8_SORBI | | no protein information available | 1,39 | |
| 4 | | -2,1 | 5,2 | 4,3 | 5 | 1 | 1 | | 33,2 | sp\|P08477\|G3PC_HORVU | | Glyceraldehyde-3-phosphate dehydrogenase, cytosolic; EC 1.2.1.12; | 1,39 | |
| 5 | | -1,4 | 5,6 | 3 | 5 | 1 | 1 | | 55,3 | sp\|A9LYH0\|ATPA_ACOAM | | EC 3.6.3.14; ATP synthase F1 sector subunit alpha; F-ATPase subunit alpha; | 1,39 | |
| 1 | | -44 | 6,4 | 14 | 17 | 5 | 9 | | 53,6 | tr\|H2CPP4\|H2CPP4_COLES | | EC 3.6.3.14; ATP synthase F1 sector subunit beta; F-ATPase subunit beta; | 12,50 | |
| 2 | | -42 | 6,6 | 5 | 5 | 1 | 1 | | 59,1 | tr\|H6THB0\|H6THB0_9LILI | | ATP synthase subunit beta; EC 3.6.3.14; | 1,39 | |
| 3 | | -37 | 6,5 | 7 | 16 | 4 | 8 | | 49 | tr\|B5RHG8\|B5RHG8_9ASPA | | Ribulose-bisphosphate carboxylase large subunit; | 11,11 | |
| 4 | | -31 | 6,4 | 9,7 | 18 | 4 | 6 | | 55,3 | sp\|A9LYH0\|ATPA_ACOAM | | EC 3.6.3.14; ATP synthase F1 sector subunit alpha; F-ATPase subunit alpha; | 8,33 | |
| 5 | | -28 | 6,5 | 4,7 | 11 | 2 | 3 | | 51,6 | tr\|B0B735\|B0B735_9POAL | | Ribulose-1:p,5-bisphosphate carboxylase/oxygenase large subunit; EC 4.1.1.39; | 4,17 | |
| 6 | | -27 | 6,7 | 2,2 | 2 | 1 | 1 | | 55,1 | tr\|F8RS97\|F8RS97_JUNEF | | ATP synthase subunit alpha | 1,39 | |
| 7 | | -28 | 7,0 | 2,2 | 2 | 1 | 3 | | 50,4 | tr\|Q6L9Z6\|Q6L9Z6_9LILI | | RuBisCO large subunit; | 4,17 | |
| 8 | | -22 | 6,9 | 2 | 2 | 1 | 1 | | 55,3 | sp\|P62626\|ATPB_AEGCO | | EC 3.6.3.14; ATP synthase F1 sector subunit beta; | 1,39 | |
| 9 | | -17 | 6,3 | 7,4 | 9 | 3 | 4 | | 59,1 | sp\|P19023\|ATPBM_MAIZE | | ATP synthase subunit beta, mitochondrial; EC 3.6.3.14; | 5,56 | |
| 10 | | -6,4 | 6,1 | 2 | 2 | 1 | 2 | | 63,8 | tr\|Q6ZFJ9\|Q6ZFJ9_ORYSJ | | 60 kDa chaperonin beta subunit; Os02g0102900 protein; | 2,78 | |
| 1 | | -23 | 6,6 | 7,9 | 9 | 3 | 3 | | 50 | tr\|Q1ENY9\|Q1ENY9_MUSAC | | Phosphoglycerate kinase, chloroplast, putative; EC 2.7.2.3; | 4,17 | |
| 2 | | -21 | 6,5 | 3,3 | 3 | 1 | 1 | | 50 | tr\|B6STH5\|B6STH5_MAIZE | | Phosphoglycerate kinase; EC 2.7.2.3; | 1,39 | |
| 3 | | -9,9 | 6,7 | 14 | 19 | 2 | 3 | | 20,8 | tr\|F8UCA0\|F8UCA0_9LILI | | Glyceraldehyde-3-phosphate dehydrogenase; EC 1.2.1.12; | 4,17 | |
| 4 | | -9 | 6,5 | 6,7 | 10 | 2 | 2 | | 42,7 | tr\|F2D714\|F2D714_HORVD | | Predicted protein; | 2,78 | |
| 5 | | -2,7 | 5,3 | 5,3 | 6 | 1 | 1 | | 31,5 | tr\|G3FBL3\|G3FBL3_9LILI | | Actin; Flags: Fragment | 1,39 | |
| 7 | | -1,7 | 5,7 | 5,1 | 6 | 1 | 1 | | 37 | tr\|Q7XZW5\|Q7XZW5_ORYSJ | | Malate dehydrogenase; EC 1.1.1.37 | 1,39 | |
| 12 | | -1,4 | 6,2 | 3,3 | 5 | 1 | 1 | | 39,8 | sp\|P0C1M0\|ATPG_MAIZE | | ATP synthase subunit gamma, chloroplastic; F-ATPase gamma subunit; | 1,39 | |
| 1 | | -3,4 | 5,4 | 4,3 | 6 | 1 | 1 | | 26,7 | sp\|P34937\|TPIS_HORVU | | Triosephosphate isomerase, cytosolic; TIM; Triose-phosphate isomerase; EC 5.3.1.1 | 1,39 | |
| 2 | | -2,5 | 5,9 | 4,9 | 6 | 1 | 2 | | 34,4 | tr\|F2CRK1\|F2CRK1_HORVD | | Predicted protein; | 2,78 | |
| 3 | | -1,7 | 5,1 | 5,2 | 6 | 1 | 1 | | 26 | tr\|Q6YTY2\|Q6YTY2_ORYSJ | | Os07g0608500 protein; Putative 40S ribosomal protein; | 1,39 | |
| 4 | | -1,7 | 5,7 | 2,8 | 4 | 1 | 1 | | 42,4 | sp\|P04709\|ADT1_MAIZE | | ADP:p ,ATP carrier protein 1, mitochondrial; ADP/ATP translocase 1; | 1,39 | |
| 1 | | -3,7 | 5,7 | 3,8 | 6 | 1 | 1 | | 24,8 | sp\|P13192\|PSAF_HORVU | | Light-harvesting complex I 17 kDa protein; | 1,39 | |
| 1 | | -2,4 | 5,8 | 13 | 28 | 1 | 2 | | 9,4 | sp\|A1EA25\|PSBE_AGRST | | Cytochrome b559 subunit alpha; PSII reaction center subunit V; | 2,78 | |
|  | |  |  |  |  |  |  | |  |  | |  |  | |
| Hypersaline sample 3 (30 days) | | | | | | | | | | | | |  | |
| **rank** | | **log(e)** | **log(I)** | **% (m)** | **% (c)** | **unique** | **total** | | **Mr** | | **Accession** | **Description** | **% spectra** | |
| 1 | | -15 | 6,0 | 8,2 | 12 | 4 | 5 | | 41,7 | | tr\|C7IWD0\|C7IWD0_ORYSJ | Os01g0791600 protein; | 6,58 | |
| 2 | | -9,5 | 6,5 | 4,3 | 8 | 2 | 3 | | 55,3 | | sp\|A9LYH0\|ATPA_ACOAM | EC 3.6.3.14; ATP synthase F1 sector subunit alpha; F-ATPase subunit alpha; | 3,95 | |
| 3 | | -7,8 | 6,7 | 2,2 | 2 | 2 | 5 | | 50,4 | | tr\|Q6L9Z6\|Q6L9Z6_9LILI | RuBisCO large subunit; | 6,58 | |
| 4 | | -4,5 | 5,7 | 3,8 | 4 | 1 | 1 | | 48,1 | | sp\|P42895\|ENO2_MAIZE | no protein information available | 1,32 | |
| 6 | | -2,5 | 5,6 | 1,5 | 2 | 1 | 1 | | 104,8 | | sp\|Q7XPY2\|PMA1_ORYSJ | no protein information available | 1,32 | |
| 7 | | -2,5 | 5,7 | 3,5 | 11 | 1 | 1 | | 50,8 | | tr\|G1C6J9\|G1C6J9_9LILI | no protein information available | 1,32 | |
| 10 | | -1,7 | 5,3 | 1,3 | 2 | 4 | 5 | | 79,5 | | sp\|Q06572\|AVP_HORVU | EC 3.6.1.1; Pyrophosphate-energized inorganic pyrophosphatase; | 6,58 | |
| 1 | | -52 | 6,2 | 18 | 22 | 6 | 10 | | 53,6 | | sp\|A9L9A3\|ATPB_LEMMI | EC 3.6.3.14; ATP synthase F1 sector subunit beta; F-ATPase subunit beta; | 13,16 | |
| 2 | | -52 | 6,9 | 3,2 | 3 | 1 | 1 | | 53,6 | | tr\|H2CPP4\|H2CPP4_COLES | EC 3.6.3.14; ATP synthase F1 sector subunit beta; F-ATPase subunit beta; | 1,32 | |
| 3 | | -33 | 6,9 | 9,7 | 18 | 4 | 5 | | 55,3 | | sp\|A9LYH0\|ATPA_ACOAM | EC 3.6.3.14; ATP synthase F1 sector subunit alpha; F-ATPase subunit alpha; | 6,58 | |
| 4 | | -12 | 5,8 | 7 | 8 | 2 | 2 | | 48,1 | | sp\|P42895\|ENO2_MAIZE | Enolase 2; EC 4.2.1.11; 2-phospho-D-glycerate hydro-lyase 2; | 2,63 | |
| 5 | | -11 | 6,2 | 4,7 | 6 | 2 | 3 | | 59,1 | | sp\|P19023\|ATPBM_MAIZE | ATP synthase subunit beta, mitochondrial; EC 3.6.3.14; | 3,95 | |
| 6 | | -9,7 | 6,7 | 4,4 | 10 | 5 | 8 | | 50,4 | | tr\|Q6L9Z6\|Q6L9Z6_9LILI | RuBisCO large subunit; | 10,53 | |
| 7 | | -3,6 | 5,8 | 2 | 2 | 1 | 2 | | 63,8 | | tr\|Q6ZFJ9\|Q6ZFJ9_ORYSJ | 60 kDa chaperonin beta subunit; Os02g0102900 protein; | 2,63 | |
| 12 | | -1,5 | 5,3 | 2,1 | 3 | 1 | 1 | | 61,4 | | tr\|Q7X9A7\|Q7X9A7_ORYSJ | 60 kDa chaperonin alpha subunit; | 1,32 | |
| 1 | | -24 | 6,4 | 8,8 | 11 | 3 | 4 | | 49,8 | | tr\|B6STH5\|B6STH5_MAIZE | Phosphoglycerate kinase; EC 2.7.2.3; | 5,26 | |
| 2 | | -22 | 6,3 | 2,5 | 3 | 1 | 1 | | 50 | | tr\|Q1ENY9\|Q1ENY9_MUSAC | Phosphoglycerate kinase, chloroplast, putative; EC 2.7.2.3; | 1,32 | |
| 3 | | -14 | 6,5 | 4 | 4 | 1 | 1 | | 0 | | tr\|Q655T1\|Q655T1_ORYSJ | no protein information available | 1,32 | |
| 4 | | -10 | 6,5 | 14 | 19 | 2 | 3 | | 20,8 | | tr\|F8UCA0\|F8UCA0_9LILI | Glyceraldehyde-3-phosphate dehydrogenase; EC 1.2.1.12; | 3,95 | |
| 5 | | -9,7 | 6,5 | 3,9 | 4 | 1 | 1 | | 20,1 | | tr\|Q7FAH2\|Q7FAH2_ORYSJ | Glyceraldehyde-3-phosphate dehydrogenase 2, cytosolic; EC 1.2.1.12 | 1,32 | |
| 6 | | -2,9 | 6,1 | 5,1 | 8 | 1 | 1 | | 33 | | sp\|P27337\|PER1_HORVU | Peroxidase 1; EC 1.11.1.7; | 1,32 | |
| 7 | | -2,0 | 5,2 | 3,2 | 5 | 1 | 1 | | 42,7 | | tr\|F2D714\|F2D714_HORVD | Predicted protein; | 1,32 | |
| 11 | | -1,5 | 5,4 | 3,3 | 8 | 1 | 2 | | 50,1 | | sp\|P25776\|ORYA_ORYSJ | no protein information available | 2,63 | |
| 1 | | -9,5 | 6,5 | 9,8 | 12 | 2 | 4 | | 34,4 | | tr\|F2CRK1\|F2CRK1_HORVD | Predicted protein; | 5,26 | |
| 2 | | -4,7 | 5,7 | 3,2 | 5 | 1 | 1 | | 41 | | tr\|Q0DJC0\|Q0DJC0_ORYSJ | Os05g0302700 protein; | 1,32 | |
| 3 | | -4,5 | 5,3 | 8,5 | 12 | 1 | 1 | | 24,7 | | tr\|F2DTJ2\|F2DTJ2_HORVD | Predicted protein | 1,32 | |
| 4 | | -3,2 | 5,7 | 5,1 | 8 | 1 | 1 | | 33 | | sp\|P27337\|PER1_HORVU | Peroxidase 1; EC 1.11.1.7; | 1,32 | |
| 7 | | -2 | 5,7 | 5,4 | 7 | 1 | 1 | | 29,8 | | tr\|G0YLW6\|G0YLW6_9ARAE | Putative chlorophyll a/b binding protein; | 1,32 | |
| 1 | | -2,5 | 6,0 | 3,8 | 6 | 1 | 2 | | 24,8 | | sp\|P13192\|PSAF_HORVU | Light-harvesting complex I 17 kDa protein; PSI-F; | 2,63 | |
| 2 | | -2,3 | 5,2 | 5,7 | 9 | 1 | 1 | | 27,7 | | tr\|Q6WFB1\|Q6WFB1_MAIZE | Photosystem II subunit PsbS; | 1,32 | |
| 1 | | -1,7 | 5,7 | 13 | 28 | 1 | 2 | | 9,4 | | sp\|A1EA25\|PSBE_AGRST | Cytochrome b559 subunit alpha; PSII reaction center subunit V; | 2,63 | |
|  | | | | | | |  | | | | | | | |
|  | | | | | | |  | | | | | | | |
| **rank**: the relative position of a protein in the list. This can change, depending on how the data is sorted. | | | | | | | | | | | | | |  |
| **log(e)**: the base-10 log of the expectation that any particular protein assignment was made at random (E-value). | | | | | | | | | | | | | |  |
| **log(I):** the base-10 log of the sum of the fragment ion intensities in the tandem mass spectra used to make this assignment. | | | | | | | | | | | | | |  |
| **% (m):** percentage of measured, the amino acid coverage of the protein in this assignment | | | | | | | | | | | | | |  |
| **%(c):** percentage of corrected, the amino acid coverage of the protein in this assignment / the coverage corrected for peptide sequences that are unlikely to be observed using normal proteomics methods. | | | | | | | | | | | | | |  |
| **unique**: the number of unique peptide sequences associated with this protein assignment. | | | | | | | | | | | | | |  |
| **total**: the total number of tandem mass spectra that can be assigned to this protein.  **% spectra**: number of spectra assigned to each peptide/total number of spectra in each sample based on the MS/MS spectra collected from data-dependent runs  **Mr**: the molecular mass of the protein sequence, in kiloDaltons | | | | | | | | | | | | | |  |
