# Supplementary material for: The modulation of leaf metabolism plays a role in salt tolerance of Cymodocea nodosa exposed to hypersaline stress in mesocosms
Source: Front Plant Sci. 2015 Jun 26;6:464. doi: 10.3389/fpls.2015.00464 (PMC4482034; doi:10.3389/fpls.2015.00464)
Supplement: Supplementary file 2 [file Data_Sheet_2.DOCX]

**Supplementary Table 2**: Peptide sequences of proteins detected by EC-MS/MS LTQ- Orbitrap XL in control and hypersaline-treated plants of *Cymodocea nodosa* . Attribution of tryptic fragments, aa. residues alignment and protein coverage are showed. A **=** control samples; B**=** hypersaline after 15 days; C**=** hypersaline after 30 days. See

| **A** |  |  |  |  |  |  |  |  |  |
| --- | --- | --- | --- | --- | --- | --- | --- | --- | --- |
| **Rank** | **log (e)** | **log (I)** | **% (m)** | **% (c)** | **unique** | **tot** | **Mr** | **Accession** | **Description** |
| **1** | **-8** | **6,91** | **4,2** | **9** | **2** | **3** | **50,4** | **tr\|Q6L9Z6\|Q6L9Z6_9LILI** | **RuBisCO large subunit;** |
| spectrum | log(e) | log(I) | m+h | delta | z | zeta | Pre | Start | Sequence |
| 1036 | -1,1 | 6,16 | 1261,65 | 1,499 | 2 | 1 | wrdr | 211 | FLFCAEALYK |
| 624 | -1,9 | 6,56 | 1230,63 | 0,594 | 2 | 1 | negr | 429 | DLATEGNEIIR |
| 619 | -1,8 | 6,49 | 1230,63 | 0,903 | 2 | 1 | negr | 429 | DLATEGNEIIR |
| **2** | **-3,9** | **5,61** | **2,1** | **3** | **1** | **1** | **74** | **tr\|Q2QV45\|Q2QV45_ORYSJ** | **70 kDa heat shock protein; Os12g0244100 protein;** |
| spectrum | log(e) | log(I) | m+h | delta | z | zeta | Pre | Start | Sequence |
| 922 | -3,9 | 5,61 | 1723,89 | 1,311 | 2 | 1 | Evlr | 227 | IINEPTAASLAYGFEK |
| **3** | **-3,5** | **6,23** | **4,3** | **5** | **1** | **3** | **33,2** | **sp\|P08477\|G3PC_HORVU** | **Glyceraldehyde-3-phosphate dehydrogenase, cytosolic; EC 1.2.1.12;** |
| spectrum | log(e) | log(I) | m+h | delta | z | zeta | Pre | Start | Sequence |
| 988 | -3,5 | 5,88 | 1498,85 | -0,16 | 2 | 1 | mafr | 205 | VPTVDVSVVDLTVR |
| 983 | -2,3 | 5,71 | 1498,85 | -0,49 | 2 | 1 | mafr | 205 | VPTVDVSVVDLTVR |
| 981 | -1,1 | 5,64 | 1498,85 | 1,335 | 2 | 1 | mafr | 205 | VPTVDVSVVDLTVR |
| **4** | **-1,8** | **5,92** | **1,6** | **2** | **1** | **1** | **72,9** | **sp\|Q7SIC9\|TKTC_MAIZE** | **Transketolase:p , chloroplastic; TK; EC 2.2.1.1;** |
| spectrum | log(e) | log(I) | m+h | delta | z | zeta | Pre | Start | Sequence |
| 450 | -1,8 | 5,92 | 1182,62 | 2,042 | 2 | 1 | Gidk | 648 | FGASAPAGTIYK |
| **5** | **-1,7** | **5,56** | **1,5** | **2** | **1** | **1** | **111** | **tr\|Q6V9T1\|Q6V9T1_ORYSJ** | **Glycine dehydrogenase P protein; Os01g0711400 protein;** |
| spectrum | log(e) | log(I) | m+h | delta | z | zeta | Pre | Start | Sequence |
| 1282 | -1,7 | 5,56 | 1793,97 | 0,267 | 2 | 1 | Glkk | 439 | LGTVTVQELPFFDTVK |
| **1** | **-54** | **7,36** | **17** | **21** | **6** | **13** | **53,6** | **tr\|H2CPP4\|H2CPP4_COLES** | **EC 3.6.3.14; ATP synthase F1 sector subunit beta;** |
| spectrum | log(e) | log(I) | m+h | delta | z | zeta | Pre | Start | Sequence |
| 1271 | -4,1 | 6,05 | 1735,02 | 1,479 | 2 | 1 | Nlgr | 23 | IAQIIGPVLDVAFPPGK |
| 1305 | -3,1 | 5,82 | 1735,02 | 0,942 | 2 | 1 | Nlgr | 23 | IAQIIGPVLDVAFPPGK |
| 1113 | -6,9 | 6,44 | 1955,02 | 0,504 | 2 | 1 | Tlgr | 110 | IFNVLGEPVDNLGPVDTR |
| 1117 | -6,4 | 6,35 | 1955,02 | 0,3 | 2 | 1 | Tlgr | 110 | IFNVLGEPVDNLGPVDTR |
| 1118 | -5 | 6,24 | 1955,02 | 1,063 | 2 | 1 | Tlgr | 110 | IFNVLGEPVDNLGPVDTR |
| 1669 | -1,2 | 5,77 | 1471,86 | 0,89 | 2 | 1 | gvgk | 179 | TVLIMELINNIAK |
| 1679 | -1,2 | 6,04 | 1471,86 | 1,342 | 2 | 1 | gvgk | 179 | TVLIMELINNIAK |
| 1456 | -1,1 | 5,66 | 1487,85 | 1,285 | 2 | 1 | gvgk | 179 | TVLIMELINNIAK |
| 387 | -2,2 | 6,21 | 1517,75 | 0,924 | 2 | 1 | memk | 218 | ESGVINEQNIAESK |
| 392 | -1,8 | 5,89 | 1517,75 | 0,644 | 2 | 1 | memk | 218 | ESGVINEQNIAESK |
| 475 | -2,2 | 5,69 | 1617,8 | 1,76 | 2 | 1 | aesk | 232 | VALVYGQMNEPPGAR |
| 984 | -3,2 | 6,65 | 1433,78 | 0,909 | 2 | 1 | nifr | 278 | FVQAGSEVSALLGR |
| 986 | -2,1 | 6,7 | 1433,78 | 0,324 | 2 | 1 | nifr | 278 | FVQAGSEVSALLGR |
| **2** | **-44** | **7,29** | **3,2** | **3** | **1** | **1** | **59,1** | **tr\|Q4FGI4\|Q4FGI4_TYPLA** | **ATP synthase subunit beta; EC 3.6.3.14;** |
| spectrum | log(e) | log(I) | m+h | delta | z | zeta | pre | Start | Sequence |
| 1311 | -4,1 | 5,62 | 1735,02 | 1,258 | 2 | 1 | nlgr | 23 | IAQIIGPVLDAVFPPGK |
| 1113 | -6,9 | 6,44 | 1955,02 | 0,504 | 2 | 1 | tlgr | 110 | IFNVLGEPVDNLGPVDTR |
| 1117 | -6,4 | 6,35 | 1955,02 | 0,3 | 2 | 1 | tlgr | 110 | IFNVLGEPVDNLGPVDTR |
| 1118 | -5 | 6,24 | 1955,02 | 1,063 | 2 | 1 | tlgr | 110 | IFNVLGEPVDNLGPVDTR |
| 1669 | -1,2 | 5,77 | 1471,86 | 0,89 | 2 | 1 | gvgk | 179 | TVLIMELINNIAK |
| 1679 | -1,2 | 6,04 | 1471,86 | 1,342 | 2 | 1 | gvgk | 179 | TVLIMELINNIAK |
| 1456 | -1,1 | 5,66 | 1487,85 | 1,285 | 2 | 1 | gvgk | 179 | TVLIMELINNIAK |
| 475 | -2,2 | 5,69 | 1617,8 | 1,76 | 2 | 1 | aesk | 232 | VALVYGQMNEPPGAR |
| 984 | -3,2 | 6,65 | 1433,78 | 0,909 | 2 | 1 | nifr | 278 | FVQAGSEVSALLGR |
| 986 | -2,1 | 6,7 | 1433,78 | 0,324 | 2 | 1 | nifr | 278 | FVQAGSEVSALLGR |
| **3** | **-42** | **7,34** | **2** | **2** | **1** | **2** | **53,6** | **sp\|Q95AD6\|ATPB_WHIBI** | **EC 3.6.3.14; ATP synthase F1 sector subunit beta;** |
| spectrum | log(e) | log(I) | m+h | delta | z | zeta | pre | Start | Sequence |
| 1036 | -1,6 | 6,22 | 1262,66 | 0,629 | 2 | 1 | plgk | 40 | MPNNYNALVVK |
| 1030 | -1,3 | 6,17 | 1262,66 | -0,01 | 2 | 1 | plgk | 40 | MPNNYNALVVK |
| 1113 | -6,9 | 6,44 | 1955,02 | 0,504 | 2 | 1 | tlgr | 110 | IFNVLGEPVDNLGPVDTR |
| 1117 | -6,4 | 6,35 | 1955,02 | 0,3 | 2 | 1 | tlgr | 110 | IFNVLGEPVDNLGPVDTR |
| 1118 | -5 | 6,24 | 1955,02 | 1,063 | 2 | 1 | tlgr | 110 | IFNVLGEPVDNLGPVDTR |
| 1669 | -1,2 | 5,77 | 1471,86 | 0,89 | 2 | 1 | gvgk | 179 | TVLIMELINNIAK |
| 1679 | -1,2 | 6,04 | 1471,86 | 1,342 | 2 | 1 | gvgk | 179 | TVLIMELINNIAK |
| 1456 | -1,1 | 5,66 | 1487,85 | 1,285 | 2 | 1 | gvgk | 179 | TVLIMELINNIAK |
| 475 | -2,2 | 5,69 | 1617,8 | 1,76 | 2 | 1 | aesk | 232 | VALVYGQMNEPPGAR |
| 984 | -3,2 | 6,65 | 1433,78 | 0,909 | 2 | 1 | nifr | 278 | FVQAGSEVSALLGR |
| 986 | -2,1 | 6,7 | 1433,78 | 0,324 | 2 | 1 | nifr | 278 | FVQAGSEVSALLGR |
| **4** | **-38** | **7,22** | **12** | **21** | **5** | **8** | **55,3** | **sp\|A9LYH0\|ATPA_ACOAM** | **EC 3.6.3.14; ATP synthase F1 sector subunit alpha;** |
| spectrum | log(e) | log(I) | m+h | delta | z | zeta | pre | Start | Sequence |
| 793 | -3,9 | 6,18 | 1598,89 | 0,871 | 2 | 1 | revk | 26 | VVNTGTVLQVGDGIAR |
| 791 | -3,1 | 6,31 | 1598,89 | 0,478 | 2 | 1 | revk | 26 | VVNTGTVLQVGDGIAR |
| 707 | -1,3 | 5,74 | 1598,89 | 2,201 | 2 | 1 | revk | 26 | VVNTGTVLQVGDGIAR |
| 787 | -2,1 | 6,59 | 1416,79 | 0,717 | 2 | 1 | atgr | 95 | IAQIPVSEAYLGR |
| 652 | -2,7 | 6,55 | 1252,73 | 0,451 | 2 | 1 | sefr | 129 | LIESPAPGIISR |
| 659 | -1,6 | 6,18 | 1252,73 | 0,787 | 2 | 1 | sefr | 129 | LIESPAPGIISR |
| 498 | -1,4 | 5,83 | 1274,7 | -0,13 | 2 | 1 | qtgk | 177 | TAVATDTILNQK |
| 1062 | -1,3 | 6,46 | 1251,65 | 1,345 | 2 | 1 | sstk | 481 | TFTEEAEALLK |
| **5** | **-36** | **7,35** | **11** | **24** | **5** | **8** | **50,4** | **tr\|Q6L9Z6\|Q6L9Z6_9LILI** | **RuBisCO large subunit;** |
| spectrum | log(e) | log(I) | m+h | delta | z | zeta | pre | Start | Sequence |
| 560 | -1,6 | 6,98 | 1407,67 | 0,915 | 2 | 1 | kdyk | 15 | LTYYTPEYETK |
| 581 | -1 | 5,83 | 1407,67 | 1,601 | 2 | 1 | kdyk | 15 | LTYYTPEYETK |
| 471 | -2,1 | 6,15 | 1465,76 | 0,991 | 2 | 1 | ayik | 140 | TFQGPPHGIQVER |
| 1058 | -1,1 | 6,54 | 1261,65 | 0,969 | 2 | 1 | wrdr | 211 | FLFCAEALYK |
| 369 | -2,4 | 6,52 | 1116,6 | 0,077 | 2 | 1 | vanr | 415 | VALEACVQAR |
| 360 | -1,4 | 5,95 | 1116,6 | 0,272 | 2 | 1 | vanr | 415 | VALEACVQAR |
| 528 | -2,3 | 6,06 | 1230,63 | 1,746 | 2 | 1 | negr | 429 | DLATEGNEIIR |
| 658 | -1,2 | 6,31 | 1230,63 | 1,369 | 2 | 1 | negr | 429 | DLATEGNEIIR |
| **6** | **-28** | **7,13** | **2** | **2** | **1** | **1** | **53,6** | **sp\|P62626\|ATPB_AEGCO** | **EC 3.6.3.14; ATP synthase F1 sector subunit beta;** |
| spectrum | log(e) | log(I) | m+h | delta | z | zeta | pre | Start | sequence |
| 782 | -2,1 | 6,12 | 1191,63 | 0,404 | 2 | 1 | pihr | 135 | SAPAFIELDTK |
| 1669 | -1,2 | 5,77 | 1471,86 | 0,89 | 2 | 1 | gvgk | 179 | TVLIMELINNIAK |
| 1679 | -1,2 | 6,04 | 1471,86 | 1,342 | 2 | 1 | gvgk | 179 | TVLIMELINNIAK |
| 1456 | -1,1 | 5,66 | 1487,85 | 1,285 | 2 | 1 | gvgk | 179 | TVLIMELINNIAK |
| 475 | -2,2 | 5,69 | 1617,8 | 1,76 | 2 | 1 | eesk | 232 | VALVYGQMNEPPGAR |
| 984 | -3,2 | 6,65 | 1433,78 | 0,909 | 2 | 1 | nifr | 278 | FVQAGSEVSALLGR |
| 986 | -2,1 | 6,7 | 1433,78 | 0,324 | 2 | 1 | nifr | 278 | FVQAGSEVSALLGR |
| **7** | **-19** | **6,87** | **2,3** | **2** | **1** | **1** | **50,4** | **tr\|C6G4V9\|C6G4V9_9ASPA** | **Ribulose-1:p,5-bisphosphate carboxylase/oxygenase large subunit;** |
| spectrum | log(e) | log(I) | m+h | delta | z | zeta | pre | Start | Sequence |
| 471 | -2,1 | 6,15 | 1465,76 | 0,991 | 2 | 1 | sysk | 140 | TFQGPPHGIQVER |
| 1392 | -2,2 | 6,27 | 1292,7 | 2,441 | 2 | 1 | eger | 333 | QMTLGFVDLLR |
| 369 | -2,4 | 6,52 | 1116,6 | 0,077 | 2 | 1 | vanr | 415 | VALEACVQAR |
| 360 | -1,4 | 5,95 | 1116,6 | 0,272 | 2 | 1 | vanr | 415 | VALEACVQAR |
| **8** | **-19** | **6,18** | **6,3** | **8** | **3** | **3** | **59,1** | **sp\|P19023\|ATPBM_MAIZE** | **ATP synthase subunit beta, mitochondrial; EC 3.6.3.14;** |
| spectrum | log(e) | log(I) | m+h | delta | z | zeta | pre | Start | Sequence |
| 430 | -1,7 | 5,56 | 1278,63 | 2,452 | 2 | 1 | nmvr | 132 | TIAMDGTEGLVR |
| 1544 | -2,2 | 5,73 | 1457,84 | 1,993 | 2 | 1 | gvgk | 235 | TVLIMELINNVAK |
| 895 | -2,6 | 5,79 | 1399,77 | 0,159 | 2 | 1 | arar | 306 | VGLTGLTVAEHFR |
| **9** | **-9,3** | **5,93** | **4,6** | **6** | **2** | **2** | **61,4** | **tr\|Q7X9A7\|Q7X9A7_ORYSJ** | **60 kDa chaperonin alpha subunit;** |
| spectrum | log(e) | log(I) | m+h | delta | z | zeta | pre | Start | Sequence |
| 998 | -2 | 5,63 | 1555,91 | -0,73 | 2 | 1 | eiik | 142 | LGLLSVTSGANPVSIK |
| 697 | -2,3 | 5,63 | 1479,75 | 1,552 | 2 | 1 | eidr | 238 | GYISPQFVTNPEK |
| **10** | **-5,1** | **5,2** | **4,1** | **6** | **1** | **1** | **55,1** | **sp\|P05494\|ATPAM_MAIZE** | **ATP synthase subunit alpha, mitochondrial;** |
| spectrum | log(e) | log(I) | m+h | delta | z | zeta | pre | Start | Sequence |
| 1661 | -5,1 | 5,2 | 2308,16 | 1,114 | 2 | 1 | aqyr | 402 | EVAAFAQFGSDLDAATQALLNR |
| **1** | **-22** | **6,77** | **10** | **15** | **3** | **5** | **42,7** | **tr\|F2D714\|F2D714_HORVD** | **Predicted protein;** |
| spectrum | log(e) | log(I) | m+h | delta | z | zeta | pre | Start | Sequence |
| 969 | -4,6 | 6,38 | 1443,7 | 1,292 | 2 | 1 | hllk | 113 | YDSTLGIFDADVK |
| 977 | -1,2 | 5,91 | 1443,7 | -0,45 | 2 | 1 | hllk | 113 | YDSTLGIFDADVK |
| 1218 | -1,8 | 5,93 | 1780,02 | 0,478 | 2 | 1 | ialr | 300 | VPTPNVSVVDLVVQVSK |
| 998 | -3,6 | 6,04 | 1786,82 | 1,162 | 2 | 1 | dmvk | 375 | VIAWYDNEWGYSQR |
| 1008 | -1,7 | 5,85 | 1786,82 | 0,411 | 2 | 1 | dmvk | 375 | VIAWYDNEWGYSQR |
| **2** | **-22** | **6,67** | **7,9** | **9** | **3** | **4** | **50** | **tr\|Q1ENY9\|Q1ENY9_MUSAC** | **Phosphoglycerate kinase, chloroplast, putative; EC 2.7.2.3;** |
| spectrum | log(e) | log(I) | m+h | delta | z | zeta | pre | Start | Sequence |
| 785 | -3 | 6,16 | 1404,74 | 0,698 | 2 | 1 | llqk | 252 | ELDYLVGAVSNPK |
| 443 | -2,5 | 5,96 | 1102,64 | 1,237 | 2 | 1 | snpk | 265 | RPFAAIVGGSK |
| 664 | -4,2 | 6,3 | 1573,84 | 1,904 | 2 | 1 | lsgk | 423 | GVTTIIGGGDSVAAVEK |
| 644 | -1,9 | 5,55 | 1573,84 | 0,156 | 2 | 1 | lsgk | 423 | GVTTIIGGGDSVAAVEK |
| **3** | **-21** | **6,57** | **3,5** | **4** | **1** | **1** | **0** | **sp\|P12782\|PGKH_WHEAT** | **no protein information available** |
| spectrum | log(e) | log(I) | m+h | delta | z | zeta | pre | Start | Sequence |
| 668 | -2,6 | 5,7 | 2028,95 | 1,629 | 2 | 1 | vlvr | 95 | ADLNVPLDDNQNITDDTR |
| 443 | -2,5 | 5,96 | 1102,64 | 1,237 | 2 | 1 | snpk | 263 | RPFAAIVGGSK |
| 664 | -4,2 | 6,3 | 1573,84 | 1,904 | 2 | 1 | lskk | 421 | GVTTIIGGGDSVAAVEK |
| 644 | -1,9 | 5,55 | 1573,84 | 0,156 | 2 | 1 | lskk | 421 | GVTTIIGGGDSVAAVEK |
| **4** | **-20** | **6,65** | **1,9** | **2** | **1** | **1** | **0** | **tr\|B6STH5\|B6STH5_MAIZE** | **Phosphoglycerate kinase; EC 2.7.2.3;** |
| spectrum | log(e) | log(I) | m+h | delta | z | zeta | pre | Start | Sequence |
| 443 | -2,5 | 5,96 | 1102,64 | 1,237 | 2 | 1 | sspk | 266 | RPFAAIVGGSK |
| 345 | -1,4 | 6,08 | 992,542 | 1,542 | 2 | 1 | efdk | 406 | FAVGTEAVAK |
| 664 | -4,2 | 6,3 | 1573,84 | 1,904 | 2 | 1 | lsgk | 424 | GVTTIIGGGDSVAAVEK |
| 644 | -1,9 | 5,55 | 1573,84 | 0,156 | 2 | 1 | lsgk | 424 | GVTTIIGGGDSVAAVEK |
| **5** | **-18** | **6,32** | **7,8** | **10** | **3** | **3** | **53,6** | **sp\|Q3V527\|ATPB_ACOCL** | **EC 3.6.3.14; ATP synthase F1 sector subunit beta;** |
| spectrum | log(e) | log(I) | m+h | delta | z | zeta | pre | Start | Sequence |
| 1646 | -2,3 | 5,48 | 1471,86 | 0,231 | 2 | 1 | gvgk | 179 | TVLIMELINNIAK |
| 615 | -1,9 | 5,54 | 1601,81 | -0,06 | 2 | 1 | eesk | 232 | VALVYGQMNEPPGAR |
| 954 | -2,3 | 6,15 | 1433,78 | 0,699 | 2 | 1 | nifr | 278 | FVQAGSEVSALLGR |
| **6** | **-11** | **6,26** | **5,4** | **6** | **1** | **1** | **31,4** | **tr\|C1JYE2\|C1JYE2_9POAL** | **Phosphoglycerate kinase; EC 2.7.2.3;** |
| spectrum | log(e) | log(I) | m+h | delta | z | zeta | pre | Start | Sequence |
| 443 | -2,5 | 5,96 | 1102,64 | 1,237 | 2 | 1 | snpk | 118 | RPFAAIVGGSK |
| 660 | -3,2 | 5,96 | 1574,8 | 1,707 | 2 | 1 | lskk | 276 | GVTTNIGGGDSVAAVEK |
| **7** | **-7,4** | **6,07** | **5** | **12** | **2** | **2** | **48,9** | **tr\|O78641\|O78641_9ASPA** | **no protein information available** |
| spectrum | log(e) | log(I) | m+h | delta | z | zeta | pre | Start | Sequence |
| 438 | -1,4 | 5,68 | 1465,76 | 1,384 | 2 | 1 | sysk | 139 | TFQGPPHGIQVER |
| 1353 | -1,2 | 5,84 | 1295,66 | 2,341 | 2 | 1 | eger | 332 | DMTLGFVDLLR |
| **8** | **-4,7** | **5,7** | **4,2** | **6** | **1** | **1** | **47,2** | **sp\|Q42450\|RCAB_HORVU** | **no protein information available** |
| spectrum | log(e) | log(I) | m+h | delta | z | zeta | pre | Start | Sequence |
| 1322 | -4,7 | 5,7 | 2089,17 | 0,82 | 2 | 1 | enpr | 265 | VPIIVTGNDFSTLYAPLIR |
| **9** | **-3,7** | **6,35** | **3,3** | **5** | **1** | **2** | **39,8** | **sp\|P0C1M0\|ATPG_MAIZE** | **F-ATPase gamma subunit;** |
| spectrum | log(e) | log(I) | m+h | delta | z | zeta | pre | Start | Sequence |
| 783 | -3,7 | 5,88 | 1358,73 | -0,41 | 2 | 1 | qilr | 301 | ALQESLASELAAR |
| 776 | -1,7 | 6,17 | 1358,73 | 0,258 | 2 | 1 | qilr | 301 | ALQESLASELAAR |
| **10** | **-3,5** | **6,09** | **5,1** | **8** | **1** | **1** | **33** | **sp\|P27337\|PER1_HORVU** | **Peroxidase 1; EC 1.11.1.7;** |
| spectrum | log(e) | log(I) | m+h | delta | z | zeta | pre | Start | Sequence |
| 1054 | -3,5 | 6,09 | 1710,92 | 0,553 | 2 | 1 | vaar | 123 | DSVVALGGPSWTVPLGR |
| **11** | **-3,4** | **5,65** | **2,9** | **4** | **1** | **1** | **47,1** | **tr\|Q9SNK3\|Q9SNK3_ORYSJ** | **Glyceraldehyde-3-phosphate dehydrogenase B; Os03g0129300 protein;** |
| spectrum | log(e) | log(I) | m+h | delta | z | zeta | pre | Start | Sequence |
| 950 | -3,4 | 5,65 | 1772,8 | 0,985 | 2 | 1 | dmvk | 389 | VVAWYDNEWGYSQR |
| **12** | **-3,1** | **5,35** | **3,4** | **5** | **1** | **1** | **42** | **sp\|Q40677\|ALFC_ORYSJ** | **Fructose-bisphosphate aldolase, chloroplastic; ALDP; EC 4.1.2.13;** |
| spectrum | log(e) | log(I) | m+h | delta | z | zeta | pre | Start | Sequence |
| 292 | -3,1 | 5,35 | 1482,67 | 0,439 | 2 | 1 | spgr | 58 | GILAMDESNATCGK |
| **13** | **-2,7** | **5,7** | **4,7** | **6** | **1** | **1** | **35,2** | **sp\|A6MMM0\|CYF_DIOEL** | **Apocytochrome f;** |
| spectrum | log(e) | log(I) | m+h | delta | z | zeta | pre | Start | sequence |
| 448 | -2,7 | 5,7 | 1625,81 | -0,04 | 2 | 1 | dgsk | 201 | SNNTVYNATSAGIVSK |
| **15** | **-2,1** | **5,75** | **3,2** | **4** | **1** | **1** | **44,5** | **sp\|P37833\|AATC_ORYSJ** | **Aspartate aminotransferase, cytoplasmic; EC 2.6.1.1;** |
| spectrum | log(e) | log(I) | m+h | delta | z | zeta | pre | Start | sequence |
| 512 | -2,1 | 5,75 | 1448,77 | -0,85 | 2 | 1 | qenr | 101 | VATVQCLSGTGSLR |
| **19** | **-1,3** | **6,68** | **3,3** | **5** | **1** | **3** | **35,6** | **sp\|Q08062\|MDHC_MAIZE** | **Malate dehydrogenase, cytoplasmic; EC 1.1.1.37;** |
| spectrum | log(e) | log(I) | m+h | delta | z | zeta | pre | Start | sequence |
| 1319 | -1,3 | 5,9 | 1346,74 | 0,094 | 2 | 1 | ngvk | 56 | MELVDAAFPLLK |
| 1168 | -1,3 | 6,13 | 1362,73 | 0,175 | 2 | 1 | ngvk | 56 | MELVDAAFPLLK |
| 1307 | -1,1 | 6,42 | 1346,74 | 0,573 | 2 | 1 | ngvk | 56 | MELVDAAFPLLK |
| **1** | **-11** | **6,23** | **6,1** | **9** | **2** | **2** | **41** | **tr\|Q0DJC0\|Q0DJC0_ORYSJ** | **Os05g0302700 protein;** |
| spectrum | log(e) | log(I) | m+h | delta | z | zeta | pre | Start | sequence |
| 1107 | -2,6 | 5,86 | 1446,74 | 0,124 | 2 | 1 | nvir | 154 | YFPTQALNFAFK |
| 491 | -3,6 | 5,98 | 1191,64 | 0,394 | 2 | 1 | nilr | 353 | AVAGAGVLAGYDK |
| **2** | **-8,2** | **6,2** | **5** | **12** | **2** | **2** | **49,2** | **tr\|Q8WL39\|Q8WL39_9ASPA** | **Ribulose-bisphosphate carboxylase large subunit;** |
| spectrum | log(e) | log(I) | m+h | delta | z | zeta | pre | Start | sequence |
| 470 | -1,4 | 6,14 | 1407,67 | 0,581 | 2 | 1 | kdyr | 15 | LTYYTPEYETK |
| 362 | -1,9 | 5,27 | 1466,74 | 2,189 | 2 | 1 | aysk | 140 | TFEGPPHGIQVER |
| **3** | **-2,5** | **5,54** | **3** | **3** | **1** | **1** | **42,3** | **tr\|Q1EPF8\|Q1EPF8_MUSAC** | **Phosphoglycerate kinase 2; EC 2.7.2.3;** |
| spectrum | log(e) | log(I) | m+h | delta | z | zeta | pre | Start | sequence |
| 728 | -2,5 | 5,54 | 1404,74 | 0,78 | 2 | 1 | lmqk | 179 | ELDYLVGAVSNPK |
| **4** | **-2,5** | **5,66** | **3,9** | **6** | **1** | **1** | **29,6** | **sp\|Q6L5I5\|VDAC2_ORYSJ** | **Voltage-dependent anion-selective channel protein 2; OsVDAC2** |
| spectrum | log(e) | log(I) | m+h | delta | z | zeta | pre | Start | sequence |
| 725 | -2,5 | 5,66 | 1292,7 | 0,437 | 2 | 1 | frpk | 253 | SLVTISTEVDTK |
| **5** | **-2,4** | **5,37** | **2** | **2** | **1** | **1** | **63,8** | **tr\|Q6ZFJ9\|Q6ZFJ9_ORYSJ** | **60 kDa chaperonin beta subunit; Os02g0102900 protein;** |
| spectrum | log(e) | log(I) | m+h | delta | z | zeta | pre | Start | sequence |
| 323 | -2,4 | 5,37 | 1295,74 | -0,02 | 2 | 1 | egvk | 158 | VVAAGANPVQITR |
| **6** | **-2** | **6,13** | **4,9** | **6** | **1** | **2** | **34,4** | **tr\|F2CRK1\|F2CRK1_HORVD** | **Predicted protein;** |
| spectrum | log(e) | log(I) | m+h | delta | z | zeta | pre | Start | sequence |
| 392 | -2 | 5,89 | 1562,76 | 1,227 | 2 | 1 | pkgr | 242 | GGSTGYDNAVALPAGGR |
| 400 | -1,3 | 5,76 | 1562,76 | 1,103 | 2 | 1 | pkgr | 242 | GGSTGYDNAVALPAGGR |
| **9** | **-1,5** | **5,78** | **5,7** | **8** | **1** | **2** | **29,8** | **tr\|G0YLW6\|G0YLW6_9ARAE** | **Putative chlorophyll a/b binding protein;** |
| spectrum | log(e) | log(I) | m+h | delta | z | zeta | pre | Start | sequence |
| 1386 | -1,5 | 5,55 | 1835,95 | 1,07 | 2 | 1 | ihar | 119 | WAMLGAAGFIIPEAFNK |
| 1378 | -1,2 | 5,4 | 1835,95 | 0,957 | 2 | 1 | ihar | 119 | WAMLGAAGFIIPEAFNK |
| **1** | **-23** | **6,71** | **15** | **25** | **3** | **10** | **27,7** | **tr\|Q6WFB1\|Q6WFB1_MAIZE** | **Photosystem II subunit PsbS;** |
| spectrum | log(e) | log(I) | m+h | delta | z | zeta | pre | Start | sequence |
| 1006 | -4,4 | 5,65 | 1584,79 | 0,427 | 2 | 1 | pkpk | 73 | VEDGIFGTSGGIGFTK |
| 1009 | -2,2 | 5,61 | 1584,79 | 1,345 | 2 | 1 | pkpk | 73 | VEDGIFGTSGGIGFTK |
| 991 | -1,3 | 5,43 | 1584,79 | 0,117 | 2 | 1 | pkpk | 73 | VEDGIFGTSGGIGFTK |
| 1835 | -5 | 5,58 | 1748,96 | 1,991 | 2 | 1 | fvgr | 97 | VAMLGFAASLLGEAITGK |
| 1500 | -4,5 | 5,9 | 1764,95 | 1,692 | 2 | 1 | fvgr | 97 | VAMLGFAASLLGEAITGK |
| 1516 | -4,4 | 5,26 | 1764,95 | 1,257 | 2 | 1 | fvgr | 97 | VAMLGFAASLLGEAITGK |
| 1837 | -4 | 5,48 | 1748,96 | 0,934 | 2 | 1 | fvgr | 97 | VAMLGFAASLLGEAITGK |
| 1819 | -3,5 | 5,25 | 1748,96 | 1,322 | 2 | 1 | fvgr | 97 | VAMLGFAASLLGEAITGK |
| 1491 | -3 | 5,38 | 1764,95 | 2,188 | 2 | 1 | fvgr | 97 | VAMLGFAASLLGEAITGK |
| 922 | -1,1 | 6,29 | 1052,54 | 1,075 | 2 | 1 | lgls | 183 | EGGPLFGFTK |
| **2** | **-4** | **5,5** | **6,8** | **12** | **1** | **1** | **21,9** | **sp\|P36213\|PSAD_HORVU** | **Photosystem I 20 kDa subunit;** |
| spectrum | log(e) | log(I) | m+h | delta | z | zeta | pre | Start | Sequence |
| 542 | -4 | 5,5 | 1668,76 | -0,13 | 2 | 1 | tspk | 104 | EQVFEMPTGGAAIMR |
| **7** | **-1,4** | **5,66** | **4,3** | **5** | **1** | **1** | **33,2** | **sp\|P08477\|G3PC_HORVU** | **Glyceraldehyde-3-phosphate dehydrogenase, cytosolic; EC 1.2.1.12;** |
| spectrum | log(e) | log(I) | m+h | delta | z | zeta | pre | Start | Sequence |
| 1023 | -1,4 | 5,66 | 1498,85 | 0,647 | 2 | 1 | mafr | 205 | VPTVDVSVVDLTVR |
| **1** | **-1,9** | **5,69** | **13** | **14** | **1** | **1** | **9,3** | **tr\|I1IWU7\|I1IWU7_BRADI** | **Uncharacterized protein;** |
| spectrum | log(e) | log(I) | m+h | delta | z | zeta | pre | Start | Sequence |
| 982 | -1,9 | 5,69 | 1484,7 | 1,58 | 2 | 1 | itdr | 69 | FNSLEQLDEFSR |
| **2** | **-1,8** | **5,75** | **13** | **28** | **1** | **1** | **9,4** | **sp\|A1EA25\|PSBE_AGRST** | **no protein information available** |
| spectrum | log(e) | log(I) | m+h | delta | z | zeta | pre | Start | Sequence |
| 981 | -1,8 | 5,75 | 1485,69 | 0,71 | 2 | 1 | itdr | 70 | FDSLEQLDEFSR |

| **B** |  |  |  |  |  |  |  |  |  |
| --- | --- | --- | --- | --- | --- | --- | --- | --- | --- |
| **rank** | **log (e)** | **log (I)** | **% (m)** | **% (c)** | **unique** | **tot** | **Mr** | **Accession** | **Description** |
| **1** | **-37** | **6,8** | **11** | **24** | **5** | **8** | **50,4** | **tr\|Q6L9Z6\|Q6L9Z6_9LILI** | **RuBisCO large subunit;** |
| spectrum | log(e) | log(I) | m+h | delta | z | zeta | pre | Start | Sequence |
| 578 | -2 | 6,06 | 1407,7 | 0,072 | 2 | 1 | kdyk | 15 | LTYYTPEYETK |
| 471 | -2 | 5,54 | 1465,8 | 0,223 | 2 | 0,667 | ayik | 140 | TFQGPPHGIQVER |
| 486 | -2 | 5,34 | 1465,8 | 0,71 | 2 | 0,667 | ayik | 140 | TFQGPPHGIQVER |
| 1063 | -1 | 6,06 | 1261,6 | 0,195 | 2 | 1 | wrdr | 211 | FLFCAEALYK |
| 368 | -2 | 6,08 | 1116,6 | 0,633 | 2 | 1 | vanr | 415 | VALEACVQAR |
| 370 | -2 | 6,11 | 1116,6 | 0,973 | 2 | 1 | vanr | 415 | VALEACVQAR |
| 532 | -2 | 5,77 | 1230,6 | 0,603 | 2 | 1 | negr | 429 | DLATEGNEIIR |
| 523 | -2 | 5,63 | 1230,6 | 2,142 | 2 | 1 | negr | 429 | DLATEGNEIIR |
| **2** | **-3** | **5,24** | **2,6** | **7** | **1** | **1** | **56** | **sp\|A9LYC6\|PSBB_ACOAM** | **Photosystem II CP47 chlorophyll apoprotein;** |
| spectrum | log(e) | log(I) | m+h | delta | z | zeta | pre | Start | Sequence |
| 1028 | -3 | 5,24 | 1923,9 | -0,712 | 2 | 1 | gptr | 273 | YQWDQGYFQQEIYR |
| **3** | **-2** | **5,36** | **2** | **3** | **1** | **1** | **71,5** | **tr\|C5YWM8\|C5YWM8_SORBI** | **no protein information available** |
| spectrum | log(e) | log(I) | m+h | delta | z | zeta | pre | start | Sequence |
| 786 | -2 | 5,36 | 1287,6 | 0,559 | 2 | 1 | sssk | 335 | DISAAAAAGAGGAER |
| **4** | **-2** | **5,73** | **4,3** | **5** | **1** | **1** | **33,2** | **sp\|P08477\|G3PC_HORVU** | **Glyceraldehyde-3-phosphate dehydrogenase, cytosolic; EC 1.2.1.12;** |
| spectrum | log(e) | log(I) | m+h | delta | z | zeta | pre | start | Sequence |
| 1016 | -2 | 5,73 | 1498,8 | 1,516 | 2 | 1 | mafr | 205 | VPTVDVSVVDLTVR |
| **5** | **-2** | **5,69** | **3** | **5** | **1** | **1** | **55,3** | **sp\|A9LYH0\|ATPA_ACOAM** | **EC 3.6.3.14; ATP synthase F1 sector subunit alpha;** |
| spectrum | log(e) | log(I) | m+h | delta | z | zeta | pre | start | Sequence |
| 798 | -2 | 5,69 | 1598,9 | 0,594 | 2 | 1 | revk | 26 | VVNTGTVLQVGDGIAR |
| **1** | **-46** | **6,83** | **14** | **17** | **5** | **9** | **53,6** | **tr\|H2CPP4\|H2CPP4_COLES** | **EC 3.6.3.14; ATP synthase F1 sector subunit beta;** |
| spectrum | log(e) | log(I) | m+h | delta | z | zeta | pre | start | Sequence |
| 1254 | -5 | 5,73 | 1735 | -0,001 | 2 | 1 | nlgr | 23 | IAQIIGPVLDVAFPPGK |
| 1288 | -4 | 5,49 | 1735 | 0,627 | 2 | 1 | nlgr | 23 | IAQIIGPVLDVAFPPGK |
| 1072 | -5 | 5,59 | 1955 | 0,53 | 2 | 1 | tlgr | 110 | IFNVLGEPVDNLGPVDTR |
| 1078 | -4 | 5,48 | 1955 | 2,437 | 2 | 1 | tlgr | 110 | IFNVLGEPVDNLGPVDTR |
| 390 | -3 | 5,9 | 1517,7 | 1,701 | 2 | 1 | memk | 218 | ESGVINEQNIAESK |
| 385 | -1 | 5,93 | 1517,7 | 1,203 | 2 | 1 | memk | 218 | ESGVINEQNIAESK |
| 1040 | -2 | 5,54 | 1487,8 | 0,891 | 2 | 1 | armr | 249 | VGLTALTMAEYFR |
| 1275 | -2 | 6,39 | 1471,8 | 0,774 | 2 | 1 | armr | 249 | VGLTALTMAEYFR |
| 972 | -3 | 5,94 | 1433,8 | 1,092 | 2 | 1 | nifr | 278 | FVQAGSEVSALLGR |
| **2** | **-43** | **6,79** | **5** | **5** | **1** | **1** | **59,1** | **tr\|H6THB0\|H6THB0_9LILI** | **ATP synthase subunit beta; EC 3.6.3.14;** |
| spectrum | log(e) | log(I) | m+h | delta | z | zeta | pre | start | Sequence |
| 840 | -2 | 5,38 | 2099,1 | -0,614 | 2 | 1 | gltr | 18 | GMEVVDTGAPLSVPVGGATLGR |
| 1072 | -5 | 5,59 | 1955 | 0,53 | 2 | 1 | tlgr | 40 | IFNVLGEPVDNLGPVDTR |
| 1078 | -4 | 5,48 | 1955 | 2,437 | 2 | 1 | tlgr | 40 | IFNVLGEPVDNLGPVDTR |
| 390 | -3 | 5,9 | 1517,7 | 1,701 | 2 | 1 | memk | 148 | ESGVINEQNIAESK |
| 385 | -1 | 5,93 | 1517,7 | 1,203 | 2 | 1 | memk | 148 | ESGVINEQNIAESK |
| 1040 | -2 | 5,54 | 1487,8 | 0,891 | 2 | 1 | armr | 179 | VGLTALTMAEYFR |
| 1275 | -2 | 6,39 | 1471,8 | 0,774 | 2 | 1 | armr | 179 | VGLTALTMAEYFR |
| 972 | -3 | 5,94 | 1433,8 | 1,092 | 2 | 1 | nifr | 208 | FVQAGSEVSALLGR |
| **3** | **-38** | **6,79** | **7** | **16** | **5** | **9** | **49** | **tr\|B5RHG8\|B5RHG8_9ASPA** | **Ribulose-bisphosphate carboxylase large subunit;** |
| spectrum | log(e) | log(I) | m+h | delta | z | zeta | pre | start | Sequence |
| 478 | -4 | 5,58 | 1466,7 | -0,371 | 2 | 0,667 | aysk | 143 | TFEGPPHGIQVER |
| 464 | -4 | 5,76 | 1466,7 | -0,702 | 2 | 0,667 | aysk | 143 | TFEGPPHGIQVER |
| 476 | -1 | 5,6 | 1466,7 | 0,175 | 2 | 0,667 | aysk | 143 | TFEGPPHGIQVER |
| 468 | -1 | 5,39 | 1365,7 | -0,245 | 2 | 0,667 | yskt | 144 | FEGPPHGIQVER |
| 465 | -2 | 5,39 | 1200,6 | 0,567 | 2 | 0,667 | sktf | 145 | EGPPHGIQVER |
| 471 | -1 | 5,81 | 1218,6 | -0,624 | 2 | 0,667 | sktf | 145 | EGPPHGIQVER |
| 461 | -1 | 5,8 | 1218,6 | 0,854 | 2 | 0,667 | sktf | 145 | EGPPHGIQVER |
| 1362 | -1 | 6,23 | 1293,7 | 1,3 | 2 | 1 | eger | 336 | EMTLGFVDLLR |
| 370 | -2 | 6,14 | 1116,6 | 0,99 | 2 | 1 | vanr | 418 | VALEACVQAR |
| **4** | **-32** | **6,81** | **9,7** | **18** | **4** | **6** | **55,3** | **sp\|A9LYH0\|ATPA_ACOAM** | **EC 3.6.3.14; ATP synthase F1 sector subunit alpha;** |
| spectrum | log(e) | log(I) | m+h | delta | z | zeta | pre | Start | Sequence |
| 778 | -5 | 6,08 | 1598,9 | -0,195 | 2 | 1 | revk | 26 | VVNTGTVLQVGDGIAR |
| 782 | -4 | 6,09 | 1598,9 | 1,189 | 2 | 1 | revk | 26 | VVNTGTVLQVGDGIAR |
| 785 | -5 | 6,07 | 1416,8 | 0,816 | 2 | 1 | atgr | 95 | IAQIPVSEAYLGR |
| 776 | -3 | 6,13 | 1416,8 | 0,917 | 2 | 1 | atgr | 95 | IAQIPVSEAYLGR |
| 655 | -2 | 5,91 | 1252,7 | -0,159 | 2 | 1 | sefr | 129 | LIESPAPGIISR |
| 486 | -1 | 5,85 | 1274,7 | -0,427 | 2 | 1 | qtgk | 177 | TAVATDTILNQK |
| **5** | **-29** | **6,74** | **4,7** | **11** | **2** | **2** | **51,6** | **tr\|B0B735\|B0B735_9POAL** | **Ribulose-1:p,5-bisphosphate carboxylase/oxygenase large subunit; EC 4.1.1.39;** |
| spectrum | log(e) | log(I) | m+h | delta | z | zeta | pre | Start | Sequence |
| 466 | -4 | 5,81 | 1465,8 | 1,327 | 2 | 0,667 | tysk | 138 | TFQGPPHGIQVER |
| 370 | -2 | 6,14 | 1116,6 | 0,99 | 2 | 1 | aanr | 413 | VALEACVQAR |
| **6** | **-29** | **6,64** | **2,2** | **2** | **1** | **1** | **55,3** | **tr\|F8RS97\|F8RS97_JUNEF** | **ATP synthase subunit alpha** |
| spectrum | log(e) | log(I) | m+h | delta | z | zeta | pre | Start | Sequence |
| 785 | -5 | 6,07 | 1416,8 | 0,816 | 2 | 1 | atgr | 95 | IAQIPVSEAYLGR |
| 776 | -3 | 6,13 | 1416,8 | 0,917 | 2 | 1 | atgr | 95 | IAQIPVSEAYLGR |
| 452 | -2 | 5,43 | 1266,7 | 0,525 | 2 | 1 | ylgr | 108 | VINALAQPIDGR |
| 655 | -2 | 5,91 | 1252,7 | -0,159 | 2 | 1 | sesr | 129 | LIESPAPGIISR |
| 486 | -1 | 5,85 | 1274,7 | -0,427 | 2 | 1 | qtgk | 177 | TAVATDTILNQK |
| **7** | **-29** | **7,05** | **2,2** | **2** | **1** | **3** | **50,4** | **tr\|Q6L9Z6\|Q6L9Z6_9LILI** | **RuBisCO large subunit;** |
| spectrum | log(e) | log(I) | m+h | delta | z | zeta | pre | Start | Sequence |
| 466 | -4 | 5,81 | 1465,8 | 1,327 | 2 | 0,667 | ayik | 140 | TFQGPPHGIQVER |
| 370 | -2 | 6,14 | 1116,6 | 0,99 | 2 | 1 | vanr | 415 | VALEACVQAR |
| 650 | -2 | 6,64 | 1230,6 | 0,109 | 2 | 1 | negr | 429 | DLATEGNEIIR |
| 657 | -1 | 6,03 | 1230,6 | 1,676 | 2 | 1 | negr | 429 | DLATEGNEIIR |
| 652 | -1 | 6,55 | 1230,6 | 0,621 | 2 | 1 | negr | 429 | DLATEGNEIIR |
| **8** | **-20** | **6,69** | **2** | **2** | **1** | **1** | **55,3** | **sp\|P62626\|ATPB_AEGCO** | **EC 3.6.3.14; ATP synthase F1 sector subunit beta;** |
| spectrum | log(e) | log(I) | m+h | delta | z | zeta | pre | Start | Sequence |
| 774 | -2 | 6,08 | 1191,6 | 1,017 | 2 | 1 | pihr | 135 | SAPAFIELDTK |
| 1040 | -2 | 5,54 | 1487,8 | 0,891 | 2 | 1 | armr | 249 | VGLTALTMAEYFR |
| 1275 | -2 | 6,39 | 1471,8 | 0,774 | 2 | 1 | armr | 249 | VGLTALTMAEYFR |
| 972 | -3 | 5,94 | 1433,8 | 1,092 | 2 | 1 | nifr | 278 | FVQAGSEVSALLGR |
| **9** | **-18** | **6,24** | **7,4** | **9** | **3** | **4** | **59,1** | **sp\|P19023\|ATPBM_MAIZE** | **ATP synthase subunit beta, mitochondrial; EC 3.6.3.14;** |
| spectrum | log(e) | log(I) | m+h | delta | z | zeta | pre | start | Sequence |
| 639 | -1 | 5,7 | 1262,6 | 1,181 | 2 | 1 | nmvr | 132 | TIAMDGTEGLVR |
| 1522 | -3 | 5,7 | 1457,8 | 0,751 | 2 | 1 | gvgk | 235 | TVLIMELINNVAK |
| 1525 | -2 | 5,43 | 1457,8 | 1,93 | 2 | 1 | gvgk | 235 | TVLIMELINNVAK |
| 1044 | -2 | 5,67 | 2061,1 | 1,166 | 2 | 1 | vlsr | 412 | QISELGIYPAVDPLDSTSR |
| **10** | **-6** | **5,94** | **2** | **2** | **1** | **2** | **63,8** | **tr\|Q6ZFJ9\|Q6ZFJ9_ORYSJ** | **60 kDa chaperonin beta subunit; Os02g0102900 protein;** |
| spectrum | log(e) | log(I) | m+h | delta | z | zeta | pre | start | Sequence |
| 409 | -6 | 5,75 | 1295,7 | -0,556 | 2 | 1 | egvk | 158 | VVAAGANPVQITR |
| 419 | -3 | 5,5 | 1295,7 | -0,644 | 2 | 1 | egvk | 158 | VVAAGANPVQITR |
| **1** | **-22** | **6,45** | **7,9** | **9** | **3** | **3** | **50** | **tr\|Q1ENY9\|Q1ENY9_MUSAC** | **Phosphoglycerate kinase, chloroplast, putative; EC 2.7.2.3;** |
| spectrum | log(e) | log(I) | m+h | delta | z | zeta | pre | start | Sequence |
| 782 | -3 | 5,98 | 1404,7 | 0,667 | 2 | 1 | llqk | 252 | ELDYLVGAVSNPK |
| 429 | -2 | 5,68 | 1102,6 | 0,027 | 2 | 0,667 | snpk | 265 | RPFAAIVGGSK |
| 651 | -4 | 6,14 | 1573,8 | 0,624 | 2 | 1 | lsgk | 423 | GVTTIIGGGDSVAAVEK |
| **2** | **-20** | **6,35** | **3,3** | **3** | **1** | **1** | **31,4** | **tr\|B6STH5\|B6STH5_MAIZE** | **Phosphoglycerate kinase; EC 2.7.2.3;** |
| spectrum | log(e) | log(I) | m+h | delta | z | zeta | pre | start | Sequence |
| 1183 | -2 | 5,57 | 1748 | -0,281 | 2 | 1 | evek | 180 | LVAALPNGGVLLLENVR |
| 429 | -2 | 5,68 | 1102,6 | 0,027 | 2 | 0,667 | sspk | 266 | RPFAAIVGGSK |
| 651 | -4 | 6,14 | 1573,8 | 0,624 | 2 | 1 | lsgk | 424 | GVTTIIGGGDSVAAVEK |
| **3** | **-10** | **6,57** | **14** | **19** | **2** | **3** | **20,8** | **tr\|F8UCA0\|F8UCA0_9LILI** | **Glyceraldehyde-3-phosphate dehydrogenase; EC 1.2.1.12;** |
| spectrum | log(e) | log(I) | m+h | delta | z | zeta | pre | start | Sequence |
| 867 | -2 | 5,37 | 1743,8 | 0,134 | 2 | 1 | apsk | 29 | DAPMFVMGVNEDQYK |
| 947 | -3 | 6,35 | 1498,8 | 1,217 | 2 | 1 | mafr | 137 | VPTVDVSVVDLTVR |
| 953 | -3 | 6,1 | 1498,8 | 1,326 | 2 | 1 | mafr | 137 | VPTVDVSVVDLTVR |
| **4** | **-9** | **6,53** | **6,7** | **10** | **2** | **2** | **42,7** | **tr\|F2D714\|F2D714_HORVD** | **Predicted protein;** |
| spectrum | log(e) | log(I) | m+h | delta | z | zeta | pre | start | Sequence |
| 545 | -2 | 6,48 | 1384,8 | 0,63 | 2 | 1 | rrar | 266 | AAALNIVPTSTGAAK |
| 971 | -2 | 5,62 | 1786,8 | 0,21 | 2 | 1 | dmvk | 375 | VIAWYDNEWGYSQR |
| **5** | **-3** | **5,38** | **5,3** | **6** | **1** | **1** | **31,5** | **tr\|G3FBL3\|G3FBL3_9LILI** | **Actin; Flags: Fragment** |
| spectrum | log(e) | log(I) | m+h | delta | z | zeta | pre | start | Sequence |
| 903 | -3 | 5,38 | 1761,9 | -0,402 | 2 | 1 | siek | 147 | TYELPDGQVITIGAER |
| **7** | **-2** | **5,59** | **5,1** | **6** | **1** | **1** | **37** | **tr\|Q7XZW5\|Q7XZW5_ORYSJ** | **Malate dehydrogenase; EC 1.1.1.37** |
| spectrum | log(e) | log(I) | m+h | delta | z | zeta | pre | Start | Sequence |
| 1121 | -2 | 5,59 | 1795,1 | 0,244 | 2 | 1 | pgfk | 45 | VAVLGAAGGIGQPLSLLMK |
| **12** | **-1** | **5,77** | **3,3** | **5** | **1** | **1** | **39,8** | **sp\|P0C1M0\|ATPG_MAIZE** | **F-ATPase gamma subunit;** |
| spectrum | log(e) | log(I) | m+h | delta | z | zeta | pre | Start | sequence |
| 761 | -1 | 5,77 | 1358,7 | 1,643 | 2 | 1 | qilr | 301 | ALQESLASELAAR |
| **1** | **-4** | **5,48** | **4,3** | **6** | **1** | **1** | **26,7** | **sp\|P34937\|TPIS_HORVU** | **Triose-phosphate isomerase; EC 5.3.1.1** |
| spectrum | log(e) | log(I) | m+h | delta | z | zeta | pre | Start | sequence |
| 483 | -4 | 5,48 | 1374,7 | -0,362 | 2 | 1 | qglk | 124 | VIACVGETLEQR |
| **2** | **-2** | **5,81** | **4,9** | **6** | **1** | **2** | **34,4** | **tr\|F2CRK1\|F2CRK1_HORVD** | **Predicted protein;** |
| spectrum | log(e) | log(I) | m+h | delta | z | zeta | pre | Start | sequence |
| 401 | -2 | 5,37 | 1562,8 | 0,425 | 2 | 1 | pkgr | 242 | GGSTGYDNAVALPAGGR |
| 387 | -2 | 5,61 | 1562,8 | -0,101 | 2 | 1 | pkgr | 242 | GGSTGYDNAVALPAGGR |
| **3** | **-2** | **5,23** | **5,2** | **6** | **1** | **1** | **26** | **tr\|Q6YTY2\|Q6YTY2_ORYSJ** | **Os07g0608500 protein; Putative 40S ribosomal protein;** |
| spectrum | log(e) | log(I) | m+h | delta | z | zeta | pre | Start | sequence |
| 417 | -2 | 5,23 | 1423,7 | 0,045 | 2 | 1 | mltr | 30 | ELAEDGYSGVEVR |
| **4** | **-2** | **5,55** | **2,8** | **4** | **1** | **1** | **42,4** | **sp\|P04709\|ADT1_MAIZE** | **ADP:p ,ATP carrier protein 1, mitochondrial; ADP/ATP translocase 1;** |
| spectrum | log(e) | log(I) | m+h | delta | z | zeta | pre | Start | sequence |
| 1102 | -2 | 5,55 | 1446,7 | 0,64 | 2 | 1 | nvir | 161 | YFPTQALNFAFK |
| **1** | **-4** | **5,76** | **3,8** | **6** | **1** | **1** | **24,8** | **sp\|P13192\|PSAF_HORVU** | **Light-harvesting complex I 17 kDa protein;** |
| spectrum | log(e) | log(I) | m+h | delta | z | zeta | pre | Start | sequence |
| 1178 | -4 | 5,76 | 1179,6 | 0,317 | 2 | 1 | iipr | 210 | GFIWPVAAYR |
| **1** | **-2** | **5,68** | **13** | **28** | **1** | **2** | **9,4** | **sp\|A1EA25\|PSBE_AGRST** | **Cytochrome b559 subunit alpha; PSII reaction center subunit V;** |
| spectrum | log(e) | log(I) | m+h | delta | z | zeta | pre | Start | sequence |
| 1012 | -2 | 5,35 | 1485,7 | 0,412 | 2 | 1 | itdr | 70 | FDSLEQLDEFSR |
| 1006 | -1 | 5,41 | 1485,7 | 1,349 | 2 | 1 | itdr | 70 | FDSLEQLDEFSR |

| **C** |  |  |  |  |  |  |  |  |  |  |  |  |  |  |  |  |
| --- | --- | --- | --- | --- | --- | --- | --- | --- | --- | --- | --- | --- | --- | --- | --- | --- |
| **Rank** | **log (e)** | **log (I)** | **% (m)** | **% (c)** | **unique** | **tot** | **Mr** | **Accession** | **Description** |  |  |  |  |  |  |  |
| **1** | **-18** | **6,28** | **8,2** | **12** | **3** | **3** | **41,7** | **tr\|C7IWD0\|C7IWD0_ORYSJ** | **Os01g0791600 protein;** |  |  |  |  |  |  |  |
| spectrum | log(e) | log(I) | m+h | delta | z | zeta | pre | Start | Sequence |  |  |  |  |  |  |  |
| 617 | -1,6 | 6,03 | 1408 | 1 | 2 | 1 | kdyk | 22 | LTYYTPEYETK |  |  |  |  |  |  |  |
| 787 | -1,4 | 5,65 | 1228 | 0,2 | 2 | 1 | lnlr | 259 | AYDFVSQEIR |  |  |  |  |  |  |  |
| 853 | -2,9 | 5,58 | 1548 | 1,5 | 2 | 1 | qeir | 269 | AAEDPEFETFYTK |  |  |  |  |  |  |  |
| **2** | **-9,3** | **6,15** | **4,3** | **8** | **2** | **3** | **55,3** | **sp\|A9LYH0\|ATPA_ACOAM** | **EC 3.6.3.14; ATP synthase F1 sector subunit alpha;** |  |  |  |  |  |  |  |
| spectrum | log(e) | log(I) | m+h | delta | z | zeta | pre | Start | sequence |  |  |  |  |  |  |  |
| 698 | -2,5 | 5,79 | 1253 | 0,4 | 2 | 1 | sefr | 129 | LIESPAPGIISR |  |  |  |  |  |  |  |
| 710 | -1,4 | 5,69 | 1253 | 2,2 | 2 | 1 | sefr | 129 | LIESPAPGIISR |  |  |  |  |  |  |  |
| 541 | -1,9 | 5,47 | 1275 | 0,1 | 2 | 1 | qtgk | 177 | TAVATDTILNQK |  |  |  |  |  |  |  |
| **3** | **-7,7** | **6,41** | **2,2** | **2** | **1** | **2** | **50,4** | **tr\|Q6L9Z6\|Q6L9Z6_9LILI** | **RuBisCO large subunit;** |  |  |  |  |  |  |  |
| spectrum | log(e) | log(I) | m+h | delta | z | zeta | pre | Start | sequence |  |  |  |  |  |  |  |
| 617 | -1,6 | 6,03 | 1408 | 1 | 2 | 1 | kdyk | 15 | LTYYTPEYETK |  |  |  |  |  |  |  |
| 692 | -1,2 | 6,07 | 1231 | 0 | 2 | 1 | negr | 429 | DLATEGNEIIR |  |  |  |  |  |  |  |
| 575 | -1,1 | 5,54 | 1231 | -0,1 | 2 | 1 | negr | 429 | DLATEGNEIIR |  |  |  |  |  |  |  |
| **4** | **-4,3** | **5,41** | **3,8** | **4** | **1** | **1** | **48,1** | **sp\|P42895\|ENO2_MAIZE** | **no protein information available** |  |  |  |  |  |  |  |
| Spectrum | log(e) | log(I) | m+h | delta | z | zeta | pre | Start | sequence |  |  |  |  |  |  |  |
| 1018 | -4,3 | 5,41 | 1791 | -0,4 | 2 | 1 | tfar | 36 | AAVPSGASTGVYEALELR |  |  |  |  |  |  |  |
| **6** | **-2,4** | **5,48** | **1,5** | **2** | **1** | **1** | **105** | **sp\|Q7XPY2\|PMA1_ORYSJ** | **no protein information available** |  |  |  |  |  |  |  |
| Spectrum | log(e) | log(I) | m+h | delta | z | zeta | pre | Start | sequence |  |  |  |  |  |  |  |
| 821 | -2,4 | 5,48 | 1430 | -0,6 | 2 | 1 | alkk | 599 | ADIGIAVADATDAAR |  |  |  |  |  |  |  |
| **7** | **-2,2** | **5,53** | **3,5** | **11** | **1** | **1** | **50,8** | **tr\|G1C6J9\|G1C6J9_9LILI** | **no protein information available** |  |  |  |  |  |  |  |
| Spectrum | log(e) | log(I) | m+h | delta | z | zeta | pre | Start | sequence |  |  |  |  |  |  |  |
| 1055 | -2,2 | 5,53 | 1762 | 2,1 | 2 | 1 | iyrr | 278 | VSAGLAENLSLSEAWSK |  |  |  |  |  |  |  |
| **10** | **-1,8** | **5,46** | **1,3** | **2** | **1** | **1** | **79,5** | **sp\|Q06572\|AVP_HORVU** | **EC 3.6.1.1; Pyrophosphate-energized inorganic pyrophosphatase;** |  |  |  |  |  |  |  |
| spectrum | log(e) | log(I) | m+h | delta | z | zeta | pre | Start | sequence |  |  |  |  |  |  |  |
| 412 | -1,8 | 5,46 | 1016 | 0,5 | 2 | 1 | iytk | 247 | AADVGADLVGK |  |  |  |  |  |  |  |
| **1** | **-54** | **6,94** | **18** | **22** | **6** | **10** | **53,6** | **sp\|A9L9A3\|ATPB_LEMMI** | **ATP synthase subunit beta, chloroplastic; EC 3.6.3.14;** |  |  |  |  |  |  |  |
| spectrum | log(e) | log(I) | m+h | delta | z | zeta | pre | Start | sequence |  |  |  |  |  |  |  |
| 899 | -4,6 | 5,55 | 2083 | 2 | 2 | 1 | gltr | 88 | GMDVIDTGAPLSVPVGGATLGR |  |  |  |  |  |  |  |
| 905 | -3,2 | 5,89 | 2083 | 2 | 2 | 1 | gltr | 88 | GMDVIDTGAPLSVPVGGATLGR |  |  |  |  |  |  |  |
| 1037 | -3,7 | 5,36 | 1955 | 1,5 | 2 | 1 | tlgr | 110 | IFNVLGEPVDNLGPVDTR |  |  |  |  |  |  |  |
| 1621 | -1,6 | 5,63 | 1472 | 1 | 2 | 1 | gvgk | 179 | TVLIMELINNIAK |  |  |  |  |  |  |  |
| 1398 | -1,3 | 5,6 | 1488 | 2 | 2 | 1 | gvgk | 179 | TVLIMELINNIAK |  |  |  |  |  |  |  |
| 612 | -2,7 | 5,89 | 1602 | 2,3 | 2 | 1 | tesk | 232 | VALVYGQMNEPPGAR |  |  |  |  |  |  |  |
| 1249 | -3,4 | 6,3 | 1472 | 1,6 | 2 | 1 | armr | 249 | VGLTALTMAEYFR |  |  |  |  |  |  |  |
| 1012 | -1,5 | 5,65 | 1488 | 0,3 | 2 | 1 | armr | 249 | VGLTALTMAEYFR |  |  |  |  |  |  |  |
| 953 | -3 | 6,35 | 1434 | 1 | 2 | 1 | nifr | 278 | FVQAGSEVSALLGR |  |  |  |  |  |  |  |
| 963 | -2,8 | 6,03 | 1434 | 2 | 2 | 1 | nifr | 278 | FVQAGSEVSALLGR |  |  |  |  |  |  |  |
| **2** | **-52** | **6,9** | **3,2** | **3** | **1** | **1** | **53,6** | **tr\|H2CPP4\|H2CPP4_COLES** | **EC 3.6.3.14; ATP synthase F1 sector subunit beta;** |  |  |  |  |  |  |  |
| spectrum | log(e) | log(I) | m+h | delta | z | zeta | pre | Start | sequence |  |  |  |  |  |  |  |
| 1231 | -2,5 | 5,53 | 1735 | 0,9 | 2 | 1 | nlgr | 23 | IAQIIGPVLDVAFPPGK |  |  |  |  |  |  |  |
| 1037 | -3,7 | 5,36 | 1955 | 1,5 | 2 | 1 | tlgr | 110 | IFNVLGEPVDNLGPVDTR |  |  |  |  |  |  |  |
| 1621 | -1,6 | 5,63 | 1472 | 1 | 2 | 1 | gvgk | 179 | TVLIMELINNIAK |  |  |  |  |  |  |  |
| 1398 | -1,3 | 5,6 | 1488 | 2 | 2 | 1 | gvgk | 179 | TVLIMELINNIAK |  |  |  |  |  |  |  |
| 612 | -2,7 | 5,89 | 1602 | 2,3 | 2 | 1 | aesk | 232 | VALVYGQMNEPPGAR |  |  |  |  |  |  |  |
| 1249 | -3,4 | 6,3 | 1472 | 1,6 | 2 | 1 | armr | 249 | VGLTALTMAEYFR |  |  |  |  |  |  |  |
| 1012 | -1,5 | 5,65 | 1488 | 0,3 | 2 | 1 | armr | 249 | VGLTALTMAEYFR |  |  |  |  |  |  |  |
| 953 | -3 | 6,35 | 1434 | 1 | 2 | 1 | nifr | 278 | FVQAGSEVSALLGR |  |  |  |  |  |  |  |
| 963 | -2,8 | 6,03 | 1434 | 2 | 2 | 1 | nifr | 278 | FVQAGSEVSALLGR |  |  |  |  |  |  |  |
| **3** | **-32** | **6,71** | **9,7** | **18** | **4** | **5** | **55,3** | **sp\|A9LYH0\|ATPA_ACOAM** | **EC 3.6.3.14; ATP synthase F1 sector subunit alpha;** |  |  |  |  |  |  |  |
| spectrum | log(e) | log(I) | m+h | delta | z | zeta | pre | Start | sequence |  |  |  |  |  |  |  |
| 758 | -4,9 | 5,98 | 1599 | 0,9 | 2 | 1 | revk | 26 | VVNTGTVLQVGDGIAR |  |  |  |  |  |  |  |
| 760 | -2,7 | 5,93 | 1599 | 1,1 | 2 | 1 | revk | 26 | VVNTGTVLQVGDGIAR |  |  |  |  |  |  |  |
| 762 | -2,5 | 6,1 | 1417 | 0,6 | 2 | 1 | atgr | 95 | IAQIPVSEAYLGR |  |  |  |  |  |  |  |
| 624 | -2,6 | 6,18 | 1253 | 1 | 2 | 1 | sefr | 129 | LIESPAPGIISR |  |  |  |  |  |  |  |
| 475 | -2,1 | 5,75 | 1275 | -0,9 | 2 | 1 | qtgk | 177 | TAVATDTILNQK |  |  |  |  |  |  |  |
| **4** | **-12** | **5,83** | **7** | **8** | **2** | **2** | **48,1** | **sp\|P42895\|ENO2_MAIZE** | **EC 4.2.1.11; 2-phospho-D-glycerate hydro-lyase 2;** |  |  |  |  |  |  |  |
| spectrum | log(e) | log(I) | m+h | delta | z | zeta | pre | Start | sequence |  |  |  |  |  |  |  |
| 933 | -5,5 | 5,49 | 1791 | -0,6 | 2 | 1 | tfar | 36 | AAVPSGASTGVYEALELR |  |  |  |  |  |  |  |
| 789 | -1,1 | 5,57 | 1574 | -0,2 | 2 | 1 | lllk | 355 | VNQIGSVTESIEAVK |  |  |  |  |  |  |  |
| **5** | **-10** | **6,29** | **4,7** | **6** | **2** | **3** | **59,1** | **sp\|P19023\|ATPBM_MAIZE** | **ATP synthase subunit beta, mitochondrial; EC 3.6.3.14;** |  |  |  |  |  |  |  |
| spectrum | log(e) | log(I) | m+h | delta | z | zeta | pre | Start | sequence |  |  |  |  |  |  |  |
| 639 | -2,3 | 5,86 | 1410 | 0,8 | 2 | 1 | rgqr | 147 | VLNTGSPITVPVGR |  |  |  |  |  |  |  |
| 669 | -2,1 | 5,54 | 1410 | 1,7 | 2 | 1 | rgqr | 147 | VLNTGSPITVPVGR |  |  |  |  |  |  |  |
| 872 | -3,2 | 5,93 | 1493 | 1,5 | 2 | 1 | nifr | 335 | FTQANSEVSALLGR |  |  |  |  |  |  |  |
| **6** | **-9,5** | **6,61** | **4,4** | **10** | **2** | **3** | **50,4** | **tr\|Q6L9Z6\|Q6L9Z6_9LILI** | **RuBisCO large subunit;** |  |  |  |  |  |  |  |
| spectrum | log(e) | log(I) | m+h | delta | z | zeta | pre | start | sequence |  |  |  |  |  |  |  |
| 540 | -2,6 | 6,06 | 1408 | 1,1 | 2 | 1 | kdyk | 15 | LTYYTPEYETK |  |  |  |  |  |  |  |
| 626 | -2 | 6,24 | 1231 | 0,4 | 2 | 1 | negr | 429 | DLATEGNEIIR |  |  |  |  |  |  |  |
| 629 | -1,2 | 6,09 | 1231 | 0,1 | 2 | 1 | negr | 429 | DLATEGNEIIR |  |  |  |  |  |  |  |
| **7** | **-3,5** | **5,72** | **2** | **2** | **1** | **2** | **63,8** | **tr\|Q6ZFJ9\|Q6ZFJ9_ORYSJ** | **60 kDa chaperonin beta subunit; Os02g0102900 protein;** |  |  |  |  |  |  |  |
| spectrum | log(e) | log(I) | m+h | delta | z | zeta | pre | start | sequence |  |  |  |  |  |  |  |
| 389 | -3,5 | 5,55 | 1296 | 1,3 | 2 | 1 | egvk | 158 | VVAAGANPVQITR |  |  |  |  |  |  |  |
| 400 | -2,5 | 5,23 | 1296 | 1,3 | 2 | 1 | egvk | 158 | VVAAGANPVQITR |  |  |  |  |  |  |  |
| **12** | **-1,5** | **5,34** | **2,1** | **3** | **1** | **1** | **61,4** | **tr\|Q7X9A7\|Q7X9A7_ORYSJ** | **60 kDa chaperonin alpha subunit;** |  |  |  |  |  |  |  |
| spectrum | log(e) | log(I) | m+h | delta | z | zeta | pre | start | sequence |  |  |  |  |  |  |  |
| 671 | -1,6 | 5,34 | 1480 | -0,1 | 2 | 1 | eidr | 238 | GYISPQFVTNPEK |  |  |  |  |  |  |  |
| **1** | **-23** | **6,3** | **8,8** | **11** | **3** | **4** | **49,8** | **tr\|B6STH5\|B6STH5_MAIZE** | **Phosphoglycerate kinase; EC 2.7.2.3;** |  |  |  |  |  |  |  |
| spectrum | log(e) | log(I) | m+h | delta | z | zeta | pre | start | sequence |  |  |  |  |  |  |  |
| 1156 | -4,1 | 5,88 | 1748 | 1,5 | 2 | 1 | evek | 180 | LVAALPNGGVLLLENVR |  |  |  |  |  |  |  |
| 411 | -1,8 | 5,52 | 1103 | -0,2 | 2 | 1 | sspk | 266 | RPFAAIVGGSK |  |  |  |  |  |  |  |
| 408 | -1,3 | 5,49 | 1103 | 0,6 | 2 | 1 | sspk | 266 | RPFAAIVGGSK |  |  |  |  |  |  |  |
| 631 | -4,8 | 5,77 | 1574 | -0,2 | 2 | 1 | lsgk | 424 | GVTTIIGGGDSVAAVEK |  |  |  |  |  |  |  |
| **2** | **-21** | **6,3** | **2,5** | **3** | **1** | **1** | **31,4** | **tr\|Q1ENY9\|Q1ENY9_MUSAC** | **Phosphoglycerate kinase, chloroplast, putative; EC 2.7.2.3;** |  |  |  |  |  |  |  |
| spectrum | log(e) | log(I) | m+h | delta | z | zeta | pre | start | sequence |  |  |  |  |  |  |  |
| 753 | -2,5 | 5,88 | 1405 | 1,8 | 2 | 1 | llqk | 252 | ELDYLVGAVSNPK |  |  |  |  |  |  |  |
| 411 | -1,8 | 5,52 | 1103 | -0,2 | 2 | 1 | snpk | 265 | RPFAAIVGGSK |  |  |  |  |  |  |  |
| 408 | -1,3 | 5,49 | 1103 | 0,6 | 2 | 1 | snpk | 265 | RPFAAIVGGSK |  |  |  |  |  |  |  |
| 631 | -4,8 | 5,77 | 1574 | -0,2 | 2 | 1 | lsgk | 423 | GVTTIIGGGDSVAAVEK |  |  |  |  |  |  |  |
| **3** | **-11** | **6,12** | **4** | **4** | **1** | **1** | **0** | **tr\|Q655T1\|Q655T1_ORYSJ** | **no protein information available** |  |  |  |  |  |  |  |
| spectrum | log(e) | log(I) | m+h | delta | z | zeta | pre | start | sequence |  |  |  |  |  |  |  |
| 1161 | -1,1 | 5,86 | 1751 | 0,8 | 2 | 1 | evqk | 106 | LAATLPDGGVLLLENVR |  |  |  |  |  |  |  |
| 631 | -4,8 | 5,77 | 1574 | -0,2 | 2 | 1 | itak | 350 | GVTTIIGGGDSVAAVEK |  |  |  |  |  |  |  |
| **4** | **-11** | **6,7** | **14** | **19** | **2** | **3** | **20,8** | **tr\|F8UCA0\|F8UCA0_9LILI** | **Glyceraldehyde-3-phosphate dehydrogenase; EC 1.2.1.12;** |  |  |  |  |  |  |  |
| spectrum | log(e) | log(I) | m+h | delta | z | zeta | pre | start | sequence |  |  |  |  |  |  |  |
| 865 | -2,1 | 5,35 | 1744 | -0,9 | 2 | 1 | apsk | 29 | DAPMFVMGVNEDQYK |  |  |  |  |  |  |  |
| 925 | -3,7 | 6,47 | 1499 | 1,3 | 2 | 1 | mafr | 137 | VPTVDVSVVDLTVR |  |  |  |  |  |  |  |
| 934 | -3,2 | 6,27 | 1499 | 0,7 | 2 | 1 | mafr | 137 | VPTVDVSVVDLTVR |  |  |  |  |  |  |  |
| **5** | **-9,9** | **6,73** | **3,9** | **4** | **1** | **1** | **20,1** | **tr\|Q7FAH2\|Q7FAH2_ORYSJ** | **Glyceraldehyde-3-phosphate dehydrogenase 2, cytosolic; EC 1.2.1.12** |  |  |  |  |  |  |  |
| spectrum | log(e) | log(I) | m+h | delta | z | zeta | pre | start | sequence |  |  |  |  |  |  |  |
| 925 | -3,7 | 6,47 | 1499 | 1,3 | 2 | 1 | mafr | 237 | VPTVDVSVVDLTVR |  |  |  |  |  |  |  |
| 934 | -3,2 | 6,27 | 1499 | 0,7 | 2 | 1 | mafr | 237 | VPTVDVSVVDLTVR |  |  |  |  |  |  |  |
| 945 | -1,3 | 5,73 | 1762 | 0,7 | 2 | 1 | nfvk | 312 | LVSWYDNEWGYSSR |  |  |  |  |  |  |  |
| **6** | **-2,9** | **6,05** | **5,1** | **8** | **1** | **1** | **33** | **sp\|P27337\|PER1_HORVU** | **Peroxidase 1; EC 1.11.1.7;** |  |  |  |  |  |  |  |
| spectrum | log(e) | log(I) | m+h | delta | z | zeta | pre | start | sequence |  |  |  |  |  |  |  |
| 1017 | -2,9 | 6,05 | 1711 | 1,2 | 2 | 1 | vaar | 123 | DSVVALGGPSWTVPLGR |  |  |  |  |  |  |  |
| **7** | **-2,1** | **5,39** | **3,2** | **5** | **1** | **1** | **42,7** | **tr\|F2D714\|F2D714_HORVD** | **Predicted protein;** |  |  |  |  |  |  |  |
| spectrum | log(e) | log(I) | m+h | delta | z | zeta | pre | start | sequence |  |  |  |  |  |  |  |
| 959 | -2,1 | 5,39 | 1787 | 0,6 | 2 | 1 | dmvk | 375 | VIAWYDNEWGYSQR |  |  |  |  |  |  |  |
| **11** | **-1,5** | **5,86** | **3,3** | **8** | **1** | **2** | **50,1** | **sp\|P25776\|ORYA_ORYSJ** | **no protein information available** |  |  |  |  |  |  |  |
| spectrum | log(e) | log(I) | m+h | delta | z | zeta | pre | start | sequence |  |  |  |  |  |  |  |
| 509 | -1,5 | 5,61 | 1539 | 1,3 | 2 | 1 | slqk | 254 | AVANQPVSVAIEAGGR |  |  |  |  |  |  |  |
| 505 | -1,2 | 5,5 | 1539 | 2,5 | 2 | 1 | slqk | 254 | AVANQPVSVAIEAGGR |  |  |  |  |  |  |  |
| **1** | **-9,4** | **6,23** | **9,8** | **12** | **2** | **4** | **34,4** | **tr\|F2CRK1\|F2CRK1_HORVD** | **Predicted protein;** |  |  |  |  |  |  |  |
| spectrum | log(e) | log(I) | m+h | delta | z | zeta | pre | start | sequence |  |  |  |  |  |  |  |
| 754 | -2,3 | 5,68 | 1761 | 1,1 | 2 | 1 | feek | 186 | DGIDYAAVTVQLPGGER |  |  |  |  |  |  |  |
| 345 | -2,2 | 5,87 | 1563 | 1 | 2 | 1 | pkgr | 242 | GGSTGYDNAVALPAGGR |  |  |  |  |  |  |  |
| 292 | -1,8 | 5,35 | 1563 | 2,1 | 2 | 1 | pkgr | 242 | GGSTGYDNAVALPAGGR |  |  |  |  |  |  |  |
| 354 | -1,7 | 5,38 | 1563 | 2,3 | 2 | 1 | pkgr | 242 | GGSTGYDNAVALPAGGR |  |  |  |  |  |  |  |
| **2** | **-4,9** | **5,9** | **3,2** | **5** | **1** | **1** | **41** | **tr\|Q0DJC0\|Q0DJC0_ORYSJ** | **Os05g0302700 protein;** |  |  |  |  |  |  |  |
| spectrum | log(e) | log(I) | m+h | delta | z | zeta | pre | start | sequence |  |  |  |  |  |  |  |
| 437 | -4,9 | 5,9 | 1192 | -0,4 | 2 | 1 | nilr | 353 | AVAGAGVLAGYDK |  |  |  |  |  |  |  |
| **3** | **-4,6** | **5,6** | **8,5** | **12** | **1** | **1** | **24,7** | **tr\|F2DTJ2\|F2DTJ2_HORVD** | **Predicted protein** |  |  |  |  |  |  |  |
| Spectrum | log(e) | log(I) | m+h | delta | z | zeta | pre | start | sequence |  |  |  |  |  |  |  |
| 1184 | -4,6 | 5,6 | 2233 | 1,5 | 2 | 1 | flar | 51 | NPFGQVPVLEDGDLTLFESR |  |  |  |  |  |  |  |
| **4** | **-3,4** | **5,75** | **5,1** | **8** | **1** | **1** | **33** | **sp\|P27337\|PER1_HORVU** | **Peroxidase 1; EC 1.11.1.7;** |  |  |  |  |  |  |  |
| spectrum | log(e) | log(I) | m+h | delta | z | zeta | pre | start | sequence |  |  |  |  |  |  |  |
| 918 | -3,4 | 5,75 | 1711 | 0,5 | 2 | 1 | vaar | 123 | DSVVALGGPSWTVPLGR |  |  |  |  |  |  |  |
| **7** | **-2** | **5,65** | **5,4** | **7** | **1** | **1** | **29,8** | **tr\|G0YLW6\|G0YLW6_9ARAE** | **Putative chlorophyll a/b binding protein;** |  |  |  |  |  |  |  |
| spectrum | log(e) | log(I) | m+h | delta | z | zeta | pre | start | sequence |  |  |  |  |  |  |  |
| 974 | -2 | 5,65 | 1697 | -0,1 | 2 | 1 | vwfk | 149 | TGALLLDGNTLNYFGK |  |  |  |  |  |  |  |
| **1** | **-2,7** | **6,22** | **3,8** | **6** | **1** | **2** | **24,8** | **sp\|P13192\|PSAF_HORVU** | **Light-harvesting complex I 17 kDa protein; PSI-F;** |  |  |  |  |  |  |  |
| spectrum | log(e) | log(I) | m+h | delta | z | zeta | pre | start | sequence |  |  |  |  |  |  |  |
| 1152 | -2,7 | 6,08 | 1180 | 1 | 2 | 1 | iipr | 210 | GFIWPVAAYR |  |  |  |  |  |  |  |
| 1162 | -2,7 | 5,65 | 1180 | 0,7 | 2 | 1 | iipr | 210 | GFIWPVAAYR |  |  |  |  |  |  |  |
| **2** | **-2,4** | **5,44** | **5,7** | **9** | **1** | **1** | **27,7** | **tr\|Q6WFB1\|Q6WFB1_MAIZE** | **Photosystem II subunit PsbS;** |  |  |  |  |  |  |  |
| spectrum | log(e) | log(I) | m+h | delta | z | zeta | pre | start | sequence |  |  |  |  |  |  |  |
| 1004 | -2,4 | 5,44 | 1585 | 1,3 | 2 | 1 | pkpk | 73 | VEDGIFGTSGGIGFTK |  |  |  |  |  |  |  |
| **1** | **-1,5** | **5,48** | **13** | **28** | **1** | **2** | **9,4** | **sp\|A1EA25\|PSBE_AGRST** | **Cytochrome b559 subunit alpha; PSII reaction center subunit V;** |  |  |  |  |  |  |  |
| spectrum | log(e) | log(I) | m+h | delta | z | zeta | pre | start | sequence |  |  |  |  |  |  |  |
| 1002 | -1,5 | 5,05 | 1486 | 1,8 | 2 | 1 | itdr | 70 | FDSLEQLDEFSR |  |  |  |  |  |  |  |
| 997 | -1,2 | 5,28 | 1486 | 0,5 | 2 | 1 | itdr | 70 | FDSLEQLDEFSR |  |  |  |  |  |  |  |
|  |  |  |  |  |  |  |  |  |  |  |  |  |  |  |  |  |
| **rank**: the relative position of a protein in the list. This can change, depending on how the data is sorted. | | | | | | | | | | | | | |  |  |  |
| **log(e)**: the base-10 log of the expectation that any particular protein assignment was made at random (*E*-value). | | | | | | | | | | | | | |  |  |  |
| **log(I)**: the base-10 log of the sum of the fragment ion intensities in the tandem mass spectra used to make this assignment. | | | | | | | | | | | | | |  |  |  |
| **% (measured)**:the amino acid coverage of the protein in this assignment | | | | | | | | | | | |  |  |  |  |  |
| **%(corrected)**: the amino acid coverage of the protein in this assignment / the coverage corrected for peptide sequences that are unlikely to  be observed using normal proteomics methods. | | | | | | | | | | | | | | | | |
| **unique**: the number of unique peptide sequences associated with this protein assignment. | | | | | | | | | | | | | |  |  |  |
| **total**: the total number of tandem mass spectra that can be assigned to this protein. | | | | | | | | | | | | |  |  |  |  |
| **Mr**: the molecular mass of the protein sequence, in kiloDaltons. | | | | | | | | | | |  |  |  |  |  |  |
